# Conjugates of Tacrine and Salicylic Acid Derivatives as New Promising Multitarget Agents for Alzheimer’s Disease

**DOI:** 10.3390/ijms24032285

**Published:** 2023-01-24

**Authors:** Galina F. Makhaeva, Nadezhda V. Kovaleva, Elena V. Rudakova, Natalia P. Boltneva, Maria V. Grishchenko, Sofya V. Lushchekina, Tatiana Y. Astakhova, Olga G. Serebryakova, Elena N. Timokhina, Ekaterina F. Zhilina, Evgeny V. Shchegolkov, Mariya V. Ulitko, Eugene V. Radchenko, Vladimir A. Palyulin, Yanina V. Burgart, Victor I. Saloutin, Sergey O. Bachurin, Rudy J. Richardson

**Affiliations:** 1 Institute of Physiologically Active Compounds at Federal Research Center of Problems of Chemical Physics and Medicinal Chemistry, Russian Academy of Sciences, Chernogolovka 142432, Russia; 2 Postovsky Institute of Organic Synthesis, Urals Branch of Russian Academy of Sciences, Yekaterinburg 620990, Russia; 3 Emanuel Institute of Biochemical Physics Russian Academy of Sciences, Moscow 119334, Russia; 4 Institute of Natural Sciences and Mathematics of the Ural Federal University Named after the First President of Russia B. N. Yeltsin, Ekaterinburg 620083, Russia; 5 Department of Chemistry, Lomonosov Moscow State University, Moscow 119991, Russia; 6 Department of Environmental Health Sciences, University of Michigan, Ann Arbor, MI 48109, USA; 7 Department of Neurology, University of Michigan, Ann Arbor, MI 48109, USA; 8 Center of Computational Medicine and Bioinformatics, University of Michigan, Ann Arbor, MI 48109, USA; 9 Michigan Institute for Computational Discovery and Engineering, University of Michigan, Ann Arbor, MI 48109, USA

**Keywords:** Alzheimer’s disease, acetylcholinesterase, butyrylcholinesterase, salicylic acid derivatives, tacrine conjugates, propidium displacement, Aβ_42_ self-aggregation, antioxidant activity, metal chelation, ADMET prediction

## Abstract

A series of previously synthesized conjugates of tacrine and salicylamide was extended by varying the structure of the salicylamide fragment and using salicylic aldehyde to synthesize salicylimine derivatives. The hybrids exhibited broad-spectrum biological activity. All new conjugates were potent inhibitors of acetylcholinesterase (AChE) and butyrylcholinesterase (BChE) with selectivity toward BChE. The structure of the salicylamide moiety exerted little effect on anticholinesterase activity, but AChE inhibition increased with spacer elongation. The most active conjugates were salicylimine derivatives: IC_50_ values of the lead compound **10c** were 0.0826 µM (AChE) and 0.0156 µM (BChE), with weak inhibition of the off-target carboxylesterase. The hybrids were mixed-type reversible inhibitors of both cholinesterases and displayed dual binding to the catalytic and peripheral anionic sites of AChE in molecular docking, which, along with experimental results on propidium iodide displacement, suggested their potential to block AChE-induced *β*-amyloid aggregation. All conjugates inhibited Aβ_42_ self-aggregation in the thioflavin test, and inhibition increased with spacer elongation. Salicylimine **10c** and salicylamide **5c** with (CH_2_)_8_ spacers were the lead compounds for inhibiting Aβ_42_ self-aggregation, which was corroborated by molecular docking to Aβ_42_. ABTS^•+^-scavenging activity was highest for salicylamides **5a–c**, intermediate for salicylimines **10a–c**, low for F-containing salicylamides **7**, and non-existent for methoxybenzoylamides **6** and difluoromethoxybenzoylamides **8**. In the FRAP antioxidant (AO) assay, the test compounds displayed little or no activity. Quantum chemical analysis and molecular dynamics (MD) simulations with QM/MM potentials explained the AO structure–activity relationships. All conjugates were effective chelators of Cu^2+^, Fe^2+^, and Zn^2+^, with molar compound/metal (Cu^2+^) ratios of 2:1 (**5b**) and ~1:1 (**10b**). Conjugates exerted comparable or lower cytotoxicity than tacrine on mouse hepatocytes and had favorable predicted intestinal absorption and blood-brain barrier permeability. The overall results indicate that the synthesized conjugates are promising new multifunctional agents for the potential treatment of AD.

## 1. Introduction

Alzheimer’s disease (AD) is a progressive and fatal degenerative disorder of the central nervous system, leading to the most common form of dementia in the aged population [1,2,3]. AD has a multifactorial causation and results in a wide spectrum of injurious effects, including reduction of acetylcholine levels, accumulation of anomalous β-amyloid (Aβ) and tau proteins, loss of synapses, dysregulation of biometal homeostasis, oxidative stress, and neuronal cell death [4]. In response to the multifunctional properties of AD, the design of multitarget directed ligands (MTDLs) has emerged as a widely used strategy in the search for new agents to treat this devastating disease [5,6,7].

Currently, cholinergic agents constitute the main pharmacotherapeutic group used for the symptomatic treatment of AD [8,9]. Inhibitors of acetylcholinesterase (AChE, EC 3.1.1.7) increase the activation of cholinergic neurons and promote cognitive functions. Butyrylcholinesterase (BChE, EC 3.1.1.8) inhibition also promotes cognitive improvement, being especially effective during disease progression [10,11]. Compounds inhibiting both cholinesterases are considered to increase AD treatment efficiency [12].

Accumulation of the β-amyloid peptide (Aβ) is one of the key contributing factors in the pathology of AD [13]. Aβ amyloidogenesis is a complex process that involves the sequential formation of different forms of amyloid species, such as monomers, oligomers, protofibrils, and mature fibrils [14]. Soluble oligomeric Aβ, rather than monomers or fibrils, may be the earliest mediators that contribute to the cognitive deficits and neurodegeneration in AD [15,16,17]. Accordingly, compounds that inhibit abnormal Aβ aggregation could have an ameliorative disease-modifying effect [18,19].

Along with its principal function of acetylcholine hydrolysis, AChE has the ability to promote the aggregation of β-amyloid [20] via the participation of its peripheral anionic site (PAS) [21,22]. Recognition of this additional capability has stimulated research to identify AChE inhibitors that can bind both to the catalytic site (CAS) and PAS of AChE [23,24]. Moreover, BChE is also involved in the formation and/or maturation of β-amyloid plaques [25,26,27]. Therefore, according to the anti-amyloid strategy, dual-site inhibitors of AChE as well as BChE inhibitors are of particular interest.

One of the important factors negatively affecting the vital activity of brain neurons is oxidative stress, which is characterized by an imbalance between the formation of reactive oxygen species and their removal through various mechanisms of the AO system [28,29,30]. It was shown that oxidative damage could promote the appearance of amyloid plaques and neurofibrillar tangles in AD brain [31]. Thus, successful protection of neuronal cells from oxidative damage could prevent AD [32]. Accordingly, the creation of cholinesterase inhibitors with additional AO properties is considered as one of the promising goals in the development of anti-AD drugs [33].

Imbalance of the homeostasis of biometals in the brain is another mechanism of AD pathogenesis and progression [34,35,36]. An excess accumulation of transition metals such as Fe^2+^, Cu^2+^, and Zn^2+^ occurs in AD brain. The accumulation of metal ions promotes their binding to β-amyloid, thereby affecting the mechanism and kinetics of Aβ aggregation and stabilizing the neurotoxic amyloid aggregates [35,37]. These metals can also exert neurotoxicity by fostering the overproduction of reactive oxygen species, resulting in increased oxidative stress in neurons [30,32,36]. Therefore, decreasing excess metal concentrations in the brain by chelating agents is one of the rational therapeutic approaches to AD treatment [38,39].

The creation of conjugates has proved to be an auspicious technique for the production of agents that are effective at interacting with multiple biological targets. This approach involves the coupling of two pharmacophores through a linker. In the case of hybrids to be developed as potential therapeutics to counteract the cholinergic deficiency in AD, one of the pharmacophores is an anticholinesterase compound [40], and tacrine has often served in this capacity [41,42,43,44,45,46]. In order to produce hybrids capable of interacting with both the CAS and PAS of AChE, a second pharmacophore is joined to the tacrine moiety through a spacer of appropriate length [47,48].

Additional design features for tacrine-based anti-Alzheimer’s MTDLs include the incorporation of second pharmacophores possessing AO [47,49,50,51,52,53,54] or chelating properties [38,51,55]. It is worth noting that adding free radical scavengers to the tacrine molecule has alleviated the hepatotoxicity that is seen with tacrine on its own [50].

Derivatives of salicylic acid are promising scaffolds to serve as second pharmacophores in the design of multifunctional agents for AD treatment. These moieties possess a wide spectrum of biological activity, including AO and metal-chelating properties, as well as the ability to inhibit β-amyloid aggregation [56,57,58]. Recently, we showed that conjugates of tacrine and salicylamide (**5a–c**) have dual anticholinesterase activity with selectivity toward BChE [59]. Moreover, the hybrid compounds inhibited AChE and BChE two to three times more effectively than tacrine, had the potential to block AChE-induced β-amyloid aggregation (propidium iodide displacement test), exhibited high ABTS^•+^-scavenging activity, and displayed metal-chelating ability.

**Figure 1:**
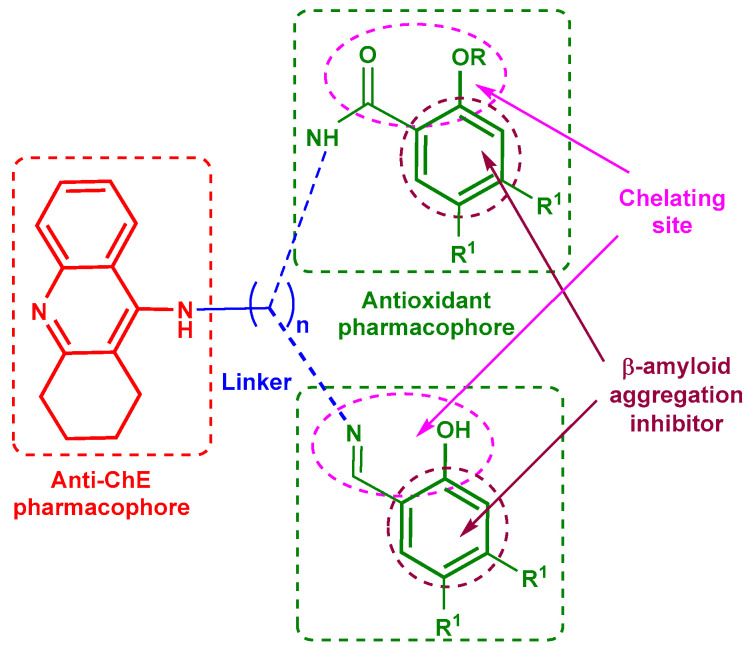
Designed conjugates of tacrine with salicylamide and salicylimine derivatives.

Herein, we extended the variations of the salicylic pharmacophore in the generated hybrid compounds (Figure 1) as potential multifunctional agents for the treatment of AD. In addition, we paid attention to the introduction of a fluorosalicylate fragment, being inspired by the success of the creation of fluorine-containing drugs. Their special attributes are due in part to the presence of fluorine atoms that can favorably modulate the biological properties of molecules. This approach is currently one of the most popular for the creation of new drugs [60,61,62,63,64,65,66,67,68]. To understand the significance of the presence of the hydroxyl group in the aromatic pharmacophore on biological potential, we obtained conjugates with methoxy derivatives of salicylamide. To evaluate the effect of the amide fragment, we also synthesized salicylimines, which were previously prepared only as intermediates [69].

Next, the esterase profile of the conjugates, i.e., their inhibitory activity against cholinesterases and a structurally related enzyme, carboxylesterase (CES, EC 3.1.1.1), was determined, and the results were analyzed using enzyme kinetics and quantum mechanics (QM)-assisted molecular docking. To assess the potential of the hybrids to block AChE-induced aggregation of β-amyloid, their ability to displace propidium iodide from the PAS of AChE was measured. The ability of the compounds to inhibit β-amyloid (1–42) (Aβ_42_) self-aggregation was explored using the thioflavin assay and analyzed using molecular docking. In addition, the primary AO activity of the conjugates was assessed, and the AO structure–activities relationships were examined using quantum chemical analysis and molecular dynamics (MD) simulations with QM/MM potentials. Finally, the ability of the compounds to chelate biometals (Fe^2+^, Cu^2+^, Zn^2+^) was determined, the cytotoxicity of the conjugates against primary mouse hepatocytes was assessed by the MTT assay, and the ADMET profile of the hybrids was computationally predicted.

## 2. Results and Discussion

### 2.1. Chemistry

**Scheme 1:**
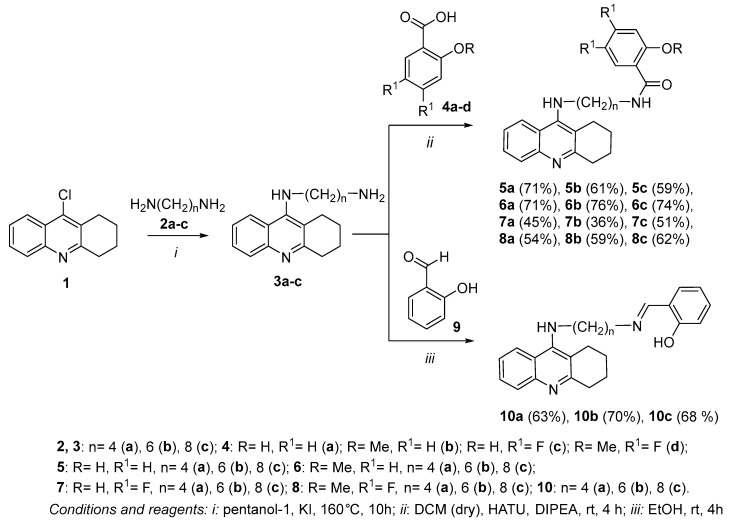
Synthesis of conjugates **5–8a–c** and **10a–c**.

One of the most popular approaches to the synthesis of tacrine conjugates is the synthesis of aminopolymethylene-containing derivatives of tacrine **3** by the substitution of the chlorine atom in 9-chloro-1,2,3,4-tetrahydroacridine **1** with diaminoalkanes [55,70]. Earlier [59], we found that the reaction of tacrine derivatives **3a**–**c** containing 4, 6, and 8 methylene units with salicylic acid **4a** in dry methylene chloride at room temperature in the presence of HATU and DIPEA leads to hybrid compounds of tacrine with salicylamide **5a–c** (Scheme 1). Using this protocol, we synthesized a series of conjugates **6a–c**, **7a–c**, and **8a–c** via reaction of tacrine derivatives **3a–c** with 2-methoxybenzoic **4b**, 4,5-difluorosalicylic **4c**, and 4,5-difluoro-2-methoxybenzoic **4d** acids.

The reaction of compounds **3a–c** with salicylic aldehyde **9** in ethanol at room temperature resulted in conjugates of tacrine and salicylimine **10a–c** having 4, 6, and 8 methylene units in the spacer (Scheme 1). Note that compounds **10a–c** were obtained earlier [69] as intermediates for the synthesis of N^1^-(benzyl)-N^3^-(1,2,3,4-tetrahydroacridin-9-yl)alkyl-1,3-diamines, but at that time, they were not isolated.

The structure of conjugates **5**–**8,10** was confirmed by IR and ^1^H, ^13^C NMR spectroscopy, elemental analysis, and mass spectrometry. The ^19^F NMR spectra were measured for fluorine-containing compounds **7a-c** and **8a-c**.

The IR spectra of compounds **5–8a–c** having the amide function contained characteristic absorption bands of the carbonyl group at ν 1640–1650 cm^−1^ (amide I band). In the IR spectra of conjugates **10a–c** having an imine fragment, the presence of an intense absorption band at ν ~ 1630 cm^−1^ associated with vibrations of the C=N bond were observed. In the ^13^C NMR spectra of compounds **5**–**8a–c**, the signal of an amide carbon atom at δ 163–169 ppm was revealed, whereas the ^13^C NMR spectra of compounds **10a–c** containing a salicylamine fragment were characterized by the presence of two weak-field signals at δ 161 and 165 ppm, corresponding to the phenolic and imine carbon atoms. In addition, the ^1^H NMR spectra of conjugates **10a–c** had a singlet signal of a methyne proton of the N=CH group at δ 8.3 ppm.

### 2.2. Biological Investigations

#### 2.2.1. Inhibition Studies of AChE, BChE, and CES. Structure–Activity Relationships

Assessment of the esterase profile of new potential anti-AD molecules enables the estimation of both the primary pharmacological effects of the tested compounds—their inhibition of AChE and BChE—and their possible unwanted side effect—inhibition of CES, which hydrolyzes numerous ester-containing drugs [71,72,73]. For the esterase profile evaluation, human erythrocyte AChE, equine serum BChE, and porcine liver CES were used. These sources of BChE and CES were employed because of their relatively low cost, high sequence identity to human enzymes [72,74,75], and the exploratory character of this work. The inhibitory activities of the conjugates against the esterases were characterized as IC_50_ values, i.e., the inhibitor concentrations required to decrease a given enzyme activity by 50%, or as the percent inhibition at an inhibitor concentration of 20 µM. Tacrine, an effective AChE and BChE inhibitor, and bis-4-nitrophenyl phosphate (BNPP), a selective CES inhibitor, were used as positive controls. The results are shown in Table 1.

The esterase profile study showed (Table 1) that regardless of the structure of the second pharmacophore, all groups of conjugates effectively inhibited AChE and BChE with IC_50_ values 0.14 to 3.2 times higher than the parent compound tacrine, and, like tacrine, were more selective against BChE. All compounds weakly inhibited CES, an enzyme responsible for the hydrolysis of numerous ester-containing drugs [76]. Low-level inhibition of CES is a desirable trait for anticholinesterase drugs because high-level inhibition of this enzyme may lead to unwanted drug-drug interactions [71,77].

Inhibitory activity toward AChE in all groups of hybrids increased with spacer elongation. Most of the conjugates with spacers ≥ (CH_2_)_6_ were more active than tacrine, with **10c** as a lead compound (IC_50_ = 0.0826 ± 0.0046 µM). Salicylimines **10a–c** were more potent AChE inhibitors in comparison with corresponding salicylamides **5a–c**.

#### 2.2.2. Kinetic Studies of AChE and BChE Inhibition

**Figure 2:**
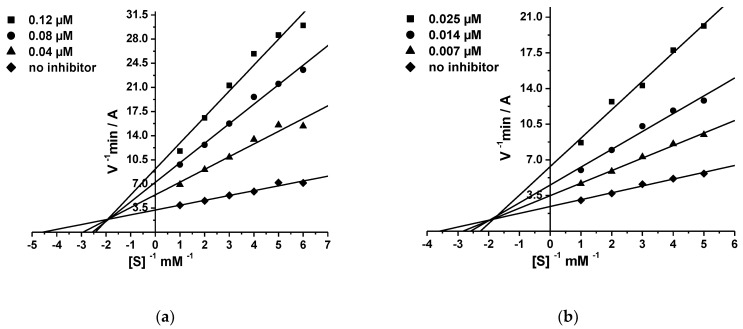
Lineweaver–Burk double-reciprocal plots of steady-state inhibition of AChE (**a**) and BChE (**b**) by compound **10c**. Each plot indicates mixed-type inhibition.

The inhibition kinetics of AChE and BChE was studied for all five groups of compounds using conjugates **5c**, **6c**, **7c**, **8c**, and **10c** with spacer (CH_2_)_8_. As a sample, Figure 2 shows Lineweaver–Burk analysis for AChE and BChE inhibition data by compound **10c**. The graphical analysis of the kinetic data on AChE and BChE inhibition by this compound (Figure 2a,b) demonstrated changes in both *K*_m_ and *V*_max_, attesting to a mixed type of inhibition. Similar results were obtained for other tested compounds (Figures S61 and S62). The obtained values of inhibition constants (*K*_i_ = competitive component and α*K*_i_ = non-competitive component) are shown in Table 2.

#### 2.2.3. Molecular Modeling Studies

**Figure 3:**
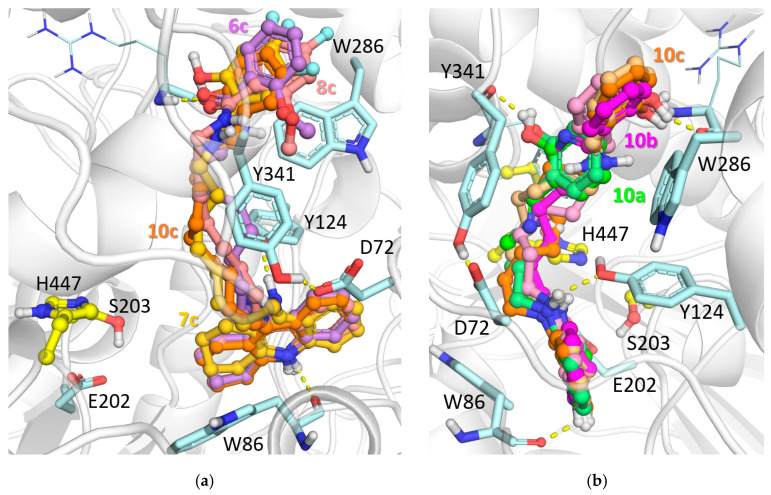
Molecular docking to the AChE active site of conjugates **6c** (carbon atoms are colored lilac blue), **7c** (carbon atoms are colored yellow), **8c** (carbon atoms are colored pink), **10c** (carbon atoms are colored orange) (**a**); and binding of non-protonated and protonated forms of **10a–c** (carbon atoms of compound **10a** are colored green, **10b**—magenta, **10c**—orange); carbon atoms of protonated forms are colored with a paler shade (**b**).

The mode of binding of salicylamides **5** was reported earlier [59]: the protonated tacrine fragment was bound in the active site, and the salicylamide group was bound in the PAS, advancing into it with increasing linker length. For the newly synthesized groups of compounds **6**, **7**, **8**, and **10**, this mode of binding was preserved (Figure 3a). For all groups, the protonated endocyclic nitrogen atom of the tacrine fragment was hydrogen-bonded with the Trp86 main chain oxygen atom. Moreover, as for compounds **5**, growth of the linker led to better occupation of the PAS by the salicyl group. In the case of the methoxy derivatives **6** and **8**, the hydrogen bond with the Arg296 main chain NH-group was formed by the amide oxygen, while for salicylates it was formed by the hydroxy group. The imine group of the salicylimines **10** was found to have a p*K*a of about 6.3–6.9, indicating that a small fraction of the inhibitors **10a–c** could have a protonated spacer. In the case of protonated imine spacers, the compounds had an intramolecular hydrogen bond. Nevertheless, protonation of the spacer had little effect on the binding mode of the compounds (Figure 3b). 

**Figure 4:**
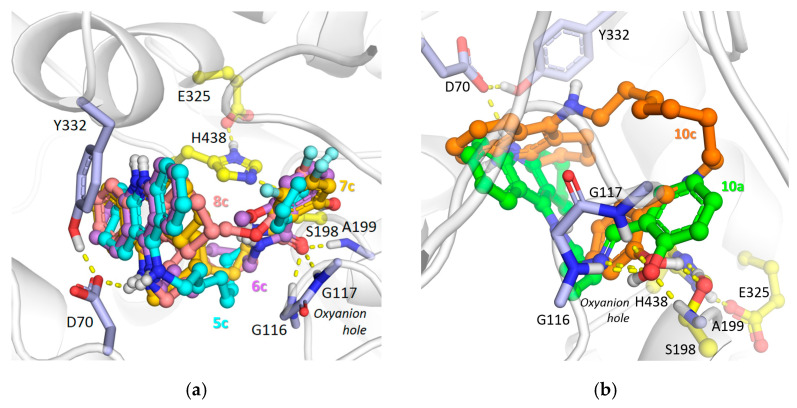
Molecular docking to the BChE active site of conjugates **5c** (carbon atoms are colored cyan), **6c** (carbon atoms are colored lilac blue), **7c** (carbon atoms are colored yellow), **8c** (carbon atoms are colored pink) (**a**); and conjugates **10a** (carbon atoms are colored green) and **10c** (carbon atoms are colored orange) (**b**).

In the case of binding to BChE, due to its wide gorge, there was not much difference in the binding modes for the compounds **5**–**8**, which were found to bind with the salicylamide group in the active site. The amide oxygen atom was bound to the oxyanion hole, and the phenolic groups formed additional hydrogen bonds with the catalytic Ser198 side chain. The positively charged tacrine fragment occupied the PAS and formed ionic interactions with Asp70 and aromatic ring stacking with the Tyr332 side chain (Figure 4a). In the case of the salicylimine compound **10a** with the shortest linker, the intramolecular hydrogen bond apparently impaired this binding, but with an increase of the linker length, the salicylimine group was better accommodated in the active site with hydrogen bonding to Ser198 (Figure 4b).

#### 2.2.4. Displacement of Propidium Iodide from the PAS of *Ee*AChE

Considering the ability of the AChE peripheral anionic site (PAS) to induce β-amyloid aggregation [20,22,78], we evaluated conjugates **5–8, 10** for their propensity to displace the selective PAS ligand, propidium iodide, from the *Ee*AChE PAS. This method is widely used as a screening approach to assess the potential ability of compounds to block AChE-induced β-amyloid aggregation. This is because when the compounds bind to the AChE PAS, they prevent the binding of β-amyloid, thereby inhibiting its aggregation [23,44,79,80,81,82]. Donepezil, a mixed-type AChE inhibitor for which the ability to block AChE-PAS-induced Aβ aggregation has been demonstrated [79], was used as a positive control and as a reference compound. The results are presented in Table 1.

It can be seen (Table 1) that the first four groups of conjugates containing the amide fragment—salicylamides **5a–c**, methoxybenzoylamides **6a–c**, difluorosalicylamides **7a–c**, and methoxydifluorobenzoylamides **8a–c**—were able to bind to the PAS of *Ee*AChE and displace propidium iodide at similar levels (10–15%) to that of the reference compound donepezil (11.9 ± 0.9%). In contrast, salicylimines **10a–c** more effectively displaced propidium iodide (18–19%) from the AChE PAS than donepezil.

Taken together, the results from propidium iodide displacement, kinetics, and molecular docking indicate that the compounds are double-site binding AChE inhibitors, which bind to the PAS of AChE and, therefore, have the potential to block the AChE-induced aggregation of β-amyloid.

#### 2.2.5. Inhibition of β-Amyloid (1–42) (Aβ_42_) Self-Aggregation

The inhibitory activity of tested hybrids against the self-aggregation of Aβ_42_ was determined in vitro using the thioflavin T (ThT) fluorimetric assay [79,83]. According to [84], the ThT test is ideal for preliminary screening of inhibitors of Aβ_42_ self-aggregation, as it enables the quantitative testing of large numbers of compounds within a relatively short time.

The results presented in Table 1 demonstrate that all hybrids exhibited inhibitory activity against Aβ_42_ self-aggregation. Moreover, the degree of Aβ_42_ self-aggregation inhibition depended on the structure of the second pharmacophore, a salicylic acid derivative, and on the length of the spacer. The most active group was the salicylimines **10a–c**, which inhibited Aβ_42_ self-aggregation in the range from 57.9% to 80.1%. The replacement of the imine fragment by an amide moiety (salicylamides **5a–c**) led to some decrease in the inhibitory activity (20.7% to 71.9%). Other groups of compounds, i.e., methoxybenzoylamides **6a–c**, difluorosalicylamides **7a–c**, and methoxydifluorobenzoylamides **8a–c**, demonstrated a further tendency toward a decrease in the degree of Aβ_42_ self-aggregation inhibition (12.3% to 55.6%). It is also important to note that inhibitory activity toward Aβ_42_ self-aggregation in all groups of hybrids increased with elongation of the spacer from (CH_2_)_4_ to (CH_2_)_8_. The most active inhibitors of Aβ_42_ self-aggregation were salicylimine **10c** (80.1 ± 5.6%) and salicylamide **5c** (71.9 ± 5.7%) with a spacer length (CH_2_)_8_, which exhibited inhibitory activity against Aβ_42_ self-aggregation comparable to the level of the reference compound Myricetin (74.7 ± 5.2%).

#### 2.2.6. Molecular Modeling: Interactions of Conjugates with Aβ_42_

As described above, in the present study, inhibition of Aβ_42_ self-aggregation was monitored by a fluorescence spectroscopy method using thioflavin T. This procedure reveals the formation of β-folded structures that form toxic Aβ_42_ oligomers and fibrils from initial α-helical soluble forms. For this reason, molecular docking was carried out using a structure of the α-helical soluble Aβ_42_ monomer as the target. The available structure PDB ID 1IYT [85] was obtained by NMR spectroscopy and contained ten conformations of the monomer. This structure is actively used in computational studies as a model for the soluble monomeric form of Aβ_42_. However, in the relevant publications, it is most often indicated (or it follows from the presented results) that only one out of the ten conformers was used for molecular docking: either the first or the sixth one, which has the least deviation from other conformers [86]. In some publications it is not clearly indicated which particular conformer was taken, and it can only be surmised that it was the first conformer.

In the present investigation, we carried out molecular docking using all conformers of Aβ_42_ as the targets. According to the molecular docking data, binding of compounds to various conformers of Aβ_42_ differed considerably. The strongest binding was obtained for compound **10c** docked to the second conformer of Aβ_42_. This compound also showed the best ability to inhibit Aβ_42_ self-aggregation experimentally (Table 1).

**Figure 5:**
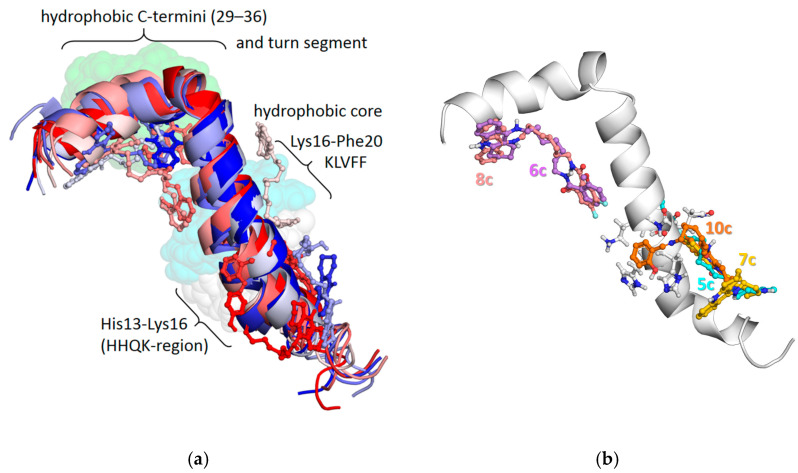
Results of molecular docking of compound **10c** to all 10 conformers of an α-helical soluble monomeric form of Aβ_42_ (PDB ID 1IYT; solution NMR structure). The color gradually changes from red through white to blue from the first to the tenth Aβ_42_ conformer) (**a**); and of compounds **5c** (carbon atoms are colored cyan), **6c** (carbon atoms are colored lilac blue), **7c** (carbon atoms are colored yellow), **8c** (carbon atoms are colored pink), and **10c** (carbon atoms are colored orange) to the second conformer of Aβ_42_ (**b**).

Binding of compound **10c** to Aβ_42_ differed substantially among the conformers (Figure 5a). Regions of the Aβ_42_ monomer important for self-aggregation can be summarized as follows: the critical regions on Aβ_42_ that are able to initiate nucleation, conformational transition, and fibril formation are the central hydrophobic core (K16LVFF20), hydrophobic C-terminal residues (29–36), and the turn segment [87]. Furthermore, the His13–Lys16 sub-region (HHQK domain) was shown to be a key motif in binding of glycosaminoglycans, which facilitate transition of an α-helix to a more stable β-sheet conformation associated with neurotoxicity [88,89]. Among the best binding modes of **10c** to the different conformers available from PDB ID 1IYT, binding to the first conformer affected these sub-regions zones least of all, while binding to the second conformer had the most contacts to the HHQK domain. Comparing the binding of compound **10c** with that of the other conjugates with the longest linker (n = 8), in the case of binding to the second conformer, it can be seen that the methoxy derivatives **6c** and **8c** were bound mostly to the C-terminal part, compound **5c** was bound to the HHQK domain and the hydrophobic core, while compound **7c** had the least interactions with the HHQK domain (Figure 5b). These observations qualitatively correlate with the experimental results. 

#### 2.2.7. Antioxidant (AO) Activity

To evaluate the primary AO potency of the conjugates, two techniques were used. One method was the FRAP test, which exclusively evaluates the single electron transfer (SET) mechanism. The other procedure was the ABTS assay, which can detect AO activity that occurs by HAT (hydrogen atom transfer) and/or SET mechanisms. For both methods, Trolox was used as a reference AO, and ascorbic acid was used as a positive control. The data are presented in Table 3.

In the ABTS assay, the dark-green ABTS cation-radical (ABTS^•+^) is quenched by interaction with AOs. The consequent decrease in absorbance is followed spectrophotometrically [90] per the previously described method [91]. Results are calculated as Trolox Equivalent AO capacity (TEAC) units and IC_50_ values (the concentration of test compound that produces a 50% decrease in the concentration of ABTS^•+^.

The Ferric Reducing Antioxidant Power (FRAP) test involves reduction of the ferric 2,4,6-tripyridyl-s-triazine complex [Fe(TPTZ)_2_]^3+^ to an intense-blue ferrous complex [Fe(TPTZ)_2_]^2+^ [92], as previously reported [53]. Results are given as Trolox Equivalents (TE).

The results (Table 3) showed that all tested conjugates had little or no AO activity in the FRAP test. At the same time, the results of the ABTS assay demonstrated the dependence of radical-scavenging activity on the structure of the conjugates, especially on the structure of the second pharmacophore, the salicylic acid derivative.

As can be seen (Table 3), the conjugates of tacrine and salicylamide **5a–c** exhibited rather high ABTS^•+^ scavenging activity (TEAC = 0.72–0.90), near that of the level of the standard AO Trolox (TEAC = 1.00). Changes in the spacer length did not significantly affect the radical-scavenging activity. Compound **5b**, with a spacer length n=6, was the most active ABTS-radical scavenger (TEAC = 0.93), having an activity close to that of Trolox. The reference compound, salicylamide, also had an ability to scavenge free radicals, but to a lesser extent (TEAC = 0.39) than conjugates of tacrine and salicylamide. In contrast, the parent compound, salicylic acid, had no antiradical activity at the studied concentration range (1–100 μM), which was in good agreement with literature data [57].

Salicylimines **10a–c** were also quite active in the ABTS test: their antiradical activity (TEAC = 0.42–0.47) was only 1.5 times lower than that of salicylamide derivatives **5a–c**. On the contrary, difluorosalicylamides **7a–c**, showed very weak activity, 12–30 times lower than salicylamides **5a–c**. Methoxybenzoylamides **6a–c** and difluoromethoxybenzoylamides **8a–c** had no detectable ABTS^•+^-scavenging ability.

#### 2.2.8. Quantum Chemical Analyses of AO Activity

To explain the obtained structure–activity relationships for the investigated conjugates, DFT calculations were performed for four compounds with different AO activities; namely, salicylamide **5a**, methoxybenzoylamide **6a**, difluorosalicylamide **7a**, and salicylimine **10a**.

The studied compounds can be protonated under working pH values (about 4.5). According to pK_a_ values estimated by the Calculator Plugins of Marvin 21.14.0, Chem-Axon (http://www.chemaxon.com, accessed on 5 December 2022), and free MolGpka [93], three compounds were predicted to be protonated at nitrogen in the tacrine fragment: **5a**, **6a**, and **7a**. Compound **10a** was twice protonated: at the nitrogen in the tacrine fragment and at the imine nitrogen in the spacer (see Table S1 in Supplementary Materials). AO characteristics were calculated for these forms of the compounds.

In the ABTS assay, the pre-generated ABTS radical cation ABTS**^•+^** is quenched to the reduced form. ABTS**^•+^** reduction is a complex reaction that can proceed by different pathways depending on the AO and experimental conditions [94]. However, as a result, ABTS**^•+^** acquires an electron, while the AO loses either an electron or an H-atom. These reactions can be generally described by the following two equations:

ABTS**^•+^** + AH → ABTS + AH**^•+^** (1)

ABTS**^•+^** + AH → ABTS + A**^•^** + H**^+^** (2)

where AH stands for the AO molecule. In reaction (1), an electron is directly transferred from the AO to the ABTS radical. The reaction is characterized by the ionization potential (IP). In reaction (2), the electron is transferred to the ABTS radical, and the proton goes into the solvent. The reaction is characterized by the bond dissociation energy (enthalpy) (BDE), as shown in general terms in Equation (3) below.

The AO activity characteristics were calculated as is usually done [95], through the enthalpies of the corresponding optimized structures, as summarized in the following three equations:

BDE = *H*(A**^•^**) + *H*(H**^•^**) − *H*(AH) (3)

IP = *H*(AH**^•^**^+^) + *H*(e^−^) − *H*(AH) (4)

PA = *H*(A**^−^**) + *H*(H**^+^**) − *H*(AH) (5)

The terms in Equations (3)–(5) not already defined above are as follows (from left to right in each equation): *H*(A**^•^**) is the enthalpy of the AO radical after hydrogen atom abstraction; *H*(H**^•^**) is the H-atom enthalpy; *H*(AH) is the enthalpy of the parent AO molecule; *H*(AH^•+^) is the enthalpy of the AO radical cation after electron abstraction; *H*(e^−^) is the electron enthalpy; *H*(AH) is the enthalpy of the parent AO molecule; PA is proton affinity; *H*(A**^•^**) is the enthalpy of the AO radical after hydrogen atom abstraction; *H*(H**^+^**) is the proton enthalpy; and *H*(AH) is the enthalpy of the parent AO molecule.

The enthalpies of the solvated electron, proton, and H**^•^** radical were taken from the literature [96].

Table 4 presents a comparison of calculated BDE and IP values in ethanol solvent with experimental AO activity as TEAC and IC_50_ values. For each compound, the smallest BDE is provided. For **5a**, **7a**, and **10a**, the BDE values for the H-atom of the OH bond in the aromatic fragment are given. For compound **6a**, the H-atom of the OH bond in the aromatic moiety was replaced by a methyl group, and the H-atom of the NH bond in the tacrine fragment had the lowest BDE in the molecule, which was, however, higher compared to the BDE for the H-atom of the OH bond in the aromatic moiety for the other compounds.

Table 4 shows that the calculated BDE values for compounds **5a**, **10a**, and **6a** were inversely related to the experimental TEAC values, while there was no such relationship between IP and TEAC values. These results indicate that ABTS radical scavenging proceeds through H-atom abstraction from the AO molecule, while the electron is transferred to the ABTS radical cation, and the proton passes into the solvent. This is consistent with the fact that little or no AO activity of the studied compounds was observed in the FRAP assay (Table 3).

Among the four compounds listed in Table 4, the difluorosalicylamide **7a** was an outlier with respect to the inverse relationship between BDE and TEAC shown by the other three conjugates; hence, the data for **7a** are shaded gray to accentuate this finding. Thus, with a low TEAC value of 0.06, if compound **7a** had fit within the trend exhibited by the other compounds, we would have expected its BDE value to have been close to that of compound **6a**; instead, its BDE was 82.0 kcal/mol, close to that of compound **5a** (81.7 kcal/mol). It is noteworthy that the replacement of two hydrogen atoms in the *para*- and *meta*-positions of compound **5a** with fluorine atoms in compound **7a** had almost no effect on the BDE value. Moreover, the PA, as expected, decreased: the PA of compounds **5a** and **7a** were 31.3 and 27.8 kcal/mol, respectively.

A strong decrease in the experimental AO activity of compound **7a** compared to compound **5a** can be explained by the formation of a pseudo hydrogen-bond network due to C–H···F–C hydrogen bonds. Recently, it has been shown that C–H···F–C hydrogen bonds play a significant role in altering the packing characteristics of molecules in building specific crystal lattices even in the presence of strong hydrogen bonds [97,98]. Therefore, despite the weakness of C–H···F–C hydrogen bonds, we hypothesized that such a network would hinder access to reactive hydrogen, which would lead to a decrease in AO activity.

#### 2.2.9. MD Simulations with QM/MM Potentials

To assess our pseudo hydrogen-bond network hypothesis, we performed MD simulations with QM/MM potentials for the pairs of model dimers of difluorosalicylamide fragment **7a** and its non-fluorinated analog **5a**. Two starting configurations of the dimers were chosen: one with C–H···F–C hydrogen bonds and the other with π-π stacking interactions.

**Figure 6:**
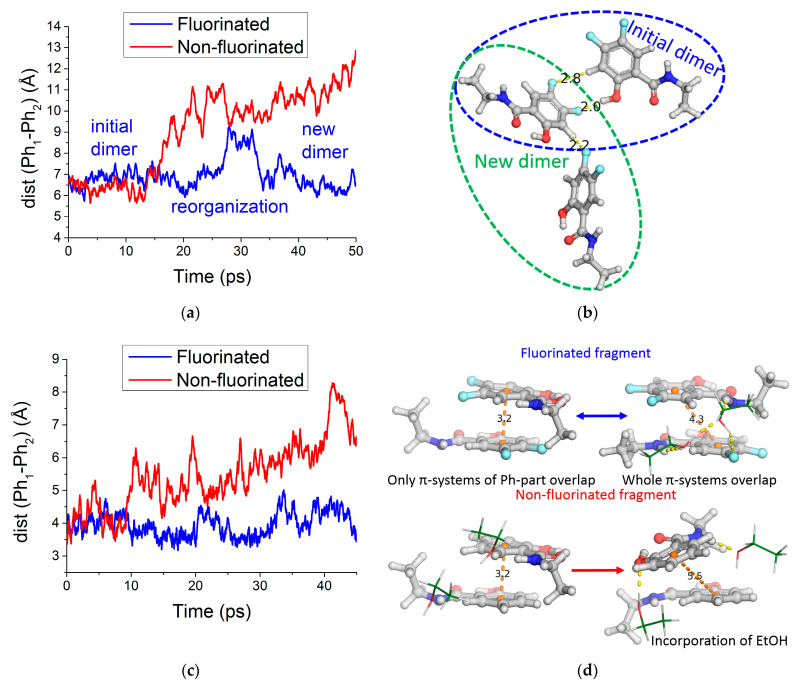
Results of MD simulations with QM/MM potentials of a sample dimer of two difluorosalicylamide fragments in comparison with their non-fluorinated analog in explicit ethanol/water solvent. (**a**) Evolution of the distance between centers of the phenolic rings of the two molecules (Ph_1_–Ph_2_ distance) over the QM/MM-MD trajectory for the fluorinated dimer with initial C–H···F–C hydrogen bonds (blue line) and its non-fluorinated analog (red line); (**b**) visualization of the rearrangement of the intermolecular C–H···F–C hydrogen bonds of the fluorinated dimer (water and ethanol molecules are not shown). (**c**) Evolution of the distance between centers of the phenolic rings for initial fragments with π–π stacking for fluorinated (blue line) and non-fluorinated (red line) systems. Fluctuation of distance between centers of the phenolic rings of the fluorinated dimer reflects coexistence of the two variants of π–π stacking; (**d**) visualization of the changes in the two systems (some of the ethanol molecules are shown).

In the case of the dimer with C–H···F–C hydrogen bonds, a partial dissociation of the dimer was observed. During this process, the C–H···F–C hydrogen bonds between the two molecules rearranged, and a new dimer was formed. For the non-fluorinated analog, dissociation of the dimer was irreversible at the same timescale (Figure 6a,b).

In the case of π–π stacking interactions for the fluorinated fragments, the dimer was very stable. Along the trajectory, π–π stacking involved either π-systems of only the phenyl rings in the salicyl moiety, or the entire π-system, including the amide fragments (both variants of π stacking are shown in Figure 6d). At the same simulation timescale, for the non-fluorinated analog, the incorporation of ethanol molecules was observed, impairing the π–π stacking (Figure 6c,d).

Thus, these two examples from MD simulations with QM/MM potentials provided evidence that the fluorination of salicylamide fragments favors aggregation, either through the C–H···F–C hydrogen bonds, or through the stabilization of π–π stacking. Therefore, the formation of the pseudo hydrogen-bond networks by compound **7a** can hinder access of ABTS**^•+^** to reactive hydrogen, resulting in a decrease in its AO activity. In contrast, the AO activity of the other studied compounds was determined by their BDE values.

#### 2.2.10. Metal-Chelating Properties

**Figure 7:**
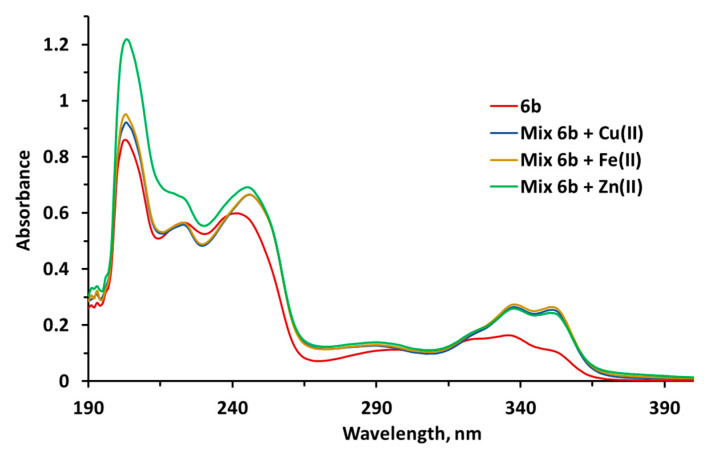
UV spectra of compound **6b** and mixtures of **6b** with Cu^2+^, Fe^2+^, and Zn^2+^ ions.

**Figure 8:**
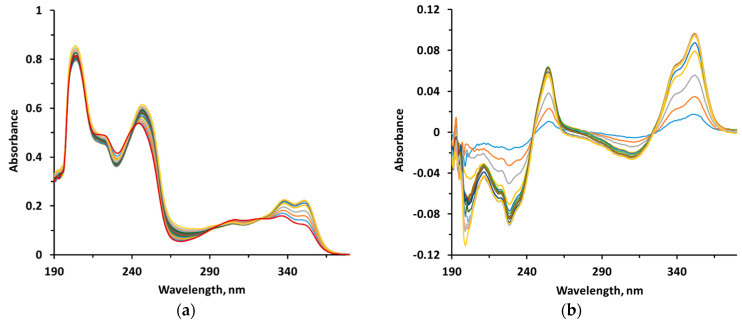
(**a**) UV absorption spectra of **5b** (20 µM) in ethanol after addition of increasing concentrations of CuCl_2_ (2–34 µM). (**b**) the differential spectra due to the **5b**–Cu^2+^ complex formation obtained by numerical subtraction from the spectra of the mixture of the spectra of the Cu^2+^ alone and **5b** alone at the corresponding concentrations.

Previous work by other groups [38,99,100] and our laboratories [59] have employed spectroscopic techniques to assess metal complexation by various compounds. In the present investigation, we used the UV-Vis spectroscopy to study in detail the complexation abilities of our conjugates **5b**, **6b**, **7b**, **8b**, and **10b** for biometals such as Cu^2+^, Fe^2+^, and Zn^2+^ in ethanol solutions. The results are shown in Figure 7, Figure 8 and Figures S53–S58, and Table 5.

Electronic spectra of **5b** [59], **6b** (Figure 7 and Figure S53), **7b**, **8b**, and **10b** (Figures S54–S56) were found to be similar and to have two broad bands. The broad band from 270 to 400 nm was ascribed to π–π* transitions, and the peaks around 240 nm were attributed to the intramolecular charge transfer. All the spectra of **5b**, **6b**, **7b**, **8b**, and **10b** exhibited a red shift (about 10 nm) after the addition of biometals Fe^2+^, Zn^2+^, and Cu^2+^ (Table 5).

In Figure 8a, the UV-Vis spectra of conjugate **5b** at increasing Cu^2+^ concentrations are shown as an example. The increase in absorbance, which could be better assessed by an analysis of the differential spectra (Figure 8b), indicates that there is an interaction between Cu^2+^ and **5b**. A similar behavior was also observed between Cu^2+^ and compounds **6b** and **10b** (Figures S57 and S58). 

**Figure 9:**
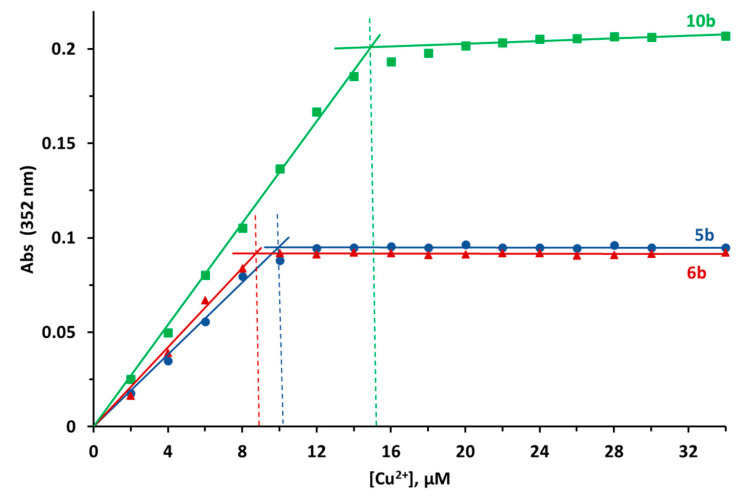
Absorbance of the **5b**–Cu^2+^, **6b**–Cu^2+^, and **10b**–Cu^2+^ complexes at 352 nm as a function of the concentration of Cu^2+^ (compounds concentration is 20 µM). Vertical dashed lines mark the metal concentration at the breakpoints and indicate ligand–metal molar ratios of 2:1 for **5b**, 2.3:1 for **6b**, and 1.3:1 for **10b**.

The ratio of ligand/metal ion in the Cu^2+^ complexes was investigated for conjugates **5b**, **6b** and **10b** by mixing a fixed concentration of the tested compound (20 µM) with increasing concentrations of metal ion (2–34 µM) [99] (Figure 8a,b, Figures S57 and S58). The titration curves (Figure 9) suggested a 2:1 molar ratio of **5b** to Cu^2+^ in the complex. The complex **6b**–Cu^2+^ demonstrated an increase in the molar ratio of ligand to metal (2.3:1) that could be due to the presence of the methoxy substituent. The major difference in the titration curves was observed for **10b** with Cu^2+^, which suggested the formation of a nearly equimolar ligand–metal complex (1.3:1).

These results indicate that the studied conjugates of tacrine and salicylic acid can effectively chelate Cu^2+^, Fe^2+^, and Zn^2+^ ions, and could, therefore, serve as metal chelators for the potential treatment of AD.

#### 2.2.11. Cytotoxicity of Hybrids and Tacrine: MTT Assay in Primary Mouse Hepatocytes

Tacrine is known to have been withdrawn from clinical use due to its high hepatotoxicity [101]. Accordingly, in the present work, we paid special attention to the study of the cytotoxic activity of compounds on primary cultures of mouse hepatocytes using standard MTT methodology [102]. The IC_50_ values (concentration of the compound resulting in a 50% decrease in cell viability) for compounds **5b**, **6c**, **7b**, **8b**, and **10c** are shown in Table 6.

The tested conjugates exhibited cytotoxicity that was comparable (**6c** and **8b**) or lower (**5b**, **7b**, and **10c**) than that of tacrine on mouse hepatocytes. In particular, the difluorosalicylamide **7b** was ~4.0 times less toxic than tacrine, based on the midpoints of their respective IC_50_ curves.

#### 2.2.12. Prediction of ADMET, Physicochemical, and PAINS Profiles

Table 7 lists a selection of computed ADMET and physicochemical properties for compounds **5–8** and **10**. Predicted values for intestinal absorption were high, suggesting favorable absorption following oral administration. Values for blood-brain barrier permeability were moderate to high (levels attained in brain are ~0.75 to four times the plasma levels), indicating the likelihood that the compounds would be capable of exerting activity in the CNS. Estimators of cardiac toxicity potential (hERG p*K_i_* and pIC_50_) were 4.8 to 7.0, which occupied the low to middle segment of the potential range (3.0 to 9.0). Molecular weights and predicted values for lipophilicity and aqueous solubility were within or close to generally recognized guidelines for drug likeness. Overall, values for the integral quantitative estimate of drug likeness were 0.2 to 0.5, and some compounds violated traditional limits of Lipinski’s rule of five. However, the model applicability domain did not encompass some of the compounds; hence, predicted values for these agents should be interpreted with caution. Finally, no structural alerts were flagged by the pan assay interference compounds (PAINS) filter. Taken together, considering prospective lead compounds in the first discovery phase, the predicted ADMET, physicochemical, and PAINS profiles were acceptable. Even so, it is always prudent to undertake further research to optimize structures for enhanced efficacy and safety.

## 3. Experimental Section

### 3.1. Chemistry

Ethanol, chloroform, methylene chloride, salicylic acid, ammonium hydroxide, potassium iodide, sodium hydroxide, and sodium sulfate were obtained from VEKTON AO (St. Petersburg, Russia). Diisopropylethylamine, pentanol-1, 1,4-diaminobutane, 1,6-diaminohexane, 1,8-diaminooctane, and salicylaldehyde were purchased from Alfa Aesar via Thermo Fisher Scientific (Kandel, Germany). The deuterated solvents DMSO-*d*_6_ and CDCl_3_ were acquired from SOLVEX LLC (Skolkovo Innovation Center, Moscow, Russia). All solvents, chemicals, and reagents were used without purification. Melting points were determined in open capillaries on a Stuart SMP30 melting point apparatus (Bibby Scientific Limited, Staffordshire, UK) and were uncorrected. The IR spectra were recorded on a Perkin Elmer Spectrum Two instrument (PerkinElmer, Waltham, MA, USA) using a frustrated total internal reflection accessory with a diamond crystal. The ^1^H and ^19^F NMR spectra were registered on a Bruker DRX-400 spectrometer (400 or 376 MHz, respectively) or a Bruker Avance^III^ 500 spectrometer (500 or 470 MHz, respectively) (Bruker, Karlsruhe, Germany). The ^13^C NMR spectra were recorded on a Bruker Avance^III^ 500 spectrometer (125 MHz). The internal standard was SiMe_4_ (for ^1^H and ^13^C NMR spectra) and C_6_F_6_ (for ^19^F NMR spectra, δ–162.9 ppm). The microanalyses (C, H, N) were carried out on a PerkinElmer PE 2400 series II elemental analyzer (PerkinElmer, Waltham, MA, USA). The high-resolution mass spectrometry (HRMS) was performed using a Bruker Daltonik MaXis Impact HD (Bruker, Karlsruhe, Germany) quadrupole time-of-flight mass spectrometer with positive electrospray ionization from methanol solutions, flow rate 180 μL·h^−1^ with parameters optimized for small molecules detection based on a pre-installed method for infusion analysis. The column chromatography was performed on silica gel 60 (0.062–0.2 mm) (Macherey-Nagel GmbH & Co KG, Duren, Germany).

2-Methoxybenzoic acid **4b [103]**, 4,5-difluoro-2-hydroxybenzoic acid **4c**, 4,5-difluoro-2-methoxybenzoic acid **4d** [104], and aminomethylene-tacrines **3a–c** [55] were synthesized by referring to the previously published methods.

#### 3.1.1. Synthesis of Compounds **3a–c** (General Procedure)

A mixture of 9-chloro-1,2,3,4-tetrahydroacridine **1** (1 g, 46 mmol), diaminoalkane **2a–c** (230 mmol), and a catalytic amount of potassium iodide in pentanol-1 (15 mL) was placed in screw-cup vials and heated at 160 °C for 16 h; then the reaction mass was concentrated under reduced pressure. The residue was diluted with chloroform (50 mL), the organic layer was washed with a 10% (*w*/*v*) solution of sodium hydroxide (3 × 50 mL) and water (3 × 50 mL), dried over sodium sulfate, and evaporated. The residues were purified by silica gel column chromatography (eluent: CHCl_3_/EtOH/NH_4_OH 50:1:0.1 followed by CHCl_3_/EtOH/NH_4_OH 1:5:0.1). The physicochemical properties of compounds **3a–c** coincided with literature data [105,106,107,108].

#### 3.1.2. General Procedure for the Synthesis of Compounds **5**–**8**

Diisopropylethylamine (0.43 mL, 2.5 mmol) was added to a solution of compound **3a–c** (1.1 mmol) in anhydrous DCM (10 mL). The reaction mixture was stirred for 20 min, and then HATU (0.418 g, 1.1 mmol) was added. After stirring the reaction mixture for 30 min, the solution of compound **4a–d** (1.0 mmol) in anhydrous DCM (10 mL) was added, and the reaction mixture was stirred for 4 h at room temperature. Next, the reaction mixture was concentrated on a rotary evaporator. The residue was dissolved in chloroform (20 mL), the organic layer was washed with a saturated solution of NaHCO_3_ (2 × 20 mL) and water (2 × 20 mL), dried over sodium sulfate, and evaporated. The residue was purified by silica gel column chromatography (eluent CHCl_3_/EtOH/NH_4_OH 25:1:0.1 followed by CHCl_3_/EtOH/NH_4_OH 5:1:0.1). The physicochemical properties of the compounds **5a–c** coincided with literature data [59].

*2-Methyl-N-[4-(1,2,3,4-tetrahydroacridin-9-ylamino)butyl]benzamide* (**6a**): yellow oil; yield 0.286 g (71%). IR ν_max_ 3393, 3072, 2934, 2862 (NH, OH, CH), 1640 (C=O), 1533, 1483, 1464, 1436 (NH, C=C, C=N) cm^−1^. ^1^H NMR (CDCl_3_, 500 MHz) δ 1.69–1.79, 1.84–1.95 (8H, both m, 4CH_2_), 2.67–2.74, 3.00–3.09 (4H, both m, 2CH_2_), 3.48–3.56 (4H, m, CH_2_), 3.88 (3H, s, CH_3_), 4.02 (1H, br. s, NH), 6.95 (1H, d, *J* 8.3 Hz, H_Ar_), 7.08 (1H, t, *J* 7.5 Hz, H_Ar_), 7.32 (1H, td, *J* 7.7, 0.7 Hz, H_Ar_), 7.44 (1H, td, *J* 7.6, 1.8 Hz, H_Ar_), 7.53 (1H, td, *J* 7.7, 0.8 Hz, H_Ar_), 7.89 (1H, d, *J* 8.3 Hz, H_Ar_), 7.90 (1H, br. s, NH), 7.95 (1H, d, *J* 8.4 Hz, H_Ar_), 8.20 (1H, dd, *J* 7.8, 1.8 Hz, H_Ar_). ^13^C NMR (CDCl_3_, 125 MHz) δ 22.73, 22.98, 24.83, 27.17, 29.11, 34.03, 39.28, 48.95, 55.83, 111.23, 116.30, 120.33, 121.30, 121.41, 122.63, 123.66, 128.19, 128.75, 132.20, 132.69, 147.43, 150.44, 157.32, 158.54, 165.33. Calcd for C_25_H_29_N_3_O_2_: C 74.41, H 7.24, N 10.41. Found: C 74.17, H 7.36, N 10.73. HRMS *m/z* 404.2332 (calcd for C_25_H_30_N_3_O_2_ 404.2333).

*2-Methyl-N-[4-(1,2,3,4-tetrahydroacridin-9-ylamino)hexyl]benzamide* (**6b**): yellow oil; yield 0.327 g (76%). IR ν_max_ 3392, 3072, 2929, 2857 (NH, OH, CH), 1640 (C=O), 1532, 1483, 1463, 1436 (NH, C=C, C=N) cm^−1^. ^1^H NMR (CDCl_3_, 500 MHz) δ 1.40–1.48 (4H, m, 2CH_2_), 1.59–1.60, 1.65–1.71 (4H, both m, 2CH_2_), 1.89–1.93 (4H, m, CH_2_), 2.67–2.74, 3.03–3.08 (4H, both m, 2CH_2_), 3.43–3.51 (4H, m, 2CH_2_), 3.92 (3H, s, CH_3_), 4.02 (1H, br. s, NH), 6.95 (1H, d, *J* 8.2 Hz, H_Ar_), 7.07 (1H, td, *J* 7.5, 0.7 Hz, H_Ar_), 7.33 (1H, td, *J* 7.6, 0.9 Hz, H_Ar_), 7.43 (1H, td, *J* 8.4, 1.8 Hz, H_Ar_), 7.50 (1H, td, *J* 7.6, 1.0 Hz, H_Ar_), 7.86 (1H, br. s, NH), 7.90 (1H, d, *J* 8.4 Hz, H_Ar_), 7.96 (1H, d, *J* 8.2 Hz, H_Ar_), 8.20 (1H, dd, *J* 7.8, 1.8 Hz, H_Ar_). ^13^C NMR (CDCl_3_, 125 MHz) δ 22.28, 22.55, 24.32, 26.12, 26.29, 29.08, 31.18, 33.51, 39.05, 48.88, 55.43, 110.81, 115.42, 119.73, 120.86, 121.16, 122.35, 123.12, 127.80, 128.18, 131.77, 132.14, 146.93, 150.27, 156.88, 157.94, 164.78. Calcd. for C_27_H_33_N_3_O_2_: C 75.14, H 7.71, N 9.74. Found: C 75.32, H 7.94, N 9.89. HRMS *m/z* 432.2639 (calcd for C_27_H_34_N_3_O_2_ 432.2646).

*2-Methyl-N-[4-(1,2,3,4-tetrahydroacridin-9-ylamino)octyl]benzamide* (**6c**): yellow oil, yield 0.286 g (71%). IR ν_max_ 3393, 3072, 2930, 2858 (NH, OH, CH), 1640 (C=O), 1533, 1483, 1464, 1464 (NH, C=C, C=N) cm^−1^. ^1^H NMR (CDCl_3_, 500 MHz) δ 1.31–1.43 (8H, m, 4CH_2_), 1.55–1.63, 1.63–1.69 (4H, m, 2CH_2_), 1.87–1.96 (4H, m, 2CH_2_), 2.67–2.74, 3.02–3.09 (4H, m, 2CH_2_), 3.42–3.51 (4H, m, 2CH_2_), 3.94 (3H, s, CH_3_), 3.96 (1H, br.s, NH), 6.95 (1H, d, *J* 8.3 Hz, H_Ar_), 7.07 (1H, td, *J* 7.5, 0.6 Hz, H_Ar_), 7.34 (1H, td, *J* 7.6, 1.0 Hz, H_Ar_), 7.43 (1H, td, *J* 8.5, 1.8 Hz, H_Ar_), 7.54 (1H, td, *J* 7.7, 1.1 Hz, H_Ar_), 7.85 (1H, br. s, NH), 7.90 (1H, d, *J* 8.4 Hz, H_Ar_), 7.95 (1H, d, *J* 8.3 Hz, H_Ar_), 8.21 (1H, dd, *J* 7.8, 1.8 Hz, H_Ar_). ^13^C NMR (CDCl_3_, 125 MHz) δ 22.77, 23.03, 24.76, 26.83, 26.91, 29.12, 29.25, 29.52, 31.70, 34.04, 39.64, 49.47, 55.88, 111.24, 115.85, 120.22, 121.30, 121.70, 122.82, 123.52, 128.19, 128.71, 132.22, 132.53, 147.48, 150.72, 157.33, 158.43, 165.16. Calcd. for C_29_H_37_N_3_O_2_: C 75.78, H 8.11, N 9.14. Found: C 76.07, H 8.40, N 9.32. HRMS *m/z* 460.2961 (calcd for C_29_H_38_N_3_O_2_ 460.2959).

*4,5-Difluoro-2-hydroxy-N-[4-(1,2,3,4-tetrahydroacridin-9-ylamino)butyl]benzamide* (**7a**): white powder, yield 0.191 g (45%), mp 123–124 °C. IR ν_max_ 3405, 3346, 2925, 2855 (NH, OH, CH), 1634 (C=O), 1435, 1562, 1364 (NH, C=C, C=N), 1180 (CF) cm^−1^. ^1^H NMR (DMSO-*d*_6_, 500 MHz) δ 1.51–1.60, 1.60–1.67 (4H, both m, 2CH_2_), 1.74–1.84 (4H, m, 2CH_2_), 2.66–2.72, 2.86–2.92, 3.23–3.31, 3.46–3.54 (8H, all m, 4CH_2_), 5.80 (1H, br. s, NH), 6.86 (1H, dd, *J* 12.5, 7.0 Hz, H_Ar_), 7.35 (1H, td, *J* 7.6, 1.0 Hz, H_Ar_), 7.55 (1H, td, *J* 7.7, 0.8 Hz, H_Ar_), 7.70 (1H, d, *J* 8.0 Hz, H_Ar_), 7.83 (1H, dd, *J* 12.0, 9.6 Hz, H_Ar_), 8.15 (1H, d, *J* 8.4 Hz, H_Ar_), 9.20 (1H, br. s, NH), OH is not observed due to deuterium exchange. ^13^C NMR (DMSO-*d*_6_, 125 MHz) δ 22.03, 22.48, 24.85, 26.18, 27.94, 32.56, 47.46, 79.15, 106.01 (d, *J* 18.1 Hz), 111.88 (unsolv. dd), 115.13, 115.70 (unsolv. dd), 119.47, 123.31, 123.45, 126.75, 128.56, 141.59 (dd, *J* 234.7, 13.3 Hz), 145.39, 151.08, 152.10 (dd, *J* 250.6, 13.6 Hz), 156.66, 158.78 (d, *J* 11.2 Hz), 167.08. ^19^F NMR (CDCl_3_, 470 MHz,) δ 10.70–11.90 (1F, m, F_Ar_); 31.35–31.85 (1F, m, F_Ar_). Calcd. for C_24_H_25_F_2_N_3_O_2_: C 67.75, H 5.92, N 9.88. Found: C 67.98, H 5.79, N 9.69. HRMS *m/z* 426.1990 (calcd for C_24_H_26_F_2_N_3_O_2_ 426.1988).

*4,5-Difluoro-2-hydroxy-N-[4-(1,2,3,4-tetrahydroacridin-9-ylamino)hexyl]benzamide* (**7b**): white powder, yield 0.163 g (36%), mp 125–127 °C. IR ν_max_ 3278, 3145, 3061, 2930, 2853 (NH, OH, CH), 1638 (C=O), 1569, 1529, 1474, 1436 (NH, C=C, C=N), 1192 (CF) cm^−1^. ^1^H NMR (DMSO-*d*_6_, 500 MHz) δ 1.25–1.34 (4H, m, 2CH_2_), 1.44–1.52, 1.53–1.61 (4H, both m, 2CH_2_), 1.73–1.85 (4H, m, 2CH_2_), 2.64–2.71, 2.87–2.93, 3.21–3.28, 3.42–3.49 (8H, all m, 4CH_2_), 5.76–5.87 (1H, m, NH), 6.81 (1H, dd, *J* 12.6, 7.0 Hz, H_Ar_), 7.35 (1H, td, *J* 7.7, 1.1 Hz, H_Ar_), 7.56 (1H, td, *J* 7.7, 1.0 Hz, H_Ar_), 7.71 (1H, d, *J* 8.3 Hz, H_Ar_), 7.85 (1H, dd, *J* 12.0, 9.7 Hz, H_Ar_), 8.14 (1H, d, *J* 8.4 Hz, H_Ar_), 9.30 (1H, br. s, NH), OH is not observed due to deuterium exchange. ^13^C NMR (DMSO-*d*_6_, 125 MHz) δ 22.01, 22.46, 24.83, 25.93, 26.14, 28.77, 30.35, 32.44, 47.73, 79.16, 105.93 (d, *J* 17.8 Hz), 112.45 (unsolv. dd, *J* 2.8 Hz), 114.91, 115.94 (dd, *J* 18.9, 1.8 Hz), 119.34, 123.37, 123.45, 126.53, 128.68, 141.44 (dd, *J* 234.3, 13.0 Hz), 145.19, 151.99 (dd, *J* 250.7, 14.1 Hz), 151.28, 156.47, 159.02 (d, *J* 11.4 Hz), 166.70. ^19^F NMR (DMSO-*d*_6_, 470 MHz) δ 10.64–11.20 (1F, m, F_Ar_), 31.08–31.72 (1F, m, F_Ar_). Calcd. for C_26_H_29_F_2_N_3_O_2_: C 68.86, H 6.45, N 9.27. Found: C 68.05, H 6.42, N 8.93. HRMS *m/z* 454.2296 (calcd for C_26_H_30_F_2_N_3_O_2_ 454.2301).

*4,5-Difluoro-2-hydroxy-N-[4-(1,2,3,4-tetrahydroacridin-9-ylamino)octyl]benzamide* (**7c**): white powder, yield 0.245 g (51%), mp 127–130 °C. IR ν_max_ 3319, 3078, 2925, 2853 (NH, OH, CH), 1643 (C=O), 1566, 1520, 1475, 1437 (NH, C=C, C=N), 1182 (CF) cm^−1^. ^1^H NMR (DMSO-*d*_6_, 500 MHz) δ 1.18–1.31 (8H, m, CH_2_), 1.43–1.51, 1.51–1.60 (4H, both m, 2CH_2_), 1.75–1.85 (4H, m, CH_2_), 2.65–2.72, 2.87–2.94, 3.20–3.29, 3.41–3.49 (8H, all m, 4CH_2_), 5.80 (1H, br. s, NH), 6.85 (1H, dd, *J* 12.5, 7.0 Hz, H_Ar_), 7.36 (1H, td, *J* 7.6, 1.0 Hz, H_Ar_), 7.56 (1H, td, *J* 7.7, 0.8, H_Ar_), 7.71 (1H, d, *J* 8.3 Hz, H_Ar_), 7.87 (1H, dd, *J* 12.0, 9.7 Hz, H_Ar_), 8.14 (1H, d, *J* 8.4 Hz, H_Ar_), 9.22 (1H, br. s, NH), OH is not observed due to deuterium exchange. ^13^C NMR (DMSO-*d*_6_, 125 MHz) δ 22.03, 22.45, 24.78, 26.09, 26.23, 28.50 (2C), 28.68, 30.34, 32.54, 47.75, 79.10, 105.93 (d, *J* = 18.1 Hz), 112.11 (unsolv. dd), 115.00, 115.79 (dd, *J* = 19.1, 2.1 Hz), 119.43, 123.27, 123.35, 126.73, 128.50, 141.57 (dd, *J* 234.8, 13.2 Hz), 145.39, 152.01 (dd, *J* 250.9, 14.1 Hz), 151.15, 156.63, 158.72 (d, *J* 11.0 Hz), 166.84. ^19^F NMR (470 MHz, DMSO-*d*_6_) δ 11.48–12.41 (1F, m, F_Ar_), 31.85–32.97 (1F, m, F_Ar_). Calcd. for C_28_H_33_F_2_N_3_O_2_: C 69.83, H 6.91, N 8.73. Found: C 69.60, H 6.68, N 8.59. HRMS *m/z* 482.2609 (calcd for C_28_H_34_F_2_N_3_O_2_ 482.2614).

*4,5-Difluoro-2-methoxy-N-[4-(1,2,3,4-tetrahydroacridin-9-ylamino)butyl]benzamide* (**8a**): yellow oil, yield 0.237 g (54%). IR ν_max_ 3401, 3063, 2933, 2863 (NH, CH), 1649 (C=O), 1614, 1504, 1411, 1317 (NH, C=C, N–H, C=N), 1196 (CF) cm^−1^. ^1^H NMR (500 MHz, CDCl_3_) δ 1.69–1.74, 1.89–1.92 (8H, both m, 4CH_2_), 2.68–2.75, 3.02–3.08, 3.45–3.49, 3.49–3.55 (8H, all m, 4CH_2_), 3.85 (3H, s, CH_3_), 4.02 (1H, br. s, NH), 6.77 (1H, dd, *J* 11.6, 6.1 Hz, H_Ar_), 7.33 (1H, t, *J* 7.6 Hz, H_Ar_), 7.54 (1H, t, *J* 7.6 Hz, H_Ar_), 7.72–7.81 (1H, m, NH), 7.90 (1H, d, *J* 8.4 Hz, H_Ar_), 7.94 (1H, d, *J* 8.5 Hz, H_Ar_), 8.02–8.09 (1H, m, H_Ar_). ^13^C NMR (CDCl_3_, 125 MHz) δ 22.72, 22.99, 24.84, 27.09, 29.08, 33.99, 39.46, 48.88, 56.73, 101.39 (d, *J* 21.7 Hz), 116.33, 117.93 (unsolv. dd, *J* 3.7 Hz), 120.59, 120.67 (dd, *J* 20.3, 1.9 Hz), 122.58, 123.7, 128.25, 128.74, 144.72 (dd, *J* 242.4, 12.6 Hz), 147.37, 150.41, 152.15 (dd, *J* 254.4, 14.3 Hz), 153.63 (dd, *J* 7.8, 1.6 Hz), 158.53, 163.33. ^19^F NMR (CDCl_3_, 470 MHz): δ 15.45 (1F, ddd, *J* 22.5, 11.4, 6.1 Hz, F_Ar_), 32.47 (1F, ddd, *J* 22.5, 11.4, 6.1 Hz, F_Ar_). Calcd. for C_25_H_27_F_2_N_3_O_2_: C 68.32, H 6.19, N 9.56. Found: C, 68.55; H, 6.40; N, 9.58. HRMS *m/z* 440.2137 (calcd for C_25_H_28_F_2_N_3_O_2_ 440.2144).

*4,5-Difluoro-2-methoxy-N-[4-(1,2,3,4-tetrahydroacridin-9-ylamino)hexyl]benzamide* (**8b**): yellow oil, yield 0.275 g (59%). IR ν_max_ 3404, 3063, 2932, 2858 (NH, CH), 1650 (C=O), 1614, 1504, 1411, 1317 (NH, C=C, N–H, C=N), 1198 (CF) cm^−1^. ^1^H NMR (CDCl_3_, 500 MHz) δ 1.37–1.51 (4H, m, 2CH_2_), 1.56–1.64, 1.64–1.74 (4H, both m, 2CH_2_), 1.88–1.94 (4H, m, CH_2_), 2.47–2.67 (2H, m, CH_2_), 3.01–3.08 (2H, s, CH_2_), 3.43 (2H, dd, *J* 13.0, 7.0 Hz, CH_2_), 3.48 (2H, t, *J* = 7.2 Hz, CH_2_), 3.89 (3H, s, CH_3_), 3.97 (1H, br. s, NH), 6.77 (1H, dd, *J* 11.6, 6.1 Hz, H_Ar_), 7.33 (1H, td, *J* 7.6, 0.9 Hz, H_Ar_), 7.54 (1H, td, *J* 7.7, 1.0 Hz, H_Ar_), 7.74 (1H, br. s, NH), 7.90 (1H, d, *J* 8.4 Hz, H_Ar_), 7.95 (1H, d, *J* 8.4 Hz, H_Ar_), 8.05 (1H, dd, *J* 11.4, 9.5 Hz, H_Ar_). ^13^C NMR (CDCl_3_, 125 MHz) δ 22.73, 22.99, 24.76, 26.54, 26.7, 29.44, 31.6, 33.98, 39.65, 49.30, 56.75, 101.37 (d, *J* = 21.8 Hz), 115.91, 118.12 (unsolv. dd), 120.19, 120.67 (dd, *J* 20.3, 1.9 Hz), 122.74, 123.55, 128.21, 128.68, 144.70 (dd, *J* 242.2, 12.6 Hz), 147.40, 150.64, 152.07 (dd, *J* 254.2, 14.2 Hz), 153.61 (dd, *J* 7.8, 2.0 Hz), 158.42, 163.20. ^19^F NMR (CDCl_3_, 376 MHz): δ 15.36 (1F, ddd, *J* 22.5, 11.4, 6.1 Hz, F_Ar_), 32.22 (1F, ddd, *J* 22.3, 11.5, 9.5 Hz, F_Ar_). Calcd. for C_27_H_31_F_2_N_3_O_2_: C 69.36, H 6.68, N 8.99. Found: C, 69.15; H, 6.88; N, 8.75. HRMS *m/z* 468.2457 (calcd for C_27_H_32_F_2_N_3_O_2_ 468.2457).

*4,5-Difluoro-2-methoxy-N-[4-(1,2,3,4-tetrahydroacridin-9-ylamino)octyl]benzamide* (**8c**): yellow oil, yield 0.306 g (62%). IR ν_max_ 3404, 3063, 2927, 2855 (NH, CH), 1650 (C=O), 1614, 1505, 1463, 1411, 1317 (NH, C=C, N–H, C=N), 1198 (CF) cm^−1^. ^1^H NMR (CDCl_3_, 500 MHz) δ 1.31–1.43 (8H, m, 4CH_2_), 1.55–1.62, 1.65–1.73 (4H, both m, 2CH_2_), 1.91 (4H, d, *J* 3.0 Hz, CH_2_), 2.68, 3.11 (4H, both s, 2CH_2_), 3.38–3.46 (2H, m, CH_2_), 3.53–3.62 (2 H, m, CH_2_), 3.92 (3H, s, CH_3_), 4.37 (1H, br. s, NH), 6.78 (1H, dd, *J* 11.6, 6.1 Hz, H_Ar_), 7.36 (1H, t, *J* 7.6 Hz, H_Ar_), 7.57 (1H, t, *J* 7.6 Hz, H_Ar_), 7.74 (1H, br. s, NH), 8.00 (1H, d, *J* 8.5 Hz, H_Ar_), 8.02–8.10 (2H, m, H_Ar_). ^13^C NMR (CDCl_3_, 125 MHz) δ 22.26, 22.74, 24.46, 26.75, 26.85, 29.05, 29.16, 29.44, 31.54, 32.70, 39.78, 49.24, 56.79, 101.39 (d, *J* 21.7 Hz), 114.64, 118.18 (unsolv. dd, *J* 3.8 Hz), 119.17, 120.65 (dd, *J* 20.3, 1.8 Hz), 123.09, 123.87, 126.91, 128.43, 129.13, 144.70 (dd, *J* 242.1, 12.4 Hz), 151.70, 152.05 (dd, *J* 254.1, 14.2 Hz), 153.63 (dd, *J* 7.7, 1.7 Hz), 156.85, 163.17. ^19^F NMR (CDCl_3_, 376 MHz): δ 15.35 (1F, ddd, *J* 22.4, 11.4, 6.1 Hz, F_Ar_), 31.37–32.78 (1F, m, F_Ar_). Calcd. for C_29_H_35_F_2_N_3_O_2_: C 70.28, H 7.12, N 8.48. Found: C, 70.22; H, 7.55; N, 8.20. HRMS *m/z* 496.2768 (calcd for C_29_H_36_F_2_N_3_O_2_ 496.2770).

#### 3.1.3. General Procedure of the Synthesis of Compounds **10a–c**

The salicylic aldehyde **9** (0.146 g, 1.2 mmol) was added to a solution of compound **3a–c** (1.0 mmol) in ethanol (10 mL), and the reaction mixture was stirred for 4 h. Next, the reaction mixture was concentrated on a rotary evaporator, the residue was extracted by chloroform, the organic layer was washed with water (2 × 20 mL), dried over sodium sulfate, and evaporated. The residue was purified by silica gel column chromatography (eluent CHCl_3_/EtOH 25:1 followed by CHCl_3_/EtOH 5:1).

*2-[4-(1,2,3,4-Tetrahydroacridin-9-ylamino)butyliminomethyl]phenol* (**10a**): yellow oil, yield 0.269 g (63%). IR ν_max_ 3332, 3058, 2932, 2858 (NH, OH, CH), 1630 (C=N), 1579, 1561, 1496, 1459 (NH, C=C, C=N) cm^−1^. ^1^H NMR (CDCl_3_, 400 MHz): δ 1.73–1.85, 1.85–1.95 (8H, both m, 4CH_2_), 2.63–2.74, 2.98–3.12, 3.45–3.59, 3.59–3.67 (8H, all m, 4CH_2_), 4.10 (1H, br. s, NH), 6.88 (1H, t, *J* 7.4 Hz, H_Ar_), 6.97 (1H, d, *J* 8.3 Hz, H_Ar_), 7.20–7.25 (1H, m, H_Ar_), 7.28–7.38 (2H, m, 2H_Ar_), 7.56 (1H, t, *J* 7.6 Hz, H_Ar_), 7.95 (2H, d, *J* 8.6 Hz, H_Ar_), 8.33 (1H, s, =CH), 13.43 (1H, br. s, OH). ^13^C NMR (CDCl_3_, 125 MHz) δ 22.63, 22.92, 24.74, 28.22, 29.34, 33.75, 49.07, 59.08, 115.97, 116.97, 118.59, 118.66, 120.07, 122.71, 123.78, 128.42, 128.46, 131.17, 132.24, 147.04, 150.72, 158.22, 161.09, 165.05. Calcd. for C_24_H_27_N_3_O: C 77.18, H 7.29, N 11.25. Found: C 77.44, H 7.16, N 11.40. HRMS *m/z* 374.2229 (calcd for C_24_H_28_N_3_O 374.2232).

*2-[4-(1,2,3,4-Tetrahydroacridin-9-ylamino)hexyliminomethyl]phenol* (**10b**): yellow oil, yield 0.281 g (70%). IR ν_max_ 3376, 3058, 2929, 2856 (NH, CH), 1631 (C=N), 1579, 1561, 1496, 1459, 1417 (NH, C=C, C=N) cm^−1^. ^1^H NMR (CDCl_3_, 500 MHz) δ 1.38–1.48, 1.60–1.76, 1.85–1.99 (12H, all m, 6CH_2_), 2.62–2.78, 2.98–3.12 (4H, both m, 2CH_2_), 3.47 (2H, t, *J* 7.1 Hz, CH_2_), 3.57 (2H, t, *J* 6.6 Hz, CH_2_), 3.94 (1H, br. s, NH), 6.86 (1H, d, *J* 7.5 Hz, H_Ar_), 6.95 (1H, d, *J* 8.3 Hz, H_Ar_), 7.23 (1H, dd, *J* 7.6, 1.5 Hz, H_Ar_), 7.27–7.37 (2H, m, 2H_Ar_), 7.54 (1H, td, *J* 7.7, 0.8 Hz, H_Ar_), 7.90 (1H, d, *J* 8.4 Hz, H_Ar_), 7.94 (1H, d, *J* 8.4 Hz, H_Ar_), 8.31 (1H, s, =CH), 13.62 (1H, br. s, OH). ^13^C NMR (CDCl_3_, 125 MHz) δ 22.76, 23.01, 24.77, 26.67, 26.93, 30.65, 31.66, 34.03, 49.38, 59.30, 115.94, 116.96, 118.42, 118.72, 120.23, 122.76, 123.57, 128.21, 128.73, 131.05, 132.06, 147.46, 150.65, 158.44, 161.24, 164.58. Calcd. for C_26_H_31_N_3_O: C 77.77, H 7.78, N 10.46. Found: C 77.54, H 7.77, N 10.31. HRMS *m/z* 402.2546 (calcd for C_26_H_32_N_3_O 402.2545).

*2-[4-(1,2,3,4-Tetrahydroacridin-9-ylamino)octyliminomethyl]phenol* (**10c**): yellow oil, yield 0.292 g (68%). IR ν_max_ 3383, 3059, 2927, 2854 (NH, CH), 1631 (C=N), 1580, 1561, 1496, 1460, 1417 (NH, C=C, C=N) cm^−1^. ^1^H NMR (CDCl_3_, 500 MHz) δ 1.31–1.49 (8H, m, 4CH_2_), 1.59–1.78, 1.84–2.04 (8H, both m, 4CH_2_), 2.64–2.80, 3.00–3.13 (4H, both m, 2CH_2_), 3.48 (2H, t, *J* 7.2, CH_2_), 3.53–3.65 (2H, m, CH_2_), 3.96 (1H, br. s, NH), 6.88 (1H, td, *J* 7.5, 1.0 Hz, H_Ar_), 6.95 (1H, d *J* 8.3, H_Ar_), 7.25 (1H, dd, *J* 7.6, 1.6 Hz, H_Ar_), 7.31 (1H, td, *J* 7.7, 1.6 Hz, H_Ar_), 7.35 (1H, td, *J* 7.6, 1.0 Hz, H_Ar_), 7.56 (1H, td, *J* 7.7, 1.2 Hz, H_Ar_), 7.91 (1H, d, *J* 8.3 Hz, H_Ar_), 7.97 (1H, d, *J* 8.5 Hz, H_Ar_), 8.33 (1H, s, =CH), 13.69 (1H, br. s, OH). ^13^C NMR (CDCl_3_, 125 MHz) δ 22.79, 23.04, 24.77, 26.83, 27.00, 29.17, 29.21, 30.74, 31.72, 34.08, 49.48, 59.42, 115.86, 116.98, 118.37, 118.75, 120.24, 122.81, 123.51, 128.17, 128.77, 131.01, 132.01, 147.52, 150.70, 158.45, 161.31, 164.44. Calcd. for C_28_H_35_N_3_O: C 78.28, H 8.21, N 9.78. Found: C 78.24, H 8.26, N 9.42. HRMS *m/z* 430.2856 (calcd for C_28_H_36_N_3_O 430.2858).

### 3.2. Biological Investigations

#### 3.2.1. Enzymatic Assays

##### In Vitro AChE, BChE, and CES Inhibition

All experiments were carried out in accordance with the standard protocols approved by IPAC RAS. The following items were purchased from Sigma-Aldrich (St. Louis, MO, USA): human erythrocyte AChE, equine serum BChE, porcine liver CES, acetylthiocholine iodide (ATCh), butyrylthiocholine iodide (BTCh), 5,5′-dithio-bis-(2-nitrobenzoic acid) (DTNB), 4-nitrophenyl acetate (4-NPA), tacrine, and BNPP. We measured the activity of AChE and BChE according to the colorimetric Ellman procedure (λ = 412 nm), as described in detail in [109]. CES activity was assessed as described in [109] by following the release of 4-nitrophenol spectrophotometrically (λ = 405 nm) using 4-NPA as a substrate. Freshly prepared solutions of the enzymes were used, which retained a constant activity during the experiment (2–2.5 h). Chromophore absorbances were measured with a FLUOStar Optima microplate reader (BMG Labtech, Ortenberg, Germany). DMSO (2% *v/v*) was employed as the solvent; the concentration used did not alter the activities of the enzymes (data not shown). Initially, we used a single concentration of 20 µM for all compounds. Subsequently, IC_50_ values (µM) were determined for the most active compounds against AChE, BChE, and CES.

##### Kinetic Study of AChE and BChE Inhibition: Determination of Steady-State Inhibition Constants

We assessed the mechanisms of AChE and BChE inhibition by performing a thorough analysis of enzyme kinetics. After a 5 min incubation at 25 °C (for temperature equilibration) with three increasing concentrations of inhibitor and six decreasing substrate concentrations, the residual enzyme activity was measured as described above for enzymatic assays. Linear regression of 1/V versus 1/[S] double-reciprocal (Lineweaver–Burk) plots was used to determine the inhibition constants for the competitive component (*K*_i_) and non-competitive component (α*K*_i_).

#### 3.2.2. Propidium Iodide Displacement Studies

We used a fluorescence method to detect the propensity of the test compounds to competitively displace propidium iodide (Sigma-Aldrich), a selective ligand of the AChE PAS [110,111]. Donepezil and tacrine (Sigma-Aldrich) were employed as the positive controls (i.e., reference compounds). The enzyme was electric eel AChE (*Ee*AChE type VI-S, lyophilized powder, Sigma-Aldrich). We selected this source of AChE for consistency with our other reports and because of the purity, specific activity, and lower cost compared to human AChE. Moreover, a 3D alignment of *Ee*AChE (PDB: 1C2O) and human AChE (PDB: 4EY7) using the MUSTANG procedure [112] in YASARA-Structure 18.4.24 for Windows [113] yielded close agreement between the two structures (RMSD 0.623 Å over 527 aligned residues and 88.6% sequence identity).

The assay is based on the high level of fluorescence intensity of propidium iodide bound with AChE, which decreases in the presence of test compounds that competitively displace propidium iodide from the AChE PAS [22,114]. Specifically [115], *Ee*AChE (7 μM final concentration) was incubated with the test compound (20 μM in 1 mM Tris-HCl buffer pH 8.0, 25 °C, for 15 min). Propidium iodide (final concentration 8 μM) was then added for a further 15 min incubation, and the fluorescence spectrum was taken (530 nm excitation and 600 nm emission) using a FLUOStar Optima microplate reader. The same concentration of propidium iodide in the Tris buffer was used as the blank. Triplicate determinations were recorded and the results were calculated via the following equation:

% Displacement = 100 − (IF_AChE+ Propidium + inhibitor_/IF_AChE + Propidium_) × 100,

where IF_AChE + Propidium_ = fluorescence intensity of propidium iodide associated with AChE in the absence of the test compound (taken as 100%), and IF_AChE + Propidium + inhibitor_ = fluorescence intensity of propidium iodide associated with AChE in the presence of the test compound.

#### 3.2.3. Inhibition of β-Amyloid (1–42) (Aβ_42_) Self-Aggregation

The influence of the inhibitory effect of the test compounds toward Aβ_42_ self-aggregation was determined using the thioflavin T (ThT) fluorescence method [79,83] with minor modifications. This assay is based on a specific interaction between the fluorescent dye thioflavin T that binds to the β-sheets of assembled amyloid fibrils leading to a significant increase in fluorescence signal [116]. Therefore, the decrease in ThT fluorescence correlates with the ability of the studied compounds to inhibit the formation of amyloid aggregates.

One mg of lyophilized HFIP-pretreated Aβ_42_ from BACHEM (Bubendorf, Switzerland) was dissolved in DMSO to obtain a stable initial solution ([Aβ_42_] = 500 μM); then it was aliquoted and stored at −20 °C.

For the measurement of Aβ_42_ self-aggregation and amyloid fibril inhibition studies by the tested compounds, aliquots of 500 μM Aβ_42_ stock solution were diluted in 215 mM Na-phosphate buffer pH = 8.0 to a final concentration of 50 μM Aβ_42_. Then the samples were incubated for 24 h at 37 °C without stirring in the absence (base level of Aβ_42_ self-aggregation, control) or presence of the test compounds. Myricetin and propidium iodide were used as reference compounds (positive controls). All compounds were used in a concentration of 100 µM. To quantify Aβ_42_ fibril formation, after incubation, 5 μM ThT in 50 mM glycine-NaOH buffer pH 8.5 was added to the solutions to a final concentration of 4 μM ThT and the fluorescence was measured at 440 nm (excitation) and 485 nm (emission). Analyses were performed with a FLUOStar Optima microplate reader (LabTech, Ortenberg, Germany). The blanks consisted of 215 mM Na-phosphate buffer, pH = 8.0, 20% (*v*/*v*) DMSO or test compounds, respectively. Each assay was run in triplicate.

The inhibition (%) of Aβ_42_ self-aggregation by the test compounds was calculated using the following equation:

% inhibition = 100 − (IF_i_/IF_o_) × 100,

where IF_i_ and IF_o_ are the fluorescence intensities obtained for Aβ_42_ in the presence and in the absence of inhibitor, respectively, after subtracting the fluorescence of respective blanks.

#### 3.2.4. Antioxidant Activity

##### ABTS Radical Cation Scavenging Activity Assay

Radical scavenging activity of the compounds was assessed using the ABTS radical cation (ABTS^•+^) decolorization assay [90] with some modifications [91]. ABTS (2,2′-azinobis-(3-ethylbenzothiazoline-6-sulfonic acid) was purchased from Tokyo Chemical Industry Co., Ltd. (Tokyo, Japan); potassium persulfate (dipotassium peroxodisulfate), Trolox (6-hydroxy-2,5,7,8-tetramethylchroman-2-carboxylic acid), and HPLC-grade ethanol and DMSO were obtained from Sigma-Aldrich (Saint Louis, MO, USA). Aqueous solutions were prepared using deionized water. All tested compounds were dissolved in DMSO.

The solution of cation radical ABTS^•+^ was produced by incubation of an aqueous solution of 7 mM ABTS and 2.45 mM potassium persulfate solution in equal quantities for 12–16 h at room temperature in the dark. Radical scavenging capacity of the compounds was analyzed by mixing 10 μL of compound with 240 μL of ABTS^•+^ working solution in ethanol (100 μM final concentration). The reaction was monitored for an hour at 30 °C with an interval of 10 min. Data are given for 1 h of incubation of compounds with ABTS^•+^. The reduction in absorbance was measured spectrophotometrically at 734 nm using an xMark microplate UV/VIS spectrophotometer (Bio-Rad, Hercules, CA, USA). Ethanol blanks were run in each assay. Values were obtained from five replicates of each sample and three independent experiments.

The standard AO Trolox was used as a reference compound. AO capacity as Trolox equivalent (TEAC) values was determined as the ratio between the slopes obtained from the linear correlation of the ABTS radical absorbance with the concentrations of tested compounds and Trolox. Ascorbic acid was used as a positive control. For the test compounds that reduced ABTS^•+^ absorbance by more than 60% at 100 µM, we also determined the IC_50_ values (compound concentration required for 50% reduction of the ABTS radical). The compounds were tested in the concentration range of 0.5–100 µM.

##### FRAP Assay

The FRAP (ferric reducing antioxidant power) assay proposed by Benzie and Strain [92] was modified to be performed in 96-well microplates as described in detail in [53]. The FRAP reagent contained 2.5 mL of 10 mM TPTZ (2,4,6-tris(2-pyridyl)-s-triazine, Sigma-Aldrich) solution in 40 mM HCl, 2.5 mL of 20 mM FeCl_3_ (Sigma-Aldrich) in distilled water and 25 mL of 0.3 M acetate buffer (pH 3.6). Aliquots of 10 µL of the test compound dissolved in DMSO (0.5 mM) were placed in quadruplicate. Absorbance was measured at a wavelength of 593 nm after 60 min incubation at 37 °C. In each case, Trolox was used as a reference compound to obtain the standard curve. The AO activity was calculated with respect to the activity of Trolox and expressed as Trolox equivalents (TE)—the values calculated as the ratio of the concentrations of Trolox and the test compound resulting in the same effect on ferric reducing activity.

#### 3.2.5. Metal Chelating Properties

The complexing studies were made in ethanol at 25 °C using a UV-Vis spectrophotometer Shimadzu UV-2600 (Shimadzu Corporation, Kyoto, Japan) with wavelength ranging from 190 to 600 nm. FeCl_2_·4H_2_O (99+%, Acros Organics by Thermo Fisher Scientific (Kandel, Germany)), CuCl_2_ (98%, Alfa Aesar by Thermo Fisher Scientific (Kandel, Germany)), or Zn(NO_3_)_2_·6H_2_O (98%, Alfa Aesar by Thermo Fisher Scientific (Kandel, Germany) ethanol solutions at 200 µM were prepared using volumetric flasks. The test compounds were dissolved in ethanol at 400 µM concentration. To a mixture of 0.5 mL of test compound solution (40 μM final concentration) and 3.5 mL of ethanol, 1 mL of CuCl_2_ solution (FeCl_2_·4H_2_O or Zn(NO_3_)_2_·6H_2_O; 40 μM final concentration) was added. The solution was incubated at 25 °C for 30 min, and the absorption spectra were recorded at 25 °C in a 1 cm quartz cell. The control was prepared by mixing 0.5 mL of test compound solution and 4.5 mL of ethanol.

The difference UV-Vis spectra resulting from complex formation were obtained by numerical subtraction of the spectra of the metal alone and the compound alone (at the same concentration used in the mixture) from the spectra of the mixture. A fixed concentration of tested compound (20 µM) was mixed with increasing concentrations of metal ions (2–34 µM), and the difference UV-Vis spectra were examined to investigate the ratio of ligand/metal in the complex.

### 3.3. Statistical Analyses

Plots, linear regressions, and IC_50_ values were determined using Origin 6.1 for Windows, OriginLab (Northampton, MA, USA). All tests were performed at least in triplicate in three independent experiments. Results are presented as mean ± SEM calculated using GraphPad Prism version 6.05 for Windows (San Diego, CA, USA).

### 3.4. Molecular Modeling Studies

#### 3.4.1. Preparation of the Molecules

Estimations of p*K*_a_ values of the ligands were performed with the Calculator Plugins of MarvinSketch 21.14.0, ChemAxon (http://www.chemaxon.com, accessed on 5 December 2022). Because the p*K*_a_ values of the tacrine fragment for all considered compounds was estimated as 8.89, all conjugates were used for all further calculations with a protonated endocyclic nitrogen atom of the tacrine fragment. For compounds **10** with the imine fragment, both protonated and non-protonated species were considered for molecular docking studies.

The ligand molecules were optimized using a DFT quantum chemistry method (B3LYP/6-31G*, GAMESS-US [117] software). For molecular docking, the optimized structures of the ligands were used with partial atomic charges derived from QM results according to the Löwdin scheme [118].

Protein targets for docking were as follows: X-ray structures of human AChE co-crystallized with donepezil (PDB: 4EY7) [119], an optimized X-ray structure of human BChE (PDB: 1P0I) [120,121], and all conformers of the Aβ_42_ structure PDB ID 1IYT [85].

#### 3.4.2. Molecular Docking

Molecular docking was performed with AutoDock 4.2.6 software [122]. The grid box for docking included the entire active site gorge of AChE (22.5 Å × 22.5 Å × 22.5 Å grid box dimensions) and BChE (15 Å × 20.25 Å × 18 Å grid box dimensions) with a grid spacing of 0.375 Å. The main Lamarckian Genetic Algorithm (LGA) [123] parameters were 256 runs, 25 × 10^6^ evaluations, 27 × 10^4^ generations, and a population size of 3000. Figures were prepared with PyMol (www.pymol.org, accessed on 21 July 2016).

#### 3.4.3. Quantum Chemical Analysis of AO Activity

DFT calculations were performed with Gaussian 16 [124] and the PRIRODA-19 program developed by D.N. Laikov [125]. A preliminary conformational search was carried out in Priroda software using the PBE0 functional [126] and the TZVP basis set [127] in the gas phase. The obtained conformations were used for optimization in the Gaussian package.

The optimized geometries were found using long-range-corrected functional CAM-B3LYP [128] in the 6-31++G(d,p) basis set [129]. All the optimizations were confirmed as stationary points by the absence of imaginary vibrational frequencies for the optimized geometries. To increase the accuracy of the thermochemical data calculation, the single-point energies of obtained optimized geometries with the 6-31++G(d,p) basis set were refined in the aug-cc-pVTZ basis set [130].

Calculations were performed for the gas phase and in ethanol solvent taking into account the SMD continuum solvation model [131].

MD calculations with QM/MM potentials (QM/MM-MD) were carried out using the common interface of the MD simulation package NAMD [132] with the quantum chemical package TeraChem [133] using the electrostatic injection scheme. The systems were prepared using VMD [134] software and the CGenFF program v. 2.4.0 [135] for parameterization of the model molecules. To mimic experimental conditions, a solvent box consisting of 1116 ethanol and 368 water molecules was used, based on the published ethanol solvent box [136]. For the classical MD and MM subsystem in the QM/MM-MD simulations, the CHARMM36 force field was used. During MD simulations, systems were maintained at constant temperature (298 K) and pressure (1 atm) (NPT ensemble) by using Langevin dynamics and the Nosé–Hoover barostat, with a 1 fs timestep. Periodic boundary conditions and PME electrostatics were applied. Prior to production QM/MM-MD runs, solvent equilibration was performed; during the 1 ns classical MD run, the coordinates of the solute molecules were fixed. The final size of the equilibrated system was 49 Å × 49 Å × 49 Å. The quantum subsystem included the model dimer with two ethanol and two water molecules forming hydrogen bonds with the solute. The QM subsystem was treated with the PBE0-D3 electron density functional and the cc-pvdz basis set. Accommodation of the starting systems took 20 ps of QM/MM-MD simulations, and then 50 ps of production runs were performed. Trajectories were analyzed using VMD and figures prepared using PyMol (www.pymol.org, accessed on 20 December 2022).

#### 3.4.4. Prediction of ADMET, Physicochemical, and PAINS Profiles

Lipophilicity (LogP_ow_) and aqueous solubility (pS_aq_) were estimated by the ALogPS 3.0 neural network model implemented in the OCHEM platform [137]. Human intestinal absorption (HIA) [138], blood-brain barrier distribution/permeability (LogBB) [139,140], and hERG-mediated cardiac toxicity risk (channel affinity p*K_i_* and inhibitory activity pIC_50_) [141] were estimated using the integrated online service for ADMET properties prediction [142]. This service implements predictive QSAR models based on accurate and representative training sets, fragmental descriptors, and artificial neural networks. The quantitative estimate of drug-likeness (QED) values [143] were calculated and the pan assay interference compounds (PAINS) alerts were checked using RDKit version 2020.03.4 software [144].

### 3.5. Cytotoxicity Studies

The cytotoxicity of the compounds was evaluated using a primary culture of mouse hepatocytes isolated at the Center for Biotechnology and Bioengineering, Ural Federal University, Ekaterinburg, Russia, according to the standard method [145]. Cells were seeded in 96-well plates in the inoculum dose of 1 × 10^4^ cells/mL and cultured for 24 h in Dulbecco’s Modified Eagle Medium (DMEM), with 1% (*w*/*v*) glutamine in the presence of 10% (*v*/*v*) fetal bovine serum and gentamicin (50 mg/L) at 37 °C, with a humidified atmosphere of 5% (*v*/*v*) CO_2_. Cell viability was assessed using the standard MTT assay [102]. The compounds were dissolved in DMSO and then in a nutrient medium, after which they were added to the wells with cells at final concentrations of 10^−7^, 10^−6^, 10^−5^, and 10^−4^ M. Hepatocytes were exposed to test compounds for 24 h at 37 °C, 95% relative humidity and 5% (*v*/*v*) CO_2_ atmosphere. After incubation, the medium was replaced with 100 μL of fresh medium containing MTT (0.5 mg/mL). The plates were then incubated at 37°C in a CO_2_ incubator for 2 h. Then the medium with MTT was removed and DMSO was added to dissolve the formazan crystals.

The results of the MTT assay were evaluated on a Tecan Infinite M200 PRO (Tecan Austria GmbH, Grödig, Austria) plate spectrophotometer at 570 nm by comparing the optical density of a formazan solution in the assay and control wells. The cell viability was expressed as the percentage of untreated control. The results were obtained from three independent experiments performed in six replicates. The IC_50_ (μM) values were calculated by the AAT Bioquest-calculator: (https://www.aatbio.com/tools/ic50-calculator, accessed on 1 December 2022) and expressed as a mean ± standard error of the mean (SEM). Statistical analysis was carried out using Microsoft Excel 2007 (Microsoft Corporation, Redmond, WA, USA) and Statistica 2009 (Tulsa, OK, USA) programs.

## 4. Conclusions

In summary, we extended a series of previously synthesized conjugates of tacrine and salicylamide, which displayed the potential to be multifunctional anti-AD agents, by variation of the structure of the salicylamide fragment and using salicylic aldehyde to synthesize the corresponding salicylimine derivatives. This procedure also resulted in an expansion of the spectrum of biological activities exhibited by the compounds.

All new conjugates exhibited high dual anticholinesterase activity with selectivity toward BChE. The structure of the salicylamide fragment had only a slight effect on the inhibitor activity. The most active hybrids were the salicylimine derivatives. All conjugates had poor anti-CES activity, suggesting a low tendency to exhibit potential unwanted drug-drug interactions in clinical use.

Detailed kinetics studies demonstrated that the conjugates exerted mixed-type reversible inhibition of AChE and BChE. Molecular docking indicated dual binding to the CAS and PAS of AChE, and propidium iodide displacement experiments suggested that the compounds had the potential to block *β*-amyloid aggregation induced by AChE.

All conjugates were active as inhibitors of A*β*_42_ self-aggregation in the thioflavin test, and both the structure of the salicylic fragment and the length of the spacer affected the inhibitory activity. In each group, the ability to inhibit A*β*_42_ self-aggregation increased with spacer elongation. Salicylimines **10a–c** were the most active group, followed by salicylamides **5a–c**. Salicylimine **10c** (80.1 ± 5.6%) and salicylamide **5c** (71.9 ± 5.7%) with a spacer length (CH_2_)_8_ were the lead compounds, which inhibited Aβ_42_ self-aggregation comparably to the reference compound Myricetin (74.7 ± 5.2%). These results agreed with molecular docking to A*β*_42_, using all conformers of Aβ_42_ as the targets. The strongest binding was obtained for **10c** docked to the second conformer of Aβ_42_. Binding of **10c** and **5c** to the second conformer had the highest number of contacts to the HHQK domain, which was shown to be a key motif for transition of the α-helix to the more stable and neurotoxic β-sheet conformation.

The conjugates exhibited high ABTS^•+^-scavenging activity but were almost inactive in the FRAP test. ABTS^•+^-scavenging activity depended on the structure of the salicylic derivative fragment. The most active were salicylamides **5a–c**, followed by salicylimines **10a–c**. Methylation of the OH group (**6a–c**) and introduction of F atoms (**7a–c**, **8a–c**) led to a sharp decrease or disappearance of AO activity, in agreement with quantum chemical and MD calculations with QM/MM potentials.

All conjugates were effective chelators of Cu^2+^, Fe^2+^, and Zn^2+^, with molar ratios of compound:metal = 2:1 for **5b** and close to 1:1 for **10b**.

Conjugates exhibited cytotoxicity comparable (**6c** and **8b**) or lower (**5b**, **7b**, and **10c**) than that of tacrine on mouse hepatocytes.

Overall, the obtained results allowed us to consider the synthesized conjugates as promising multifunctional agents for the potential treatment of AD.

## Figures and Tables

**Table 1:** Esterase profiles of conjugates **5**–**8**, **10** of general formula (**I**), their ability to displace propidium from the *Ee*AChE PAS, and their inhibition of Aβ_42_ self-aggregation.

Compound			Inhibitory Activity against AChE, BChE, and CES IC_50_ (µM) or Inhibition at 20 µM (%) ^1^			Propidium Displacement from *Ee*AChE PAS, (%) ^1^	Inhibition of Aβ_42_ Self- Aggregation, (%) ^2^
No	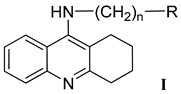 Where R	n	Human Erythrocyte AChE	Equine Serum BChE	Porcine Liver CES ^1^		
Salicylamides							
**5a**	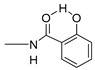	4	1.17 ± 0.05	0.0107 ± 0.0005	1.6 ± 1.1%	11.8 ± 0.9	20.7 ± 1.6
**5b**		6	0.294 ± 0.008	0.0119 ± 0.0001	19.5 ± 0.9%	12.6 ± 1.0	41.5 ± 2.9
**5c**		8	0.224 ± 0.017	0.0104 ± 0.0013	29.5 ± 2.5%	14.2 ± 1.1	71.9 ± 5.7
Methoxybenzoylamides							
**6a**	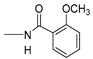	4	1.92 ± 0.13	0.0105 ± 0.0007	3.4 ± 0.7%	10.2 ± 0.9	12.3 ± 0.9
**6b**		6	0.646 ± 0.041	0.0377 ± 0.0031	8.0 ± 1.3%	12.5 ± 0.8	30.8 ± 2.1
**6c**		8	0.150 ± 0.011	0.0475 ± 0.0032	10.6 ± 1.2%	14.1 ± 0.9	55.6 ± 4.4
Difluorosalicylamides							
**7a**	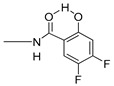	4	0.500 ± 0.030	0.0291 ± 0.0022	4.3 ± 0.9%	10.4 ± 0.9	17.4 ± 1.2
**7b**		6	0.249 ± 0.017	0.0317 ± 0.0030	7.1 ± 1.0%	12.2 ± 0.9	24.1 ± 2.1
**7c**		8	0.188 ± 0.015	0.0226 ± 0.0018	8.6 ± 0.7%	13.6 ± 1.0	37.2 ± 2.9
Difluoromethoxybenzoylamides							
**8a**	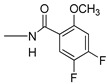	4	1.22 ± 0.07	0.0547 ± 0.0034	6.5 ± 1.1%	13.2 ± 1.0	10.4 ± 0.9
**8b**		6	0.712 ± 0.067	0.0702 ± 0.0042	6.3 ± 0.9%	13.8 ± 0.9	29.7 ± 2.3
**8c**		8	0.142 ± 0.011	0.0317 ± 0.0024	19.8 ± 3.1%	15.1 ± 1.2	54.1 ± 4.3
Salicylimines							
**10a**	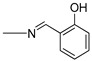	4	0.743 ± 0.044	0.127 ± 0.006	20.8 ± 0.4%	18.6 ± 1.6	57.9 ± 4.6
**10b**		6	0.103 ± 0.006	0.0354 ± 0.0017	17.9 ± 0.8%	18.2 ± 1.4	67.9 ± 5.4
**10c**		8	0.0826 ± 0.0046	0.0156 ± 0.0012	23.0 ± 0.3%	19.8 ± 1.7	80.1 ± 5.6
N-hexylsalicylamide [59] 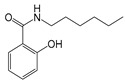			n.a.	10.6 ± 0.8%	4.9 ± 1.0%	n.d.	n.a
Salicylic acid			n.a.	47.6 ± 3.2	8.9 ± 0.6%	n.d.	6.5 ± 1.2
Tacrine			0.601 ± 0.047	0.0295 ± 0.0020	n.a.	3.1 ± 0.2	5.9 ± 0.5
BNPP			n.a.	n.a.	1.80 ± 0.11	n.d.	n.d.
Donepezil			0.040 ± 0.004	19.2 ± 3.0	n.a.	11.9 ± 0.9	n.d.
Myricetin			n.d.	n.d.	n.d.	n.d.	74.7 ± 5.2
Propidium iodide			n.d.	n.d.	n.d.	n.d.	90.7 ± 7.1

^1^ compound concentration 20 µM; *Ee*AChE = *Electrophorus electricus* (electric eel) AChE. ^2^ Inhibition of Aβ_42_ self-aggregation (50 µM) by the tested compound at 100 µM concentration. n.d.—not determined. n.a.—not active. Data are expressed as mean ± SEM, *n* = 3.

**Table 2:** Inhibition constants of cholinesterases by conjugates **5c**–**8c**, **10c** ^1^.

Compound	AChE		eqBChE	
	*K*_i_ (µM)	α*K*_i_ (µM)	*K*_i_ (µM)	α*K*_i_ (µM)
**5c**	0.152 ± 0.012	0.263 ± 0.018	0.0106 ± 0.0007	0.0171 ± 0.0013
**6c**	0.104 ± 0.004	0.213 ± 0.012	0.0188 ± 0.0011	0.0314 ± 0.0025
**7c**	0.125 ± 0.003	0.134 ± 0.009	0.0173 ± 0.0006	0.0243 ± 0.0001
**8c**	0.0536 ± 0.0025	0.0998 ± 0.0058	0.0177 ± 0.0007	0.0187 ± 0.0013
**10c**	0.0299 ± 0.0021	0.0644 ± 0.0040	0.0084 ± 0.0006	0.0152 ± 0.0009

^1^ Values for *K*_i_ (competitive inhibition constant) and α *K*_i_ (non-competitive inhibition constant) were determined from analyses of slopes of 1/V versus 1/S at various inhibitor concentrations. Values (means ± SEM) are from at least three separate experiments.

**Table 3:** AO properties of conjugates **5**–**8**, **10** of general formula (**I**).

Compound			ABTS^•+^ Scavenging Activity		
No	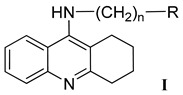 Where R	n	TEAC ^1^	IC_50_, μM ^2^	FRAP, TE ^3^
Salicylamides					
**5a**	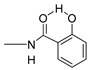	4	0.73 ± 0.04	25.3 ± 1.1	0.02
**5b**		6	0.90 ± 0.05	21.3 ± 1.5	n.a.
**5c**		8	0.75 ± 0.04	25.5 ± 1.1	n.a.
Methoxybenzoylamides					
**6a**	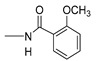	4	0.02 (n.a.)	n.d.	0.03
**6b**		6	n.a.	n.d.	0.02
**6c**		8	n.a.	n.d.	n.a.
Difluorosalicylamides					
**7a**	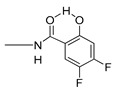	4	0.060 ± 0.002	>200	n.a.
**7b**		6	0.030 ± 0.001	>200	0.03
**7c**		8	0.040 ± 0.002	>200	0.06
Difluoromethoxybenzoylamides					
**8a**	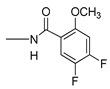	4	n.a.	n.d.	0.03
**8b**		6	n.a.	n.d.	0.02
**8c**		8	n.a.	n.d.	0.02
Salicylimines					
**10a**	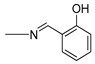	4	0.42 ± 0.03	35.8 ± 1.9	0.02
**10b**		6	0.47 ± 0.04	31.8 ± 1.6	0.03
**10c**		8	0.42 ± 0.04	34.2 ± 1.8	n.a.
N-hexylsalicylamide [59] 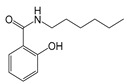			0.39 ± 0.04	46.5 ± 2.3	n.d.
Salicylic acid			n.a.	n.a.	0.02
Tacrine			n.a.	n.a.	n.a.
Trolox			1.00	19.8 ± 0.8	1.00
Ascorbic acid			0.96 ± 0.03	22.6 ± 1.2	1.28 ± 0.11

^1^ TEAC (Trolox Equivalent AO Capacity) was determined from the ratio of the slopes of the concentration-response curves test compound/Trolox. ^2^ Compound concentration required for 50% reduction of the ABTS radical cation radical. ^3^ TE (Trolox Equivalent in FRAP method)—the values were calculated as the ratio of the concentrations of Trolox and the test compound, resulting in the same effect. n.d.—not determined; n.a.—not active. Data are expressed as mean ± SEM, *n* = 3.

**Table 4:** Comparison of calculated BDE and IP with AO activity (ABTS: TEAC and IC_50_) for selected conjugates.

Compound	Ethanol BDE, kcal/mol	Ethanol IP, kcal/mol	TEAC	IC_50_, μM
**5a**	81.7	124.7	0.72 ± 0.04	25.3 ± 1.1
**10a**	84.7	126.1	0.42 ± 0.03	35.8 ± 1.9
**6a**	88.9	124.3	0.02 (n.a.)	n.d.
**7a**	82.0	121.9	0.060 ± 0.002	>200

Experimental ABTS data repeated from Table 3 to facilitate comparisons; data are mean ± SEM (*n* = 3). BDE values for **5a**, **10a**, and **6a** are inversely correlated with TEAC values; the row for **7a** is shaded gray to emphasize its outlying from this trend.

**Table 5:** Data on UV spectra of **5b**, **6b**, **7b**, **8b**, and **10b** and their metal complexes.

Sample	λ_max_, nm	Shift of Absorbance ^1^, nm		
		Cu(II)	Fe(II)	Zn(II)
**5b** [59]	337, 323, 308	352	351	351
	237	254	254	254
**6b**	336	352	352	352
	241	254	254	253
**7b**	337	352	352	352
	246	253	253	-
**8b**	336	351	351	351
	245	254	255	251
**10b**	323	352	352	352
	246	255	254	254

^1^ The shift of absorbance values was calculated by subtraction of the sum of spectra of the compound and corresponding ion from the spectra of the mixture.

**Table 6:** Cytotoxicity of conjugates **5b**, **6c**, **7b**, **8b**, **10c** and tacrine against mouse hepatocytes.

Compound	IC_50,_ μM (Mean ± SEM, *n* = 3)
**5b**	4.89 ± 0.08
**6c**	3.81 ± 0.07
**7b**	15.16 ± 0.09
**8b**	3.95 ± 0.22
**10c**	5.87 ± 0.14
Tacrine	3.78 ± 0.15

**Table 7:** Predicted ADMET and physicochemical profiles of compounds **5–8** and **10**.

Compound	MW	LogP_ow_	pS_aq_	LogBB	HIA, %	hERG *pK_i_*	hERG *pIC*_50_	QED
**5a**	389.50	4.38	5.51	–0.12	95	4.86	6.03	0.52
**5b**	417.55	5.14	6.12	–0.13	95	4.79	6.15	0.42
**5c**	445.61	5.80	6.80	0.25	95	5.18	6.60	0.32
**6a**	403.53	4.62	5.82	0.23	100	4.86	6.43	0.53
**6b**	431.58	5.36	6.46	0.23	100	4.79	6.56	0.41
**6c**	459.63	6.02	7.11	0.59	100	5.18	7.03	0.31
**7a**	425.48	4.51	5.90	–0.03	100	4.93	5.63	0.48
**7c**	481.59	5.97	7.50	0.34	100	5.25	6.11	0.28
**7b**	453.53	5.26	6.77	–0.04	100	4.87	5.73	0.37
**8a**	439.51	4.75	6.18	0.06	100	4.93	5.74	0.48
**8b**	467.56	5.52	7.03	0.05	100	4.87	5.84	0.37
**8c**	495.62	6.19	7.77	0.43	100	5.25	6.22	0.28
**10a**	373.50	4.97	5.83	0.02	89	5.16	5.76	0.45
**10b**	401.55	5.61	6.63	–0.07	89	5.08	6.16	0.35
**10c**	429.61	6.19	7.31	0.31	89	5.44	6.59	0.26
Tacrine	198.27	2.95	1.52	0.00	93	4.98	4.98	0.71

MW—molecular weight; LogP_ow_—octanol-water partition coefficient; pS_aq_—aqueous solubility [−log(M)]; LogBB—blood–brain barrier distribution; HIA—human intestinal absorption [%]; hERG *pK_i_*—hERG potassium channel affinity [−log(M)]; hERG *pIC*_50_—hERG potassium channel inhibitory activity [−log(M)]; QED—quantitative estimate of drug-likeness.

## Data Availability

Not applicable.

## Supplementary Materials

The following supporting information can be downloaded at: https://www.mdpi.com/article/10.3390/ijms24032285/s1.

## References

[1] Huang Y., Mucke L. (2012). Alzheimer Mechanisms and Therapeutic Strategies. Cell.

[2] Rosenberg R.N., Lambracht-Washington D., Yu G., Xia W. (2016). Genomics of Alzheimer Disease. JAMA Neurol..

[3] McDade E., Bateman R.J. (2017). Stop Alzheimer’s before it starts. Nature.

[4] Vaz M., Silvestre S. (2020). Alzheimer’s disease: Recent treatment strategies. Eur. J. Pharmacol..

[5] Cavalli A., Bolognesi M.L., Minarini A., Rosini M., Tumiatti V., Recanatini M., Melchiorre C. (2008). Multi-Target-Directed Ligands to Combat Neurodegenerative Diseases. J. Med. Chem..

[6] Albertini C., Salerno A., Sena Murteira Pinheiro P., Bolognesi M.L. (2021). From combinations to multitarget-directed ligands: A continuum in Alzheimer’s disease polypharmacology. Med. Res. Rev..

[7] Li X., Li X., Liu F., Li S., Shi D. (2021). Rational Multitargeted Drug Design Strategy from the Perspective of a Medicinal Chemist. J. Med. Chem..

[8] Martinez A., Castro A. (2006). Novel cholinesterase inhibitors as future effective drugs for the treatment of Alzheimer’s disease. Expert Opin. Investig. Drugs.

[9] Agatonovic-Kustrin S., Kettle C., Morton D.W. (2018). A molecular approach in drug development for Alzheimer’s disease. Biomed. Pharmacother..

[10] Furukawa-Hibi Y., Alkam T., Nitta A., Matsuyama A., Mizoguchi H., Suzuki K., Moussaoui S., Yu Q.-S., Greig N.H., Nagai T. (2011). Butyrylcholinesterase inhibitors ameliorate cognitive dysfunction induced by amyloid-β peptide in mice. Behav. Brain Res..

[11] Nordberg A., Ballard C., Bullock R., Darreh-Shori T., Somogyi M. (2013). A Review of Butyrylcholinesterase as a Therapeutic Target in the Treatment of Alzheimer’s Disease. Prim. Care Companion CNS Disord..

[12] Lane R.M., Potkin S.G., Enz A. (2005). Targeting acetylcholinesterase and butyrylcholinesterase in dementia. Int. J. Neuropsychopharmacol..

[13] Hardy J., Bogdanovic N., Winblad B., Portelius E., Andreasen N., Cedazo-Minguez A., Zetterberg H. (2014). Pathways to Alzheimer’s disease. J. Intern. Med..

[14] Ahmed M., Davis J., Aucoin D., Sato T., Ahuja S., Aimoto S., Elliott J.I., Van Nostrand W.E., Smith S.O. (2010). Structural conversion of neurotoxic amyloid-beta(1–42) oligomers to fibrils. Nat. Struct. Mol. Biol..

[15] Cleary J.P., Walsh D.M., Hofmeister J.J., Shankar G.M., Kuskowski M.A., Selkoe D.J., Ashe K.H. (2005). Natural oligomers of the amyloid-β protein specifically disrupt cognitive function. Nat. Neurosci..

[16] Selkoe D.J., Selkoe D.J., Triller A., Christen Y. (2008). Soluble Oligomers of the Amyloid β-Protein: Impair Synaptic Plasticity and Behavior. Synaptic Plasticity and the Mechanism of Alzheimer’s Disease.

[17] Jiang L., Huang M., Xu S., Wang Y., An P., Feng C., Chen X., Wei X., Han Y., Wang Q. (2016). Bis(propyl)-cognitin Prevents beta-amyloid-induced Memory Deficits as Well as Synaptic Formation and Plasticity Impairments via the Activation of PI3-K Pathway. Mol. Neurobiol..

[18] Hu S., Xian Y., Fan Y., Mak S., Wang J., Tang J., Pang Y., Pi R., Tsim K.W., Liu F. (2020). Significant combination of Aβ aggregation inhibitory and neuroprotective properties in silico, in vitro and in vivo by bis(propyl)-cognitin, a multifunctional anti-Alzheimer’s agent. Eur. J. Pharmacol..

[19] Jeremic D., Jiménez-Díaz L., Navarro-López J.D. (2021). Past, present and future of therapeutic strategies against amyloid-β peptides in Alzheimer’s disease: A systematic review. Ageing Res. Rev..

[20] Moran M.A., Mufson E.J., Gomez-Ramos P. (1994). Cholinesterases colocalize with sites of neurofibrillary degeneration in aged and Alzheimer’s brains. Acta Neuropathol..

[21] Inestrosa N.C., Alvarez A., Calderón F. (1996). Acetylcholinesterase is a senile plaque component that promotes assembly of amyloid beta-peptide into Alzheimer’s filaments. Mol. Psychiatry.

[22] De Ferrari G.V., Canales M.A., Shin I., Weiner L.M., Silman I., Inestrosa N.C. (2001). A Structural Motif of Acetylcholinesterase That Promotes Amyloid β-Peptide Fibril Formation. Biochemistry.

[23] Arce M.P., Rodríguez-Franco M.I., González-Muñoz G.C., Pérez C., López B., Villarroya M., López M.G., García A.G., Conde S. (2009). Neuroprotective and Cholinergic Properties of Multifunctional Glutamic Acid Derivatives for the Treatment of Alzheimer’s Disease. J. Med. Chem..

[24] Camps P., Formosa X., Galdeano C., Gómez T., Muñoz-Torrero D., Ramírez L., Viayna E., Gómez E., Isambert N., Lavilla R. (2010). Tacrine-based dual binding site acetylcholinesterase inhibitors as potential disease-modifying anti-Alzheimer drug candidates. Chem. Biol. Interact..

[25] Mesulam M., Geula C. (1994). Butyrylcholinesterase reactivity differentiates the amyloid plaques of aging from those of dementia. Ann. Neurol..

[26] Ramanan V.K., Risacher S.L., Nho K., Kim S., Swaminathan S., Shen L., Foroud T.M., Hakonarson H., Huentelman M.J., Aisen P.S. (2014). APOE and BCHE as modulators of cerebral amyloid deposition: A florbetapir PET genome-wide association study. Mol. Psychiatry.

[27] Darvesh S., Reid G.A. (2016). Reduced fibrillar β-amyloid in subcortical structures in a butyrylcholinesterase-knockout Alzheimer disease mouse model. Chem. Biol. Interact..

[28] Radi E., Formichi P., Battisti C., Federico A. (2014). Apoptosis and Oxidative Stress in Neurodegenerative Diseases. J. Alzheimers Dis..

[29] Kim G.H., Kim J.E., Rhie S.J., Yoon S. (2015). The Role of Oxidative Stress in Neurodegenerative Diseases. Exp. Neurobiol..

[30] Pohanka M. (2018). Oxidative stress in Alzheimer disease as a target for therapy. Bratisl. Med. J..

[31] Moreira P.I., Santos M.S., Oliveira C.R., Shenk J.C., Nunomura A., Smith M.A., Zhu X., Perry G. (2008). Alzheimer disease and the role of free radicals in the pathogenesis of the disease. CNS Neurol. Disord. Drug Targets.

[32] Chakrabarti S., Sinha M., Thakurta I., Banerjee P., Chattopadhyay M. (2013). Oxidative Stress and Amyloid Beta Toxicity in Alzheimer’s Disease: Intervention in a Complex Relationship by Antioxidants. Curr. Med. Chem..

[33] Rosini M., Simoni E., Bartolini M., Tarozzi A., Matera R., Milelli A., Hrelia P., Andrisano V., Bolognesi M.L., Melchiorre C. (2011). Exploiting the lipoic acid structure in the search for novel multitarget ligands against Alzheimer’s disease. Eur. J. Med. Chem..

[34] Barnham K.J., Bush A.I. (2008). Metals in Alzheimer’s and Parkinson’s Diseases. Curr. Opin. Chem. Biol..

[35] Bush A.I. (2008). Drug Development Based on the Metals Hypothesis of Alzheimer’s Disease. J. Alzheimers Dis..

[36] Valko M., Jomova K., Rhodes C.J., Kuča K., Musílek K. (2016). Redox- and non-redox-metal-induced formation of free radicals and their role in human disease. Arch. Toxicol..

[37] Strodel B., Coskuner-Weber O. (2019). Transition Metal Ion Interactions with Disordered Amyloid-beta Peptides in the Pathogenesis of Alzheimer’s Disease: Insights from Computational Chemistry Studies. J. Chem. Inf. Model..

[38] Xie S.-S., Wang X.-B., Li J.-Y., Yang L., Kong L.-Y. (2013). Design, synthesis and evaluation of novel tacrine–coumarin hybrids as multifunctional cholinesterase inhibitors against Alzheimer’s disease. Eur. J. Med. Chem..

[39] Santos M.A., Chand K., Chaves S. (2016). Recent progress in multifunctional metal chelators as potential drugs for Alzheimer’s disease. Coord. Chem. Rev..

[40] Mishra P., Kumar A., Panda G. (2019). Anti-cholinesterase hybrids as multi-target-directed ligands against Alzheimer’s disease (1998–2018). Bioorg. Med. Chem..

[41] Milelli A., De Simone A., Ticchi N., Chen H.H., Betari N., Andrisano V., Tumiatti V. (2017). Tacrine-based Multifunctional Agents in Alzheimer’s Disease: An Old Story in Continuous Development§. Curr. Med. Chem..

[42] Spilovska K., Korabecny J., Nepovimova E., Dolezal R., Mezeiova E., Soukup O., Kuca K. (2017). Multitarget tacrine hybrids with neuroprotective properties to confront Alzheimer’s disease. Curr. Top. Med. Chem..

[43] Przybyłowska M., Kowalski S., Dzierzbicka K., Inkielewicz-Stepniak I. (2019). Therapeutic Potential of Multifunctional Tacrine Analogues. Curr. Neuropharmacol..

[44] Makhaeva G.F., Kovaleva N.V., Boltneva N.P., Lushchekina S.V., Rudakova E.V., Stupina T.S., Terentiev A.A., Serkov I.V., Proshin A.N., Radchenko E.V. (2020). Conjugates of tacrine and 1,2,4-thiadiazole derivatives as new potential multifunctional agents for Alzheimer’s disease treatment: Synthesis, quantum-chemical characterization, molecular docking, and biological evaluation. Bioorg. Chem..

[45] Minarini A., Milelli A., Simoni E., Rosini M., Bolognesi M., Marchetti C., Tumiatti V. (2013). Multifunctional Tacrine Derivatives in Alzheimer’s Disease. Curr. Top. Med. Chem..

[46] Sameem B., Saeedi M., Mahdavi M., Shafiee A. (2017). A review on tacrine-based scaffolds as multi-target drugs (MTDLs) for Alzheimer’s disease. Eur. J. Med. Chem..

[47] Guzior N., Wieckowska A., Panek D., Malawska B. (2014). Recent Development of Multifunctional Agents as Potential Drug Candidates for the Treatment of Alzheimer’s Disease. Curr. Med. Chem..

[48] Singh M., Kaur M., Chadha N., Silakari O. (2016). Hybrids: A new paradigm to treat Alzheimer’s disease. Mol. Divers..

[49] Rosini M., Andrisano V., Bartolini M., Bolognesi M.L., Hrelia P., Minarini A., Tarozzi A., Melchiorre C. (2005). Rational Approach To Discover Multipotent Anti-Alzheimer Drugs. J. Med. Chem..

[50] Xie S.-S., Lan J.-S., Wang X.-B., Jiang N., Dong G., Li Z.-R., Wang K.D.G., Guo P.-P., Kong L.-Y. (2015). Multifunctional tacrine–trolox hybrids for the treatment of Alzheimer’s disease with cholinergic, antioxidant, neuroprotective and hepatoprotective properties. Eur. J. Med. Chem..

[51] Fernández-Bachiller M.I., Pérez C., González-Muñoz G.C., Conde S., López M.G., Villarroya M., García A.G., Rodríguez-Franco M.I. (2010). Novel Tacrine−8-Hydroxyquinoline Hybrids as Multifunctional Agents for the Treatment of Alzheimer’s Disease, with Neuroprotective, Cholinergic, Antioxidant, and Copper-Complexing Properties. J. Med. Chem..

[52] Nepovimova E., Korabecny J., Dolezal R., Babkova K., Ondrejicek A., Jun D., Sepsova V., Horova A., Hrabinova M., Soukup O. (2015). Tacrine–Trolox Hybrids: A Novel Class of Centrally Active, Nonhepatotoxic Multi-Target-Directed Ligands Exerting Anticholinesterase and Antioxidant Activities with Low In Vivo Toxicity. J. Med. Chem..

[53] Makhaeva G.F., Kovaleva N.V., Rudakova E.V., Boltneva N.P., Lushchekina S.V., Faingold I.I., Poletaeva D.A., Soldatova Y.V., Kotelnikova R.A., Serkov I.V. (2020). New Multifunctional Agents Based on Conjugates of 4-Amino-2,3-polymethylenequinoline and Butylated Hydroxytoluene for Alzheimer’s Disease Treatment. Molecules.

[54] Makhaeva G.F., Serkov I.V., Kovaleva N.V., Rudakova E.V., Boltneva N.P., Kochetkova E.A., Proshin A.N., Bachurin S.O. (2021). Novel conjugates of 4-amino-2,3-polymethylenequinolines and vanillin as potential multitarget agents for AD treatment. Mendeleev Commun..

[55] Mao F., Huang L., Luo Z., Liu A., Lu C., Xie Z., Li X. (2012). O-Hydroxyl- or o-amino benzylamine-tacrine hybrids: Multifunctional biometals chelators, antioxidants, and inhibitors of cholinesterase activity and amyloid-β aggregation. Bioorg. Med. Chem..

[56] Borges R.S., Pereira G.A.N., Vale J.K.L., França L.C.S., Monteiro M.C., Alves C.N., da Silva A.B.F. (2013). Design and Evaluation of 4-Aminophenol and Salicylate Derivatives as Free-Radical Scavenger. Chem. Biol. Drug Des..

[57] Borges R.S., Castle S.L. (2015). The antioxidant properties of salicylate derivatives: A possible new mechanism of anti-inflammatory activity. Bioorg. Med. Chem. Lett..

[58] Song Q., Li Y., Cao Z., Qiang X., Tan Z., Deng Y. (2019). Novel salicylamide derivatives as potent multifunctional agents for the treatment of Alzheimer’s disease: Design, synthesis and biological evaluation. Bioorg. Chem..

[59] Grishchenko M.V., Makhaeva G.F., Burgart Y.V., Rudakova E.V., Boltneva N.P., Kovaleva N.V., Serebryakova O.G., Lushchekina S.V., Astakhova T.Y., Zhilina E.F. (2022). Conjugates of Tacrine with Salicylamide as Promising Multitarget Agents for Alzheimer’s Disease. ChemMedChem.

[60] Ojima I. (2009). Fluorine in Medicinal Chemistry and Chemical Biology.

[61] Han J., Kiss L., Mei H., Remete A.M., Ponikvar-Svet M., Sedgwick D.M., Roman R., Fustero S., Moriwaki H., Soloshonok V.A. (2021). Chemical Aspects of Human and Environmental Overload with Fluorine. Chem. Rev..

[62] Zhou Y., Wang J., Gu Z., Wang S., Zhu W., Aceña J.L., Soloshonok V.A., Izawa K., Liu H. (2016). Next Generation of Fluorine-Containing Pharmaceuticals, Compounds Currently in Phase II–III Clinical Trials of Major Pharmaceutical Companies: New Structural Trends and Therapeutic Areas. Chem. Rev..

[63] O’Hagan D. (2010). Fluorine in health care: Organofluorine containing blockbuster drugs. J. Fluor. Chem..

[64] Isanbor C., O’Hagan D. (2006). Fluorine in medicinal chemistry: A review of anti-cancer agents. J. Fluor. Chem..

[65] Dolbier W.R. (2005). Fluorine chemistry at the millennium. J. Fluor. Chem..

[66] Berger R., Resnati G., Metrangolo P., Weber E., Hulliger J. (2011). Organic fluorine compounds: A great opportunity for enhanced materials properties. Chem. Soc. Rev..

[67] Purser S., Moore P.R., Swallow S., Gouverneur V. (2008). Fluorine in medicinal chemistry. Chem. Soc. Rev..

[68] Kirk K.L. (2006). Fluorine in medicinal chemistry: Recent therapeutic applications of fluorinated small molecules. J. Fluor. Chem..

[69] Luo W., Li Y.P., He Y., Huang S.L., Li D., Gu L.Q., Huang Z.S. (2011). Synthesis and evaluation of heterobivalent tacrine derivatives as potential multi-functional anti-Alzheimer agents. Eur. J. Med. Chem..

[70] Carlier P.R., Chow E.S.H., Han Y., Liu J., Yazal J.E., Pang Y.-P. (1999). Heterodimeric Tacrine-Based Acetylcholinesterase Inhibitors:  Investigating Ligand−Peripheral Site Interactions. J. Med. Chem..

[71] Makhaeva G.F., Rudakova E.V., Kovaleva N.V., Lushchekina S.V., Boltneva N.P., Proshin A.N., Shchegolkov E.V., Burgart Y.V., Saloutin V.I. (2019). Cholinesterase and carboxylesterase inhibitors as pharmacological agents. Russ. Chem. Bull..

[72] Makhaeva G.F., Rudakova E.V., Serebryakova O.G., Aksinenko A.Y., Lushchekina S.V., Bachurin S.O., Richardson R.J. (2016). Esterase profiles of organophosphorus compounds in vitro predict their behavior in vivo. Chem. Biol. Interact..

[73] Makhaeva G.F., Boltneva N.P., Lushchekina S.V., Serebryakova O.G., Stupina T.S., Terentiev A.A., Serkov I.V., Proshin A.N., Bachurin S.O., Richardson R.J. (2016). Synthesis, molecular docking and biological evaluation of N,N-disubstituted 2-aminothiazolines as a new class of butyrylcholinesterase and carboxylesterase inhibitors. Bioorg. Med. Chem..

[74] Makhaeva G.F., Lushchekina S.V., Boltneva N.P., Sokolov V.B., Grigoriev V.V., Serebryakova O.G., Vikhareva E.A., Aksinenko A.Y., Barreto G.E., Aliev G. (2015). Conjugates of γ-carbolines and phenothiazine as new selective inhibitors of butyrylcholinesterase and blockers of NMDA receptors for Alzheimer disease. Sci. Rep..

[75] Makhaeva G.F., Radchenko E.V., Palyulin V.A., Rudakova E.V., Aksinenko A.Y., Sokolov V.B., Zefirov N.S., Richardson R.J. (2013). Organophosphorus compound esterase profiles as predictors of therapeutic and toxic effects. Chem. Biol. Interact..

[76] Laizure S.C., Herring V., Hu Z., Witbrodt K., Parker R.B. (2013). The role of human carboxylesterases in drug metabolism: Have we overlooked their importance?. Pharmacotherapy.

[77] Tsurkan L.G., Hatfield M.J., Edwards C.C., Hyatt J.L., Potter P.M. (2013). Inhibition of human carboxylesterases hCE1 and hiCE by cholinesterase inhibitors. Chem. Biol. Interact..

[78] Lushchekina S.V., Kots E.D., Novichkova D.A., Petrov K.A., Masson P. (2016). Role of acetylcholinesterase in β-amyloid aggregation studied by accelerated molecular dynamics. BioNanoScience.

[79] Bartolini M., Bertucci C., Cavrini V., Andrisano V. (2003). β-Amyloid aggregation induced by human acetylcholinesterase: Inhibition studies. Biochem. Pharmacol..

[80] Bachurin S.O., Makhaeva G.F., Shevtsova E.F., Aksinenko A.Y., Grigoriev V.V., Shevtsov P.N., Goreva T.V., Epishina T.A., Kovaleva N.V., Pushkareva E.A. (2021). Conjugation of Aminoadamantane and γ-Carboline Pharmacophores Gives Rise to Unexpected Properties of Multifunctional Ligands. Molecules.

[81] Ghotbi G., Mahdavi M., Najafi Z., Moghadam F.H., Hamzeh-Mivehroud M., Davaran S., Dastmalchi S. (2020). Design, synthesis, biological evaluation, and docking study of novel dual-acting thiazole-pyridiniums inhibiting acetylcholinesterase and beta-amyloid aggregation for Alzheimer’s disease. Bioorg. Chem..

[82] Sanchez Montero J.M., Agis-Torres A., Solano D., Sollhuber M., Fernandez M., Villaro W., Gomez-Canas M., Garcia-Arencibia M., Fernandez-Ruiz J., Egea J. (2021). Analogues of cannabinoids as multitarget drugs in the treatment of Alzheimer’s disease. Eur. J. Pharmacol..

[83] Munoz-Ruiz P., Rubio L., Garcia-Palomero E., Dorronsoro I., del Monte-Millan M., Valenzuela R., Usan P., de Austria C., Bartolini M., Andrisano V. (2005). Design, synthesis, and biological evaluation of dual binding site acetylcholinesterase inhibitors: New disease-modifying agents for Alzheimer’s disease. J. Med. Chem..

[84] Bartolini M., Naldi M., Fiori J., Valle F., Biscarini F., Nicolau D.V., Andrisano V. (2011). Kinetic characterization of amyloid-beta 1–42 aggregation with a multimethodological approach. Anal. Biochem..

[85] Crescenzi O., Tomaselli S., Guerrini R., Salvadori S., D’Ursi A.M., Temussi P.A., Picone D. (2002). Solution structure of the Alzheimer amyloid beta-peptide (1–42) in an apolar microenvironment. Similarity with a virus fusion domain. Eur. J. Biochem..

[86] Safarizadeh H., Garkani-Nejad Z. (2019). Molecular docking, molecular dynamics simulations and QSAR studies on some of 2-arylethenylquinoline derivatives for inhibition of Alzheimer’s amyloid-beta aggregation: Insight into mechanism of interactions and parameters for design of new inhibitors. J. Mol. Graph. Model..

[87] Jokar S., Erfani M., Bavi O., Khazaei S., Sharifzadeh M., Hajiramezanali M., Beiki D., Shamloo A. (2020). Design of peptide-based inhibitor agent against amyloid-beta aggregation: Molecular docking, synthesis and in vitro evaluation. Bioorg. Chem..

[88] Giulian D., Haverkamp L.J., Yu J., Karshin W., Tom D., Li J., Kazanskaia A., Kirkpatrick J., Roher A.E. (1998). The HHQK domain of beta-amyloid provides a structural basis for the immunopathology of Alzheimer’s disease. J. Biol. Chem..

[89] Ariga T., Miyatake T., Yu R.K. (2010). Role of proteoglycans and glycosaminoglycans in the pathogenesis of Alzheimer’s disease and related disorders: Amyloidogenesis and therapeutic strategies—A review. J. Neurosci. Res..

[90] Re R., Pellegrini N., Proteggente A., Pannala A., Yang M., Rice-Evans C. (1999). Antioxidant activity applying an improved ABTS radical cation decolorization assay. Free Radic. Biol. Med..

[91] Makhaeva G.F., Elkina N.A., Shchegolkov E.V., Boltneva N.P., Lushchekina S.V., Serebryakova O.G., Rudakova E.V., Kovaleva N.V., Radchenko E.V., Palyulin V.A. (2019). Synthesis, molecular docking, and biological evaluation of 3-oxo-2-tolylhydrazinylidene-4,4,4-trifluorobutanoates bearing higher and natural alcohol moieties as new selective carboxylesterase inhibitors. Bioorg. Chem..

[92] Benzie I.F.F., Strain J.J. (1999). Ferric reducing/antioxidant power assay: Direct measure of total antioxidant activity of biological fluids and modified version for simultaneous measurement of total antioxidant power and ascorbic acid concentration. Methods Enzymol..

[93] Pan X., Wang H., Li C., Zhang J.Z.H., Ji C. (2021). MolGpka: A Web Server for Small Molecule pKa Prediction Using a Graph-Convolutional Neural Network. J. Chem. Inf. Model..

[94] Ilyasov I.R., Beloborodov V.L., Selivanova I.A., Terekhov R.P. (2020). ABTS/PP Decolorization Assay of Antioxidant Capacity Reaction Pathways. Int. J. Mol. Sci..

[95] Amic D., Stepanic V., Lucic B., Markovic Z., Dimitric Markovic J.M. (2013). PM6 study of free radical scavenging mechanisms of flavonoids: Why does O-H bond dissociation enthalpy effectively represent free radical scavenging activity?. J. Mol. Model..

[96] Marković Z., Tošović J., Milenković D., Marković S. (2016). Revisiting the solvation enthalpies and free energies of the proton and electron in various solvents. Comput. Theor. Chem..

[97] Yadav H.R., Choudhury A.R. (2017). Can C H⋯F C hydrogen bonds alter crystal packing features in the presence of N H⋯O C hydrogen bond?. J. Biomol. Struct..

[98] Cole J.C., Taylor R. (2022). Intermolecular Interactions of Organic Fluorine Seen in Perspective. Cryst. Growth Des..

[99] Perez C.A., Wei Y., Guo M. (2009). Iron-binding and anti-Fenton properties of baicalein and baicalin. J. Inorg. Biochem..

[100] Yang H.-L., Cai P., Liu Q.-H., Yang X.-L., Fang S.-Q., Tang Y.-W., Wang C., Wang X.-B., Kong L.-Y. (2017). Design, synthesis, and evaluation of salicyladimine derivatives as multitarget-directed ligands against Alzheimer’s disease. Bioorg. Med. Chem..

[101] Lou Y.-H., Wang J.-S., Dong G., Guo P.-P., Wei D.-D., Xie S.-S., Yang M.-H., Kong L.-Y. (2015). The acute hepatotoxicity of tacrine explained by 1H NMR based metabolomic profiling. Toxicol. Res..

[102] Mosmann T. (1983). Rapid colorimetric assay for cellular growth and survival: Application to proliferation and cytotoxicity assays. J. Immunol. Methods.

[103] Deng X., Wang Z., Liu J., Xiong S., Xiong R., Cao X., Chen Y., Zheng X., Tang G. (2017). Design, synthesis and biological evaluation of flavonoid salicylate derivatives as potential anti-tumor agents. RSC Adv..

[104] Shchegol’kov E.V., Shchur I.V., Burgart Y.V., Saloutin V.I., Solodnikov S.Y., Krasnykh O.P., Kravchenko M.A. (2016). A convenient and efficient approach to polyfluorosalicylic acids and their tuberculostatic activity. Bioorg. Med. Chem. Lett..

[105] Zhang H., Peng Y., Zhuo L., Wang Y., Zeng G., Wang S., Long L., Li X., Wang Z. (2022). Recent advance on pleiotropic cholinesterase inhibitors bearing amyloid modulation efficacy. Eur. J. Med. Chem..

[106] Gniazdowska E., Koźmiński P., Wasek M., Bajda M., Sikora J., Mikiciuk-Olasik E., Szymański P. (2017). Synthesis, physicochemical and biological studies of technetium-99m labeled tacrine derivative as a diagnostic tool for evaluation of cholinesterase level. Bioorg. Med. Chem..

[107] Cheng Z.-Q., Zhu K.-K., Zhang J., Song J.-L., Muehlmann L.A., Jiang C.-S., Liu C.-L., Zhang H. (2019). Molecular-docking-guided design and synthesis of new IAA-tacrine hybrids as multifunctional AChE/BChE inhibitors. Bioorg. Chem..

[108] Bornstein J.J., Eckroat T.J., Houghton J.L., Jones C.K., Green K.D., Garneau-Tsodikova S. (2011). Tacrine-mefenamic acid hybrids for inhibition of acetylcholinesterase. MedChemComm.

[109] Makhaeva G.F., Kovaleva N.V., Boltneva N.P., Lushchekina S.V., Astakhova T.Y., Rudakova E.V., Proshin A.N., Serkov I.V., Radchenko E.V., Palyulin V.A. (2020). New Hybrids of 4-Amino-2,3-polymethylene-quinoline and p-Tolylsulfonamide as Dual Inhibitors of Acetyl- and Butyrylcholinesterase and Potential Multifunctional Agents for Alzheimer’s Disease Treatment. Molecules.

[110] Taylor P., Lappi S. (1975). Interaction of fluorescence probes with acetylcholinesterase. Site and specificity of propidium binding. Biochemistry.

[111] Johnson G., Moore S.W. (2006). The peripheral anionic site of acetylcholinesterase: Structure, functions and potential role in rational drug design. Curr. Pharm. Des..

[112] Konagurthu A.S., Whisstock J.C., Stuckey P.J., Lesk A.M. (2006). MUSTANG: A multiple structural alignment algorithm. Proteins.

[113] Krieger E., Vriend G. (2014). YASARA View—Molecular graphics for all devices—From smartphones to workstations. Bioinformatics.

[114] Inestrosa N.C., Dinamarca M.C., Alvarez A. (2008). Amyloid-cholinesterase interactions. Implications for Alzheimer’s disease. FEBS J..

[115] Bachurin S.O., Makhaeva G.F., Shevtsova E.F., Boltneva N.P., Kovaleva N.V., Lushchekina S.V., Rudakova E.V., Dubova L.G., Vinogradova D.V., Sokolov V.B. (2019). Conjugates of methylene blue with gamma-carboline derivatives as new multifunctional agents for the treatment of neurodegenerative diseases. Sci. Rep..

[116] Biancalana M., Koide S. (2010). Molecular mechanism of Thioflavin-T binding to amyloid fibrils. Biochim. Biophys. Acta.

[117] Barca G.M.J., Bertoni C., Carrington L., Datta D., De Silva N., Deustua J.E., Fedorov D.G., Gour J.R., Gunina A.O., Guidez E. (2020). Recent developments in the general atomic and molecular electronic structure system. J. Chem. Phys..

[118] Löwdin P.-O., Per-Olov L. (1970). On the nonorthogonality problem. Advances in Quantum Chemistry.

[119] Cheung J., Rudolph M.J., Burshteyn F., Cassidy M.S., Gary E.N., Love J., Franklin M.C., Height J.J. (2012). Structures of Human Acetylcholinesterase in Complex with Pharmacologically Important Ligands. J. Med. Chem..

[120] Masson P., Lushchekina S., Schopfer L.M., Lockridge O. (2013). Effects of viscosity and osmotic stress on the reaction of human butyrylcholinesterase with cresyl saligenin phosphate, a toxicant related to aerotoxic syndrome: Kinetic and molecular dynamics studies. Biochem. J..

[121] Nicolet Y., Lockridge O., Masson P., Fontecilla-Camps J.C., Nachon F. (2003). Crystal structure of human butyrylcholinesterase and of its complexes with substrate and products. J. Biol. Chem..

[122] Morris G.M., Huey R., Lindstrom W., Sanner M.F., Belew R.K., Goodsell D.S., Olson A.J. (2009). AutoDock4 and AutoDock Tools4: Automated docking with selective receptor flexibility. J. Comput. Chem..

[123] Morris G.M., Goodsell D.S., Halliday R.S., Huey R., Hart W.E., Belew R.K., Olson A.J. (1998). Automated docking using a Lamarckian genetic algorithm and an empirical binding free energy function. J. Comput. Chem..

[124] Frisch M.J., Trucks G.W., Schlegel H.B., Scuseria G.E., Robb M.A., Cheeseman J.R., Scalmani G., Barone V., Petersson G.A., Nakatsuji H. (2016). Gaussian 16 Revision C. 01. 2016.

[125] Laikov D.N., Ustynyuk Y.A. (2005). PRIRODA-04: A quantum-chemical program suite. New possibilities in the study of molecular systems with the application of parallel computing. Russ. Chem. Bull..

[126] Adamo C., Barone V. (1999). Toward reliable density functional methods without adjustable parameters: The PBE0 model. J. Chem. Phys..

[127] Schäfer A., Huber C., Ahlrichs R. (1994). Fully optimized contracted Gaussian basis sets of triple zeta valence quality for atoms Li to Kr. J. Chem. Phys..

[128] Yanai T., Tew D.P., Handy N.C. (2004). A new hybrid exchange–correlation functional using the Coulomb-attenuating method (CAM-B3LYP). Chem. Phys. Lett..

[129] Rassolov V.A., Ratner M.A., Pople J.A., Redfern P.C., Curtiss L.A. (2001). 6-31G* basis set for third-row atoms. J. Comput. Chem..

[130] Papajak E., Zheng J., Xu X., Leverentz H.R., Truhlar D.G. (2011). Perspectives on Basis Sets Beautiful: Seasonal Plantings of Diffuse Basis Functions. J. Chem. Theory Comput..

[131] Marenich A.V., Cramer C.J., Truhlar D.G. (2009). Universal solvation model based on solute electron density and on a continuum model of the solvent defined by the bulk dielectric constant and atomic surface tensions. J. Phys. Chem. B.

[132] Phillips J.C., Hardy D.J., Maia J.D.C., Stone J.E., Ribeiro J.V., Bernardi R.C., Buch R., Fiorin G., Henin J., Jiang W. (2020). Scalable molecular dynamics on CPU and GPU architectures with NAMD. J. Chem. Phys..

[133] Seritan S., Bannwarth C., Fales B.S., Hohenstein E.G., Isborn C.M., Kokkila-Schumacher S.I.L., Li X., Liu F., Luehr N., Snyder J.W. (2020). TeraChem: A graphical processing unit—Accelerated electronic structure package for large-scale ab initio molecular dynamics. Wiley Interdiscip. Rev. Comput. Mol. Sci..

[134] Humphrey W., Dalke A., Schulten K. (1996). VMD: Visual molecular dynamics. J. Mol. Graph..

[135] Vanommeslaeghe K., Hatcher E., Acharya C., Kundu S., Zhong S., Shim J., Darian E., Guvench O., Lopes P., Vorobyov I. (2010). CHARMM general force field: A force field for drug-like molecules compatible with the CHARMM all-atom additive biological force fields. J. Comput. Chem..

[136] Vermaas J.V., Crowley M.F., Beckham G.T. (2020). Molecular Lignin Solubility and Structure in Organic Solvents. ACS Sustain. Chem. Eng..

[137] Sushko I., Novotarskyi S., Korner R., Pandey A.K., Rupp M., Teetz W., Brandmaier S., Abdelaziz A., Prokopenko V.V., Tanchuk V.Y. (2011). Online chemical modeling environment (OCHEM): Web platform for data storage, model development and publishing of chemical information. J. Comput. Aided. Mol. Des..

[138] Radchenko E.V., Dyabina A.S., Palyulin V.A., Zefirov N.S. (2016). Prediction of human intestinal absorption of drug compounds. Russ. Chem. Bull..

[139] Dyabina A.S., Radchenko E.V., Palyulin V.A., Zefirov N.S. (2016). Prediction of blood-brain barrier permeability of organic compounds. Dokl. Biochem. Biophys..

[140] Radchenko E.V., Dyabina A.S., Palyulin V.A. (2020). Towards Deep Neural Network Models for the Prediction of the Blood-Brain Barrier Permeability for Diverse Organic Compounds. Molecules.

[141] Radchenko E.V., Rulev Y.A., Safanyaev A.Y., Palyulin V.A., Zefirov N.S. (2017). Computer-aided estimation of the hERG-mediated cardiotoxicity risk of potential drug components. Dokl. Biochem. Biophys..

[142] ADMET Prediction Service. http://qsar.chem.msu.ru/admet/.

[143] Bickerton G.R., Paolini G.V., Besnard J., Muresan S., Hopkins A.L. (2012). Quantifying the chemical beauty of drugs. Nat. Chem..

[144] RDKit: Open-Source Cheminformatics Software. http://www.rdkit.org.

[145] Charni-Natan M., Goldstein I. (2020). Protocol for Primary Mouse Hepatocyte Isolation. STAR Protoc..

[146] Voevodin V.V., Antonov A.S., Nikitenko D.A., Shvets P.A., Sobolev S.I., Sidorov I.Y., Stefanov K.S., Voevodin V.V., Zhumatiy S.A. (2019). Supercomputer Lomonosov-2: Large Scale, Deep Monitoring and Fine Analytics for the User Community. Supercomput. Front. Innov..

