# Supplementary material for: Conjugates of Tacrine and Salicylic Acid Derivatives as New Promising Multitarget Agents for Alzheimer’s Disease
Source: Int J Mol Sci. 2023 Jan 24;24(3):2285. doi: 10.3390/ijms24032285 (PMC9916969; doi:10.3390/ijms24032285)
Supplement: Supplementary file 1 [file ijms-24-02285-s001.zip › ijms-2154456-supplementary.pdf]

Conjugates of Tacrine and Salicylic Acid Derivatives as New Promising Multitarget Agents for Alzheimer's Disease

Galina F. Makhaeva [1], Nadezhda V. Kovaleva [1], Elena V. Rudakova [1], Natalia P. Boltneva [1], Maria V. Grishchenko [2], Sofya V. Lushchekina [1,3], Tatiana Y. Astakhova [3], Olga G. Serebryakova [1], Elena N. Timokhina [3], Ekaterina F. Zhilina [2], Evgeny V. Shchegolkov [2], Mariya V. Ulitko [4], Eugene V. Radchenko [1,5], Vladimir A. Palyulin [1,5], Yanina V. Burgart [2], Victor I. Saloutin [2], Sergey O. Bachurin [1] and Rudy J. Richardson [6,7,8,9,*]

1 Institute of Physiologically Active Compounds at Federal Research Center of Problems of Chemical Physics and Medicinal Chemistry, Russian Academy of Sciences, Chernogolovka 142432, Russia
2 Postovsky Institute of Organic Synthesis, Urals Branch of Russian Academy of Sciences, Yekaterinburg 620990, Russia
3 Emanuel Institute of Biochemical Physics Russian Academy of Sciences, Moscow 119334, Russia
4 Institute of Natural Sciences and Mathematics of the Ural Federal University Named after the First President of Russia B. N. Yeltsin, Ekaterinburg 620083, Russia
5 Department of Chemistry, Lomonosov Moscow State University, Moscow 119991, Russia
6 Department of Environmental Health Sciences, University of Michigan, Ann Arbor, MI 48109, USA
7 Department of Neurology, University of Michigan, Ann Arbor, MI 48109, USA
8 Center of Computational Medicine and Bioinformatics, University of Michigan, Ann Arbor, MI 48109, USA
9 Michigan Institute for Computational Discovery and Engineering, University of Michigan, Ann Arbor, MI 48109, USA
* Correspondence: rjrich@umich.edu; Tel.: +1-734-936-0769

Table of contents

Figure S1. IR spectrum of compound **6a** 4
Figure S2. ^{1}H NMR spectrum of compound **6a**....................5
Figure S3. ^{13}C NMR spectrum of compound **6a**....................6
Figure S4. IR spectrum of compound **6b** 8
Figure S5. ^{1}H NMR spectrum of compound **6b** 9
Figure S6. ^{13}C NMR spectrum of compound **6b** 10
Figure S7. IR spectrum of compound **6c** 12
Figure S8. ^{1}H NMR spectrum of compound **6c**....................13
Figure S9. ^{13}C NMR spectrum of compound **6c**....................14
Figure S10. HRMS spectrum of compound **6c**....................15
Figure S11. IR spectrum of compound **7a** 16
Figure S12. ^{1}H NMR spectrum of compound **7a** 17
Figure S13. ^{13}C NMR spectrum of compound **7a**....................18
Figure S14. ^{19}F NMR spectrum of compound **7a**....................19
Figure S15. HMRS spectrum of compound **7a** 20
Figure S16. IR spectrum of compound **7b**....................21
Figure S17. ^{1}H NMR spectrum of compound **7b** 22

Figure S18. ^{13}C NMR spectrum of compound **7b** 23
Figure S19. ^{19}F NMR spectrum of compound **7b** 24
Figure S20. HRMS spectrum of compound **7b** 25
Figure S21. IR spectrum of compound **7c** 26
Figure S22. ^{1}H NMR spectrum of compound **7c** 27
Figure S23. ^{13}C NMR spectrum of compound **7c** 28
Figure S24. ^{19}F NMR spectrum of compound **7c** 29
Figure S25. HMRS spectrum of compound **7c** 30
Figure S26. IR spectrum of compound **8a** 31
Figure S26. ^{1}H NMR spectrum of compound **8a** 32
Figure S27. ^{13}C NMR spectrum of compound **8a** 33
Figure S28. ^{19}F NMR spectrum of compound **8a** 34
Figure S29. HRMS spectrum of compound **8a** 35
Figure S30. IR spectrum of compound **8b** 36
Figure S31. ^{1}H NMR spectrum of compound **8b** 37
Figure S33. ^{13}C NMR spectrum of compound **8b** 38
Figure S34. ^{19}F NMR spectrum of compound **8b** 39
Figure S35. HRMS spectrum of compound **8b** 40
Figure S36. IR spectrum of compound **8c** 41
Figure S37. ^{1}H NMR spectrum of compound **8c** 42
Figure S38. ^{13}C NMR spectrum of compound **8c** 43
Figure S39. ^{19}F NMR spectrum of compound **8c** 44
Figure S40. HRMS spectrum of compound **8c** 45
Figure S41. IR spectrum of compound **10a** 46
Figure S42. ^{1}H NMR spectrum of compound **10a** 47
Figure S43. ^{13}C NMR spectrum of compound **10a** 48
Figure S44. HRMS spectrum of compound **10a** 49
Figure S45. IR spectrum of compound **10b** 50
Figure S46. ^{1}H NMR spectrum of compound **10b** 51
Figure S47. ^{13}C NMR spectrum of compound **10b** 52
Figure S48. HMRS spectrum of compound **10b** 53
Figure S49. IR spectrum of compound **10c** 54
Figure S50. ^{1}H NMR spectrum of compound **10c** 55
Figure S51. ^{13}C NMR spectrum of compound **10c** 56
Figure S52. HRMS spectrum of compound **10c** 57
Figure S53. Absorption spectra of: compound **6b**, Cu^{2+} ions solution, a sum of **6b** and Cu^{2+}, their mixture, and the shift of the spectra caused by the formation of a complex. 58
Figure S54. UV spectra of compound **7b** and mixture of **7b** with Cu(II), Fe(II) and Zn(II) ions. 58

Figure S55. UV spectra of compound **8b** and mixture of **8b** with Cu(II), Fe(II) and Zn(II) ions. 59

Figure S56. UV spectra of compound **10b** and mixture of **10b** with Cu(II), Fe(II) and Zn(II) ions. 59

Figure S57. (a) UV absorption spectra of **6b** (20 μM) in ethanol after addition of increasing concentrations of $CuCl_2$ (2-34 μM). **(b)** the differential spectra due to 6b-Cu^{2+} complex formation obtained by numerical subtraction from the spectra of the mixture of the spectra of the Cu^{2+} alone and 6b alone at the corresponding concentrations. 60

Figure S58. **(a)** UV absorption spectra of **10b** (20 μM) in ethanol after addition of increasing concentrations of $CuCl_2$ (2-34 μM). **(b)** the differential spectra due to **10b**-Cu^{2+} complex formation obtained by numerical subtraction from the spectra of the mixture of the spectra of the Cu^{2+} alone and **10b** alone at the corresponding concentrations. 60

Figure S59 . IC_{50} values for AChE inhibition by compounds **5c**,**6c,7c,8c and 10c** (MEAN ± SEM, n = 3) 61

Figure S60. IC_{50} values for BChE inhibition by compounds **5c**,**6c,7c,8c and 10c** (MEAN ± SEM, n = 3) 62

Figure S61. Lineweaver-Burk double-reciprocal plots of steady state inhibition of AChE by compounds **5c**,**6c,7c,8c**. Each plot indicates mixed-type inhibition. 63

Figure S62. Lineweaver-Burk double-reciprocal plots of steady state inhibition of BChE by compounds **5c**,**6c,7c,8c**. Each plot indicates mixed-type inhibition……………………………………………………………………………….64

Table S1. pKa values and charges of the investigated compounds…………………………………………………………65

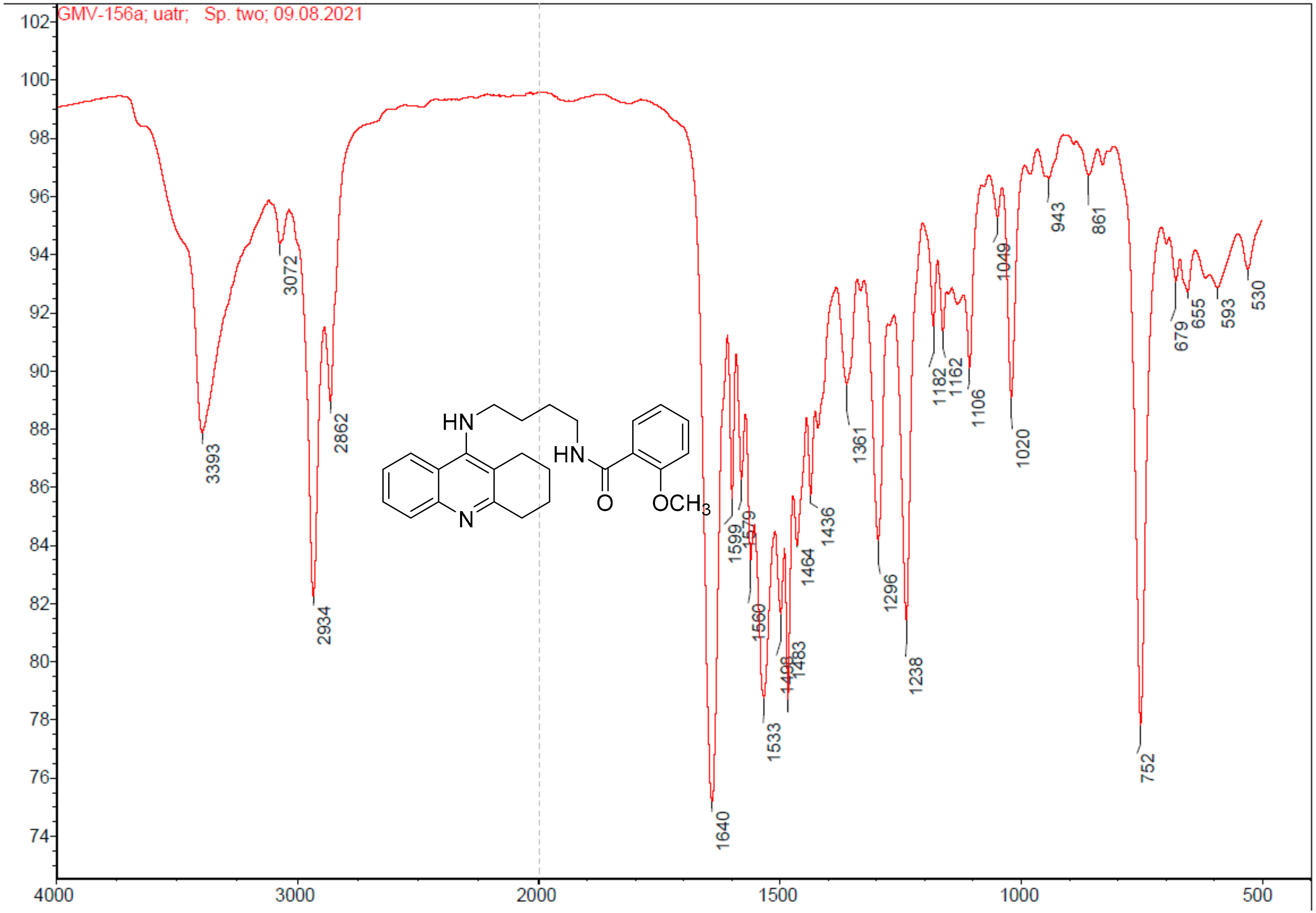

Figure S1. IR spectrum of compound **6a**

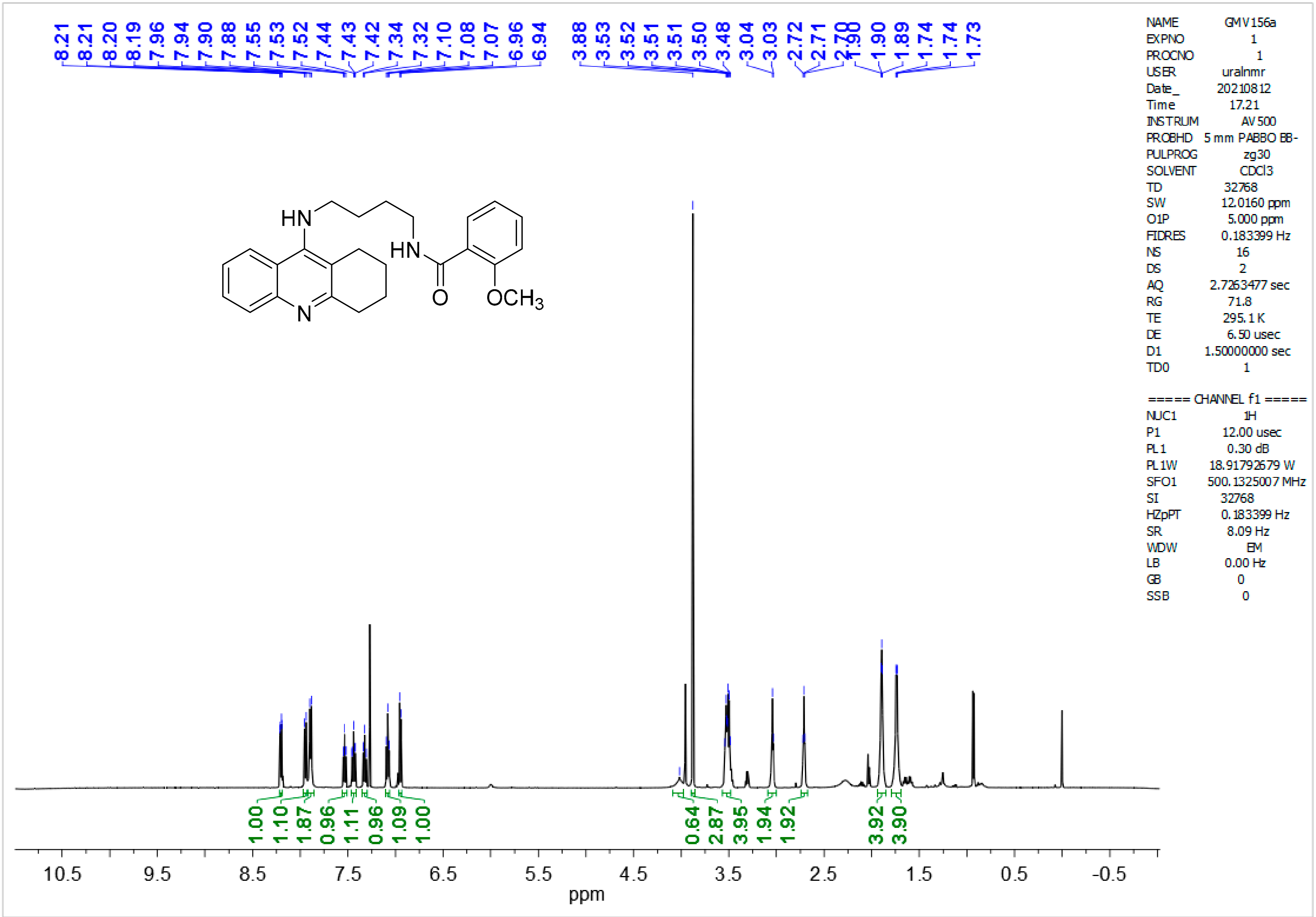

Figure S2. ¹H NMR spectrum of compound **6a**

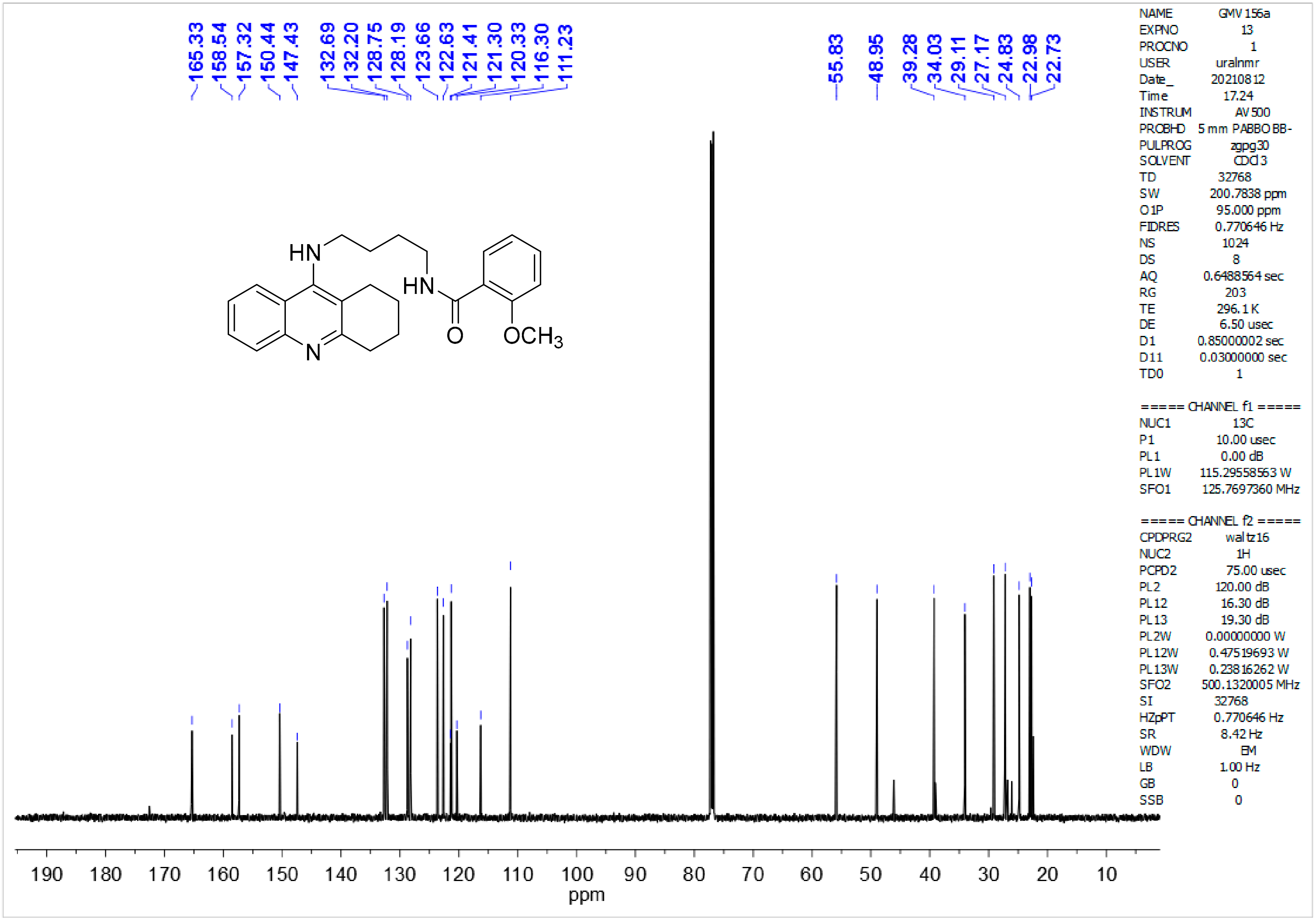

Figure S3. ^{13}C NMR spectrum of compound **6a**

Compound Spectrum SmartFormula Report

Analysis Info

Acquisition Date	9/22/2021 1:16:38 PM
Analysis Name	D:\Data\ING21\GMV-156a.22i-C.EP180.6219_22i1315.d
Method	EP180UI21HPC50-1600_500-3500-0.4-4-200_1f2002f200hrf 70ie5lm70ce10pps6crf300-1200tt40-110_F3x1_Segm1.m
Operator	admin
Sample Name	
Instrument	maXis impact 1819696.00172
Comment	22/09/2022: +Bckgnd: 118.09, 322.05, 622.03, 922.01, 1221.99, 1521.97, 1821.95, 2121.93, 2421.91, 2721.89 (G1969-85000; +/-299.981 HPC); other intense peaks (>2*e4): 102.13 (NEt3); 132.91 (*2-PrOH); 391.28&413.26 (DOP); 86.10, 113.13, 140.07, 149.02, 158.96, 167.03, 187.07. 194.10, 203.14, 207.17, 209.19, 214.25, 217.10, 223.21, 227.23, 237.22, 245.19, 249.22, 251.24, 255.27, 259.20, 263.23, 265.25, 273.22, 279.16, 291.27, 293.28, 304.30, 307.30, 321.31, 326.38, 332.33, 335.33, 349.35, 413.27, 1259.95, 1307.08, 1559.93: background (prev. analyzed samples and impurities); 188.09 (#6216); 588.32 (#6218)

Acquisition Parameter

Source Type	ESI	Ion Polarity	Positive	Set Nebulizer	0.4 Bar
Focus	Active	Set Capillary	3500 V	Set Dry Heater	200 °C
Scan Begin	50 m/z	Set End Plate Offset	-500 V	Set Dry Gas	4.0 l/min
Scan End	1600 m/z	Set Charging Voltage	2000 V	Set Divert Valve	Source
		Set Corona	0 nA	Set APCI Heater	0 °C

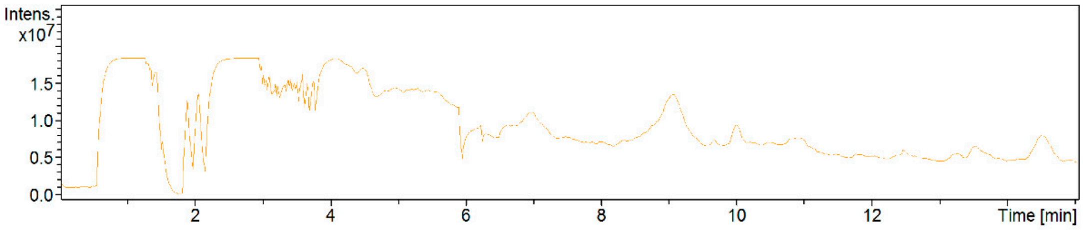

+MS, 6.1-6.4min #353-367 (EQ Int.)

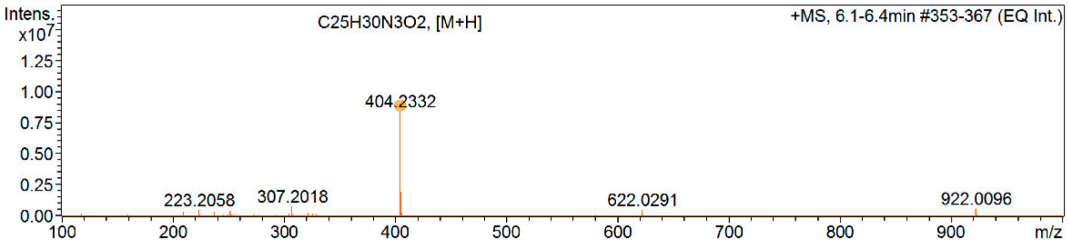

Meas. m/z	#	Ion Formula	m/z	err [ppm]	mSigma	# mSigma	Score	rdb	e⁻ Conf	N-Rule
404.2332	1	C20H30N5O4	404.2292	-9.9	4.2	1	37.77	8.5	even	ok
	2	C21H26N9	404.2306	-6.6	16.2	2	91.58	13.5	even	ok
	3	C25H30N3O2	404.2333	0.1	30.2	3	100.00	12.5	even	ok

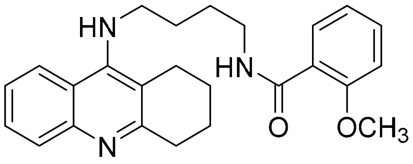

GMV-156a.22i-C.EP180.6219_22i1315.d

Bruker Compass DataAnalysis 4.2 printed: 9/22/2021 2:11:25 PM by: admin Page 1 of 1

Institute of Organic Synthesis UB RAS
22 S.Kovalevskoy, 20 Akademicheskaya str, Yekaterinburg, Russian Federation
Phone: +7 (343) 362-34-56

BRUKER

Figure S4. HMRS spectrum of compound **6a**

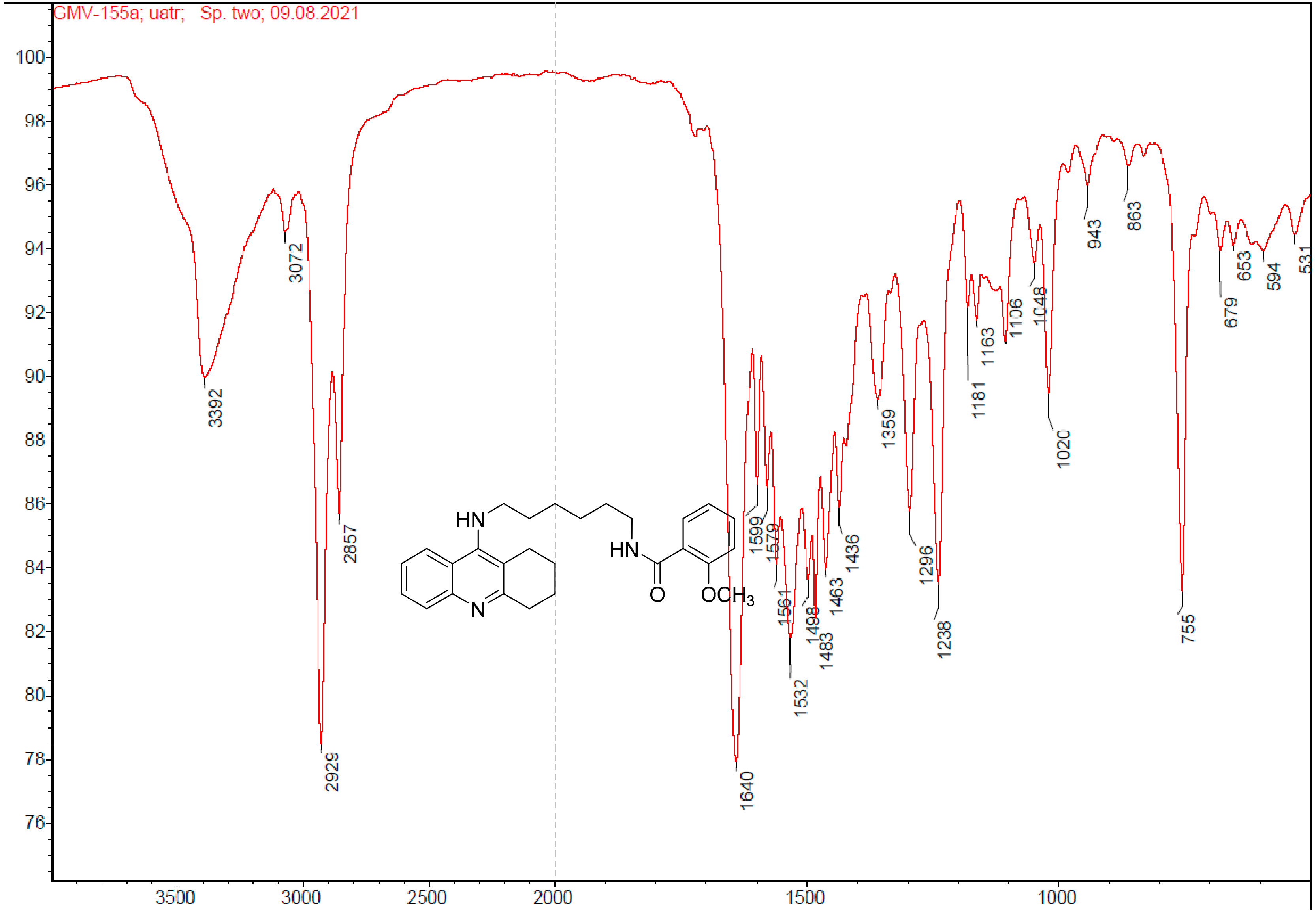

Figure S4. IR spectrum of compound **6b**

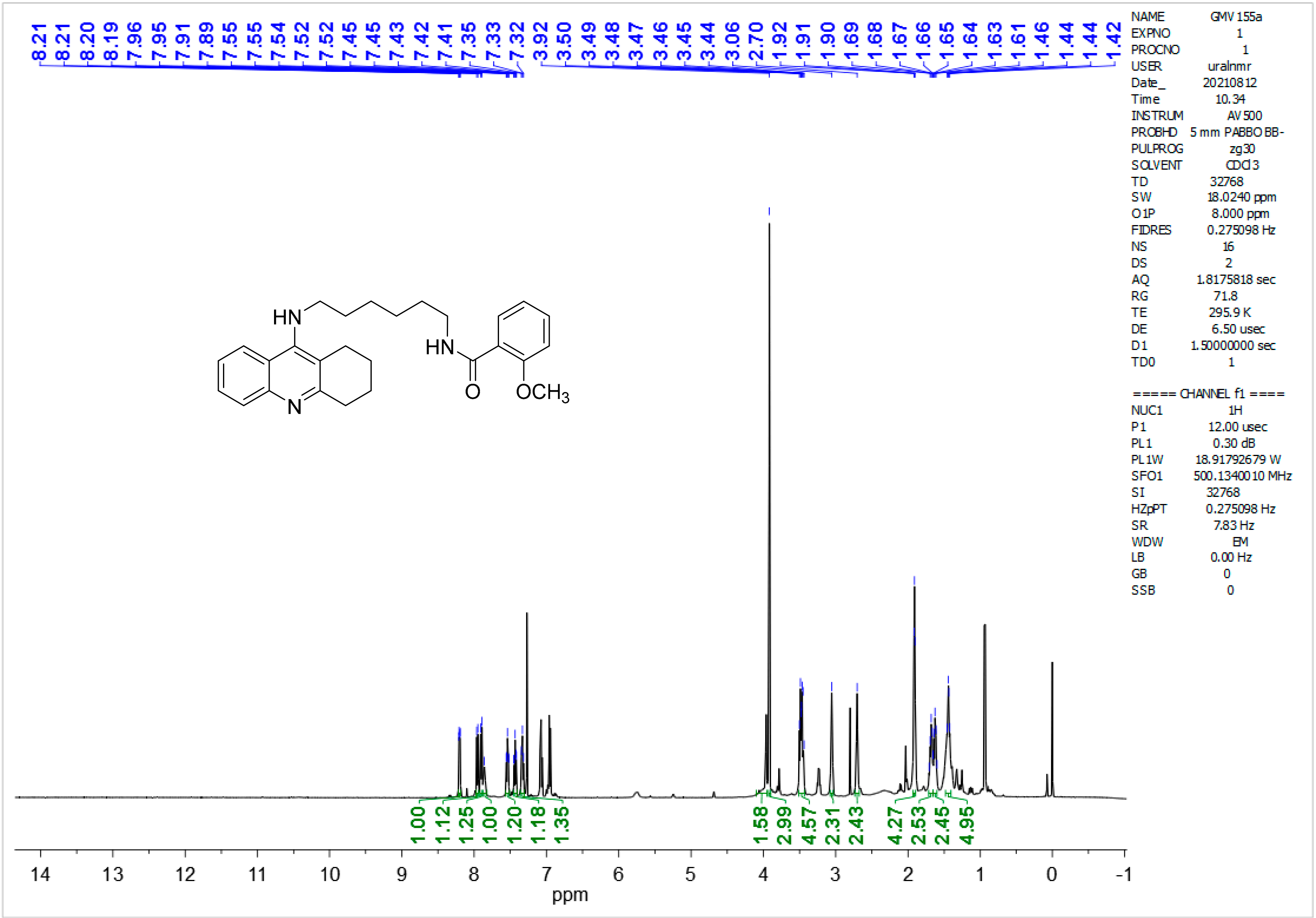

Figure S5. ^{1}H NMR spectrum of compound **6b**

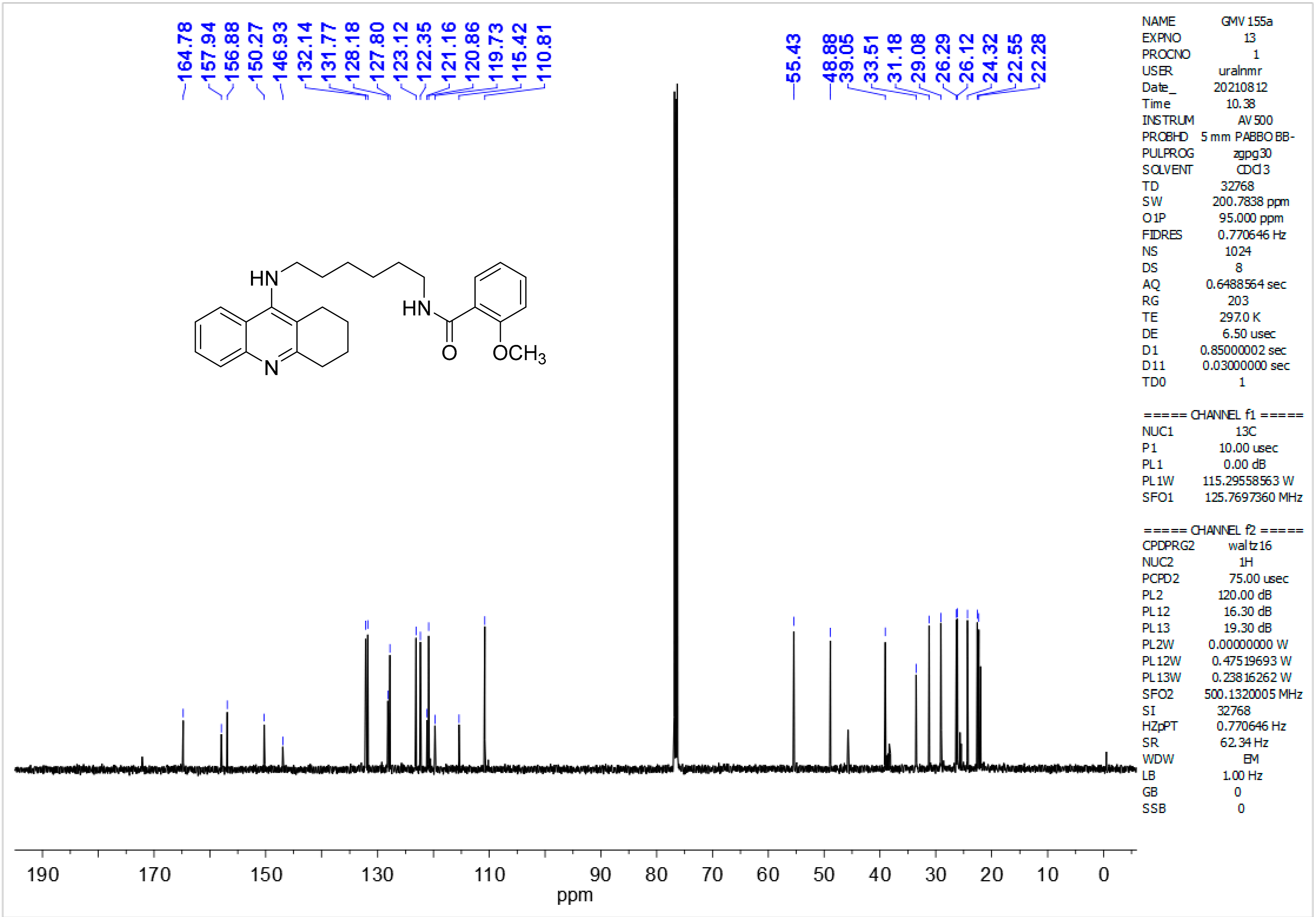

Figure S6. ^{13}C NMR spectrum of compound **6b**

Compound Spectrum SmartFormula Report

Analysis Info | Acquisition Date 9/23/2021 10:10:46 AM

Analysis Name D:\Data\ING21\GMV-155a.22i-C.EP180.6222_23i1015.d
Method EP180UI21HPC50-1600_500-3500-0.4-4-200_1f2002f200hrf70ie5lm70ce10pps6crf300-1200tt40-110_F3x1_Segm1.m
Sample Name | Instrument maXis impact
Comment 2/09/2022: +Bckgnd: 118.09, 322.05, 622.03, 922.01, 1221.99, 1521.97, 1821.95, 2121.93, 2421.91, 2721.89 (G1969-85000; +/-299.981 HPC); other intense peaks (>2*e4): 102.13 (NEt3); 132.91 (*2-PrOH); 391.28&413.26 (DOP); 86.10, 113.13, 140.07, 149.02, 158.96, 167.03, 187.07. 194.10, 203.14, 207.17, 209.19, 214.25, 217.10, 223.21, 227.23, 237.22, 245.19, 249.22, 251.24, 255.27, 259.20, 263.23, 265.25, 273.22, 279.16, 291.27, 293.28, 304.30, 307.30, 321.31, 326.38, 332.33, 335.33, 349.35, 413.27, 1259.95, 1307.08, 1559.93: background (prev. analyzed samples and impurities); 188.09 (#6216); 588.32 (#6218); 404.23 (#6219); 376.20 (#6220); 440.21 (#6221)

Acquisition Parameter 1819696.00172

Source Type	ESI	Ion Polarity	Positive	Set Nebulizer	0.4 Bar
Focus	Active	Set Capillary	3500 V	Set Dry Heater	200 °C
Scan Begin	50 m/z	Set End Plate Offset	-500 V	Set Dry Gas	4.0 l/min
Scan End	1600 m/z	Set Charging Voltage	2000 V	Set Divert Valve	Source
		Set Corona	0 nA	Set APCI Heater	0 °C

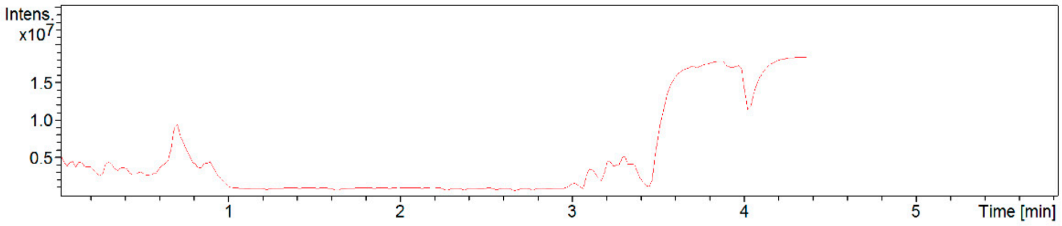

+MS, 3.7-4.0min #210-227

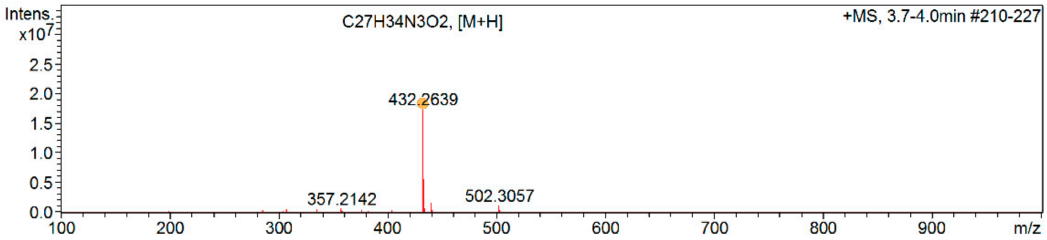

Meas. m/z	#	Ion Formula	m/z	err [ppm]	mSigma	# mSigma	Score	rdb	e⁻ Conf	N-Rule
432.2639	1	C27H34N3O2	432.2646	1.5	10.9	1	100.00	12.5	even	ok
	2	C23H30N9	432.2619	-4.7	22.7	2	33.17	13.5	even	ok

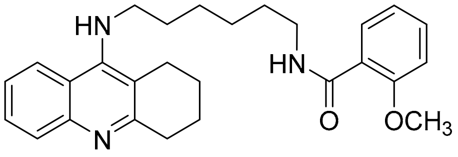

GMV-155a.22i-C.EP180.6222_23i1015.d
Bruker Compass DataAnalysis 4.2 printed: 9/23/2021 10:18:25 AM Page 1 of 1

Figure S7. HMRS spectrum of compound **6b**

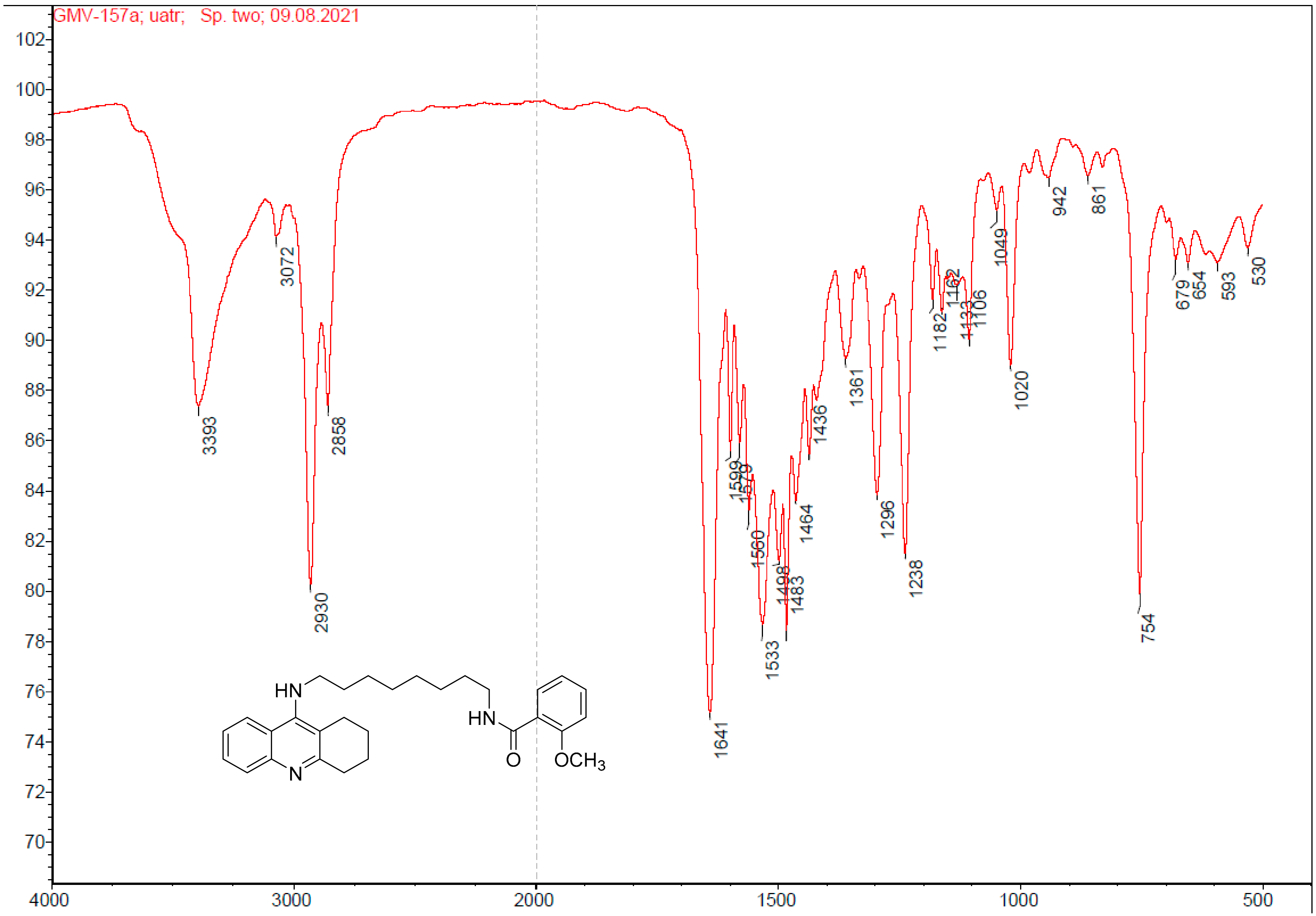

Figure S7. IR spectrum of compound **6c**

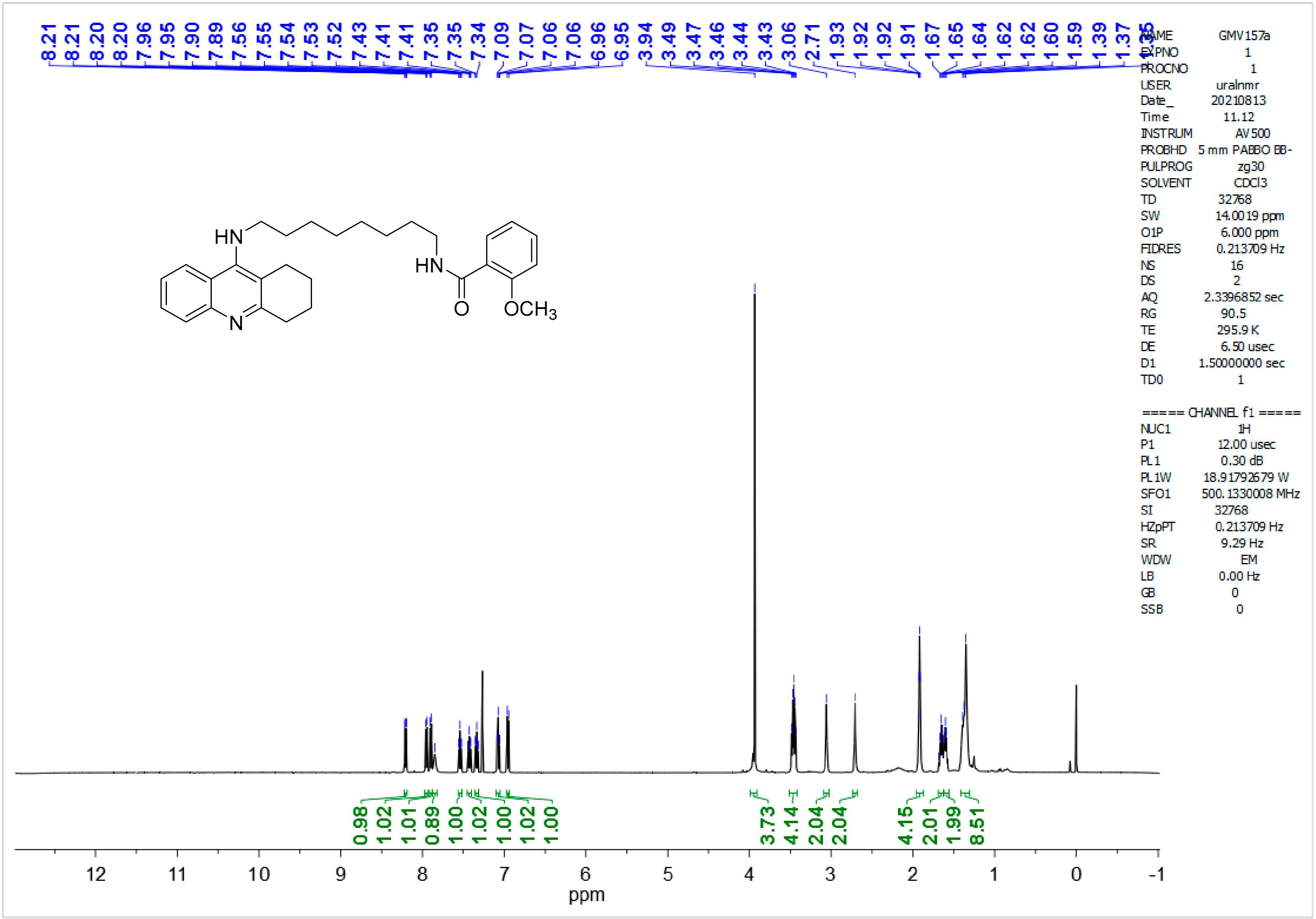

Figure S8. ^{1}H NMR spectrum of compound **6c**

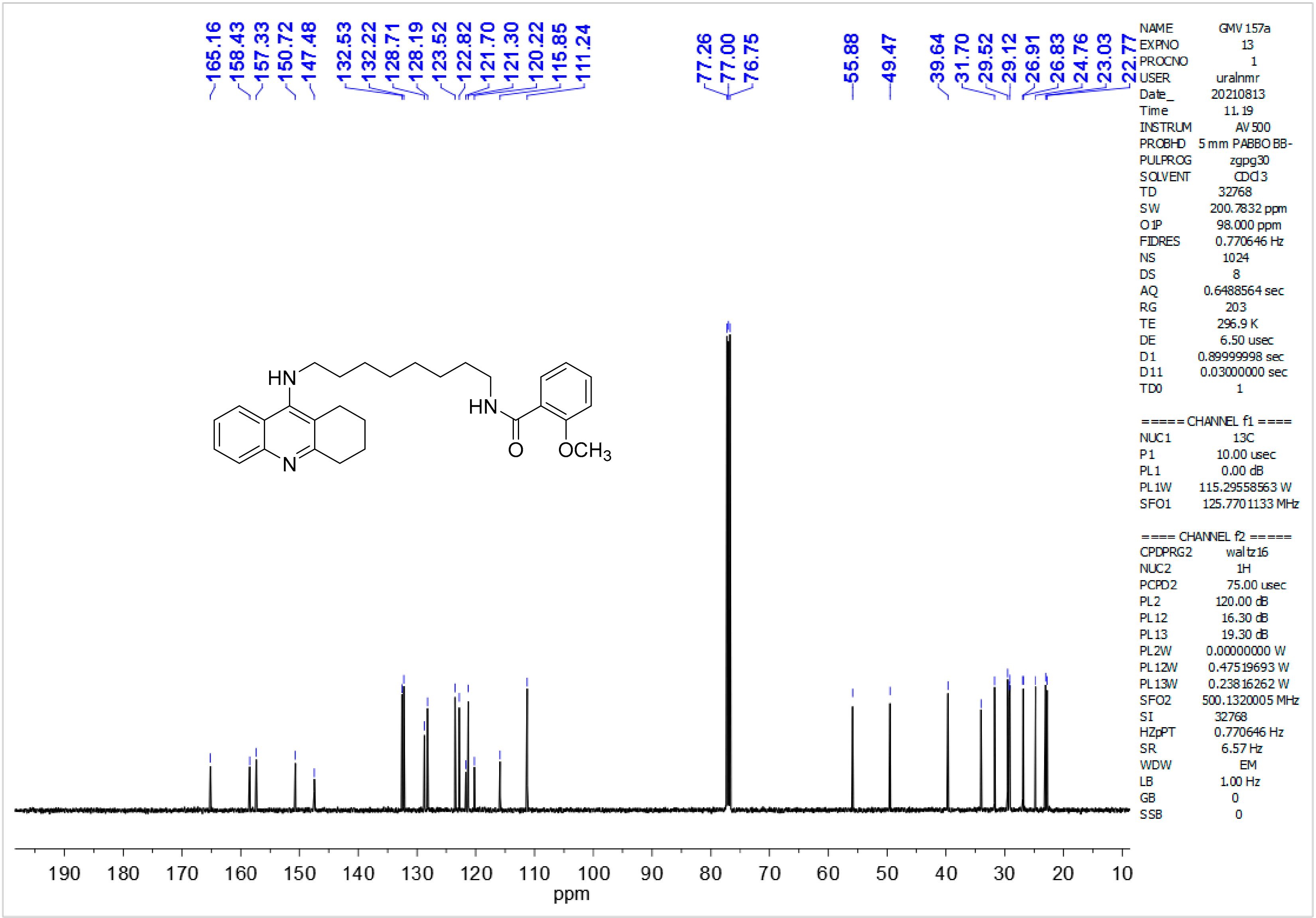

Figure S9. ^{13}C NMR spectrum of compound **6c**

Compound Spectrum SmartFormula Report

Analysis Info — Acquisition Date 9/23/2021 11:25:07 AM

Analysis Name D:\Data\ING21\GMV-157a.22i-C.EP180.6224_23i1100.d
Method EP180UI21HPC50-1600_500-3500-0.4-4-200_1f2002f200hrf70ie5lm70ce10pps6crf300-1200tt40-110_F3x1_Segm1.m
Sample Name — Instrument maXis impact
Comment 2/09/2022: +Bckgnd: 118.09, 322.05, 622.03, 922.01, 1221.99, 1521.97, 1821.95, 2121.93, 2421.91, 2721.89 (G1969-85000; +/-299.981 HPC); other intense peaks (>2*e4): 102.13 (NEt3); 132.91 (*2-PrOH); 391.28&413.26 (DOP); 86.10, 113.13, 140.07, 149.02, 158.96, 167.03, 187.07. 194.10, 203.14, 207.17, 209.19, 214.25, 217.10, 223.21, 227.23, 237.22, 245.19, 249.22, 251.24, 255.27, 259.20, 263.23, 265.25, 273.22, 279.16, 291.27, 293.28, 304.30, 307.30, 321.31, 326.38, 332.33, 335.33, 349.35, 413.27, 1259.95, 1307.08, 1559.93: background (prev. analyzed samples and impurities); 188.09 (#6216); 588.32 (#6218); 404.23 (#6219); 376.20 (#6220); 440.21 (#6221)

Acquisition Parameter — 1819696.00172

Source Type	ESI	Ion Polarity	Positive	Set Nebulizer	0.4 Bar
Focus	Active	Set Capillary	3500 V	Set Dry Heater	200 °C
Scan Begin	50 m/z	Set End Plate Offset	-500 V	Set Dry Gas	4.0 l/min
Scan End	1600 m/z	Set Charging Voltage	2000 V	Set Divert Valve	Source
		Set Corona	0 nA	Set APCI Heater	0 °C

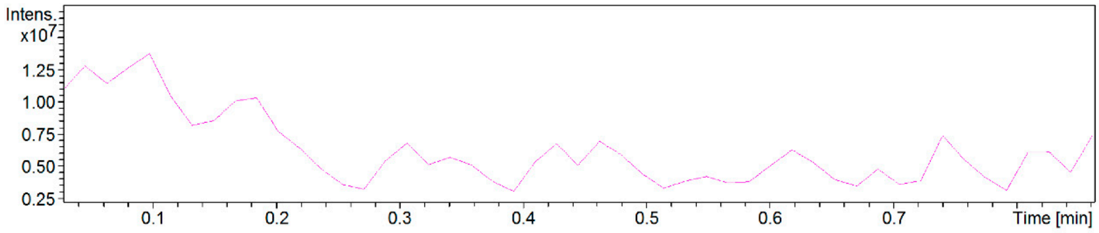

+MS, 0.4-0.8min #20-47

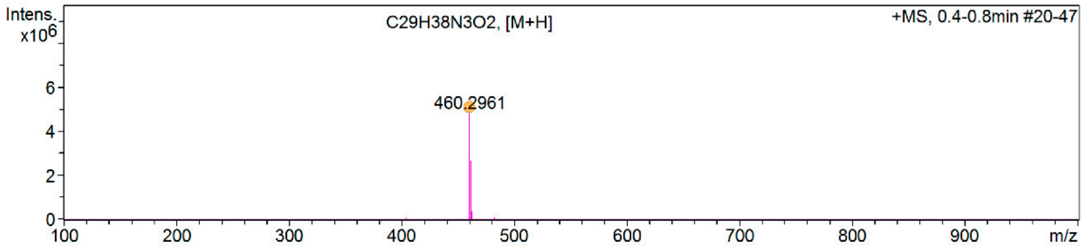

Meas. m/z	#	Ion Formula	m/z	err [ppm]	mSigma	# mSigma	Score	rdb	e⁻ Conf	N-Rule
460.2961	1	C29H38N3O2	460.2959	-0.5	130.0	1	100.00	12.5	even	ok
	2	C17H42N5O9	460.2977	3.5	199.6	2	0.33	-0.5	even	ok
	3	C14H34N15O3	460.2964	0.6	200.1	3	0.67	5.5	even	ok
	4	C13H38N11O7	460.2950	-2.4	213.1	4	0.14	0.5	even	ok

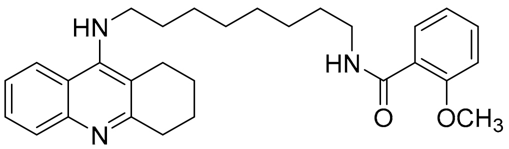

GMV-157a.22i-C.EP180.6224_23i1100.d
Bruker Compass DataAnalysis 4.2 printed: 9/23/2021 11:27:05 AM Page 1 of 1

Figure S10. HRMS spectrum of compound **6c**

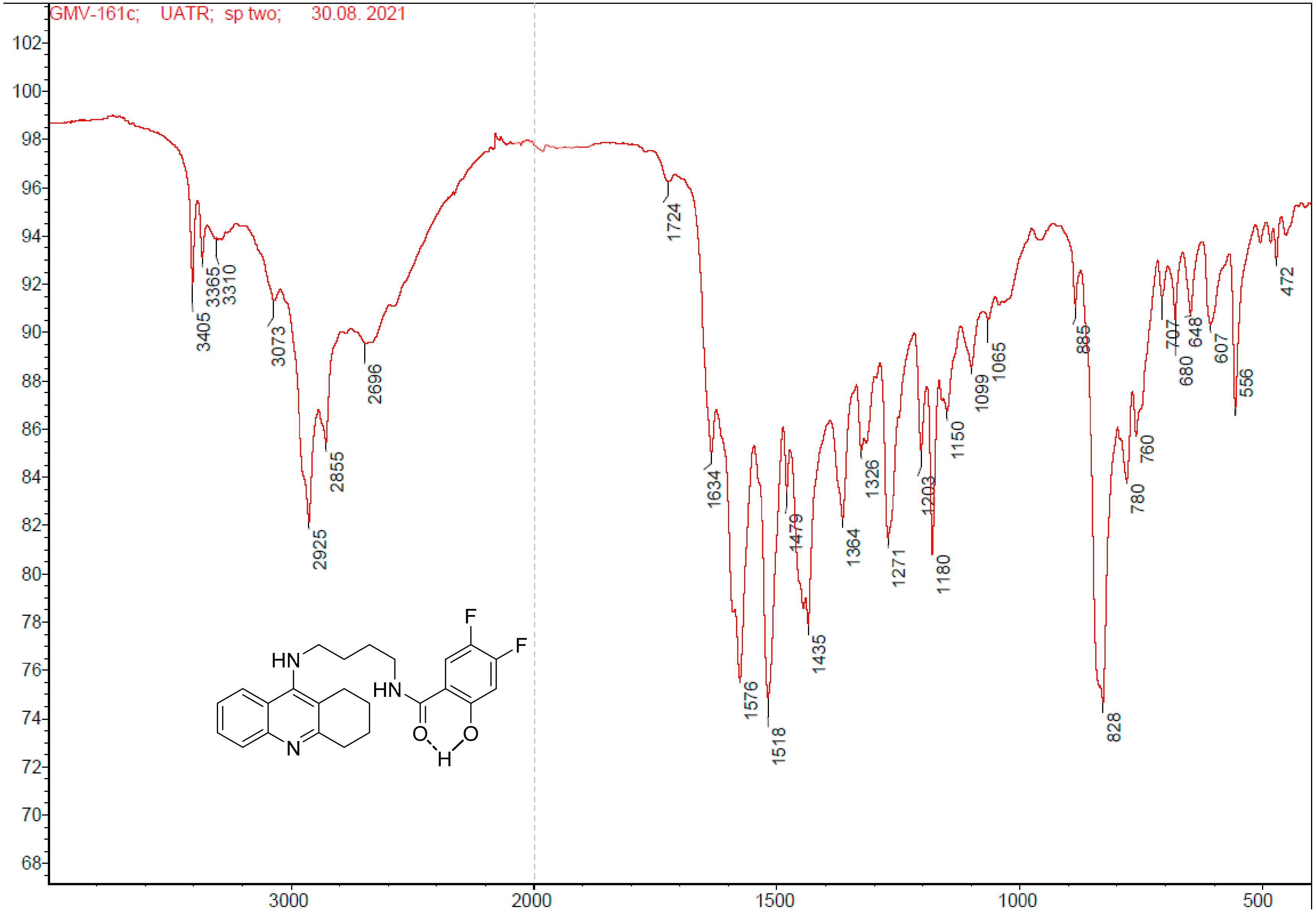

Figure S11. IR spectrum of compound **7a**

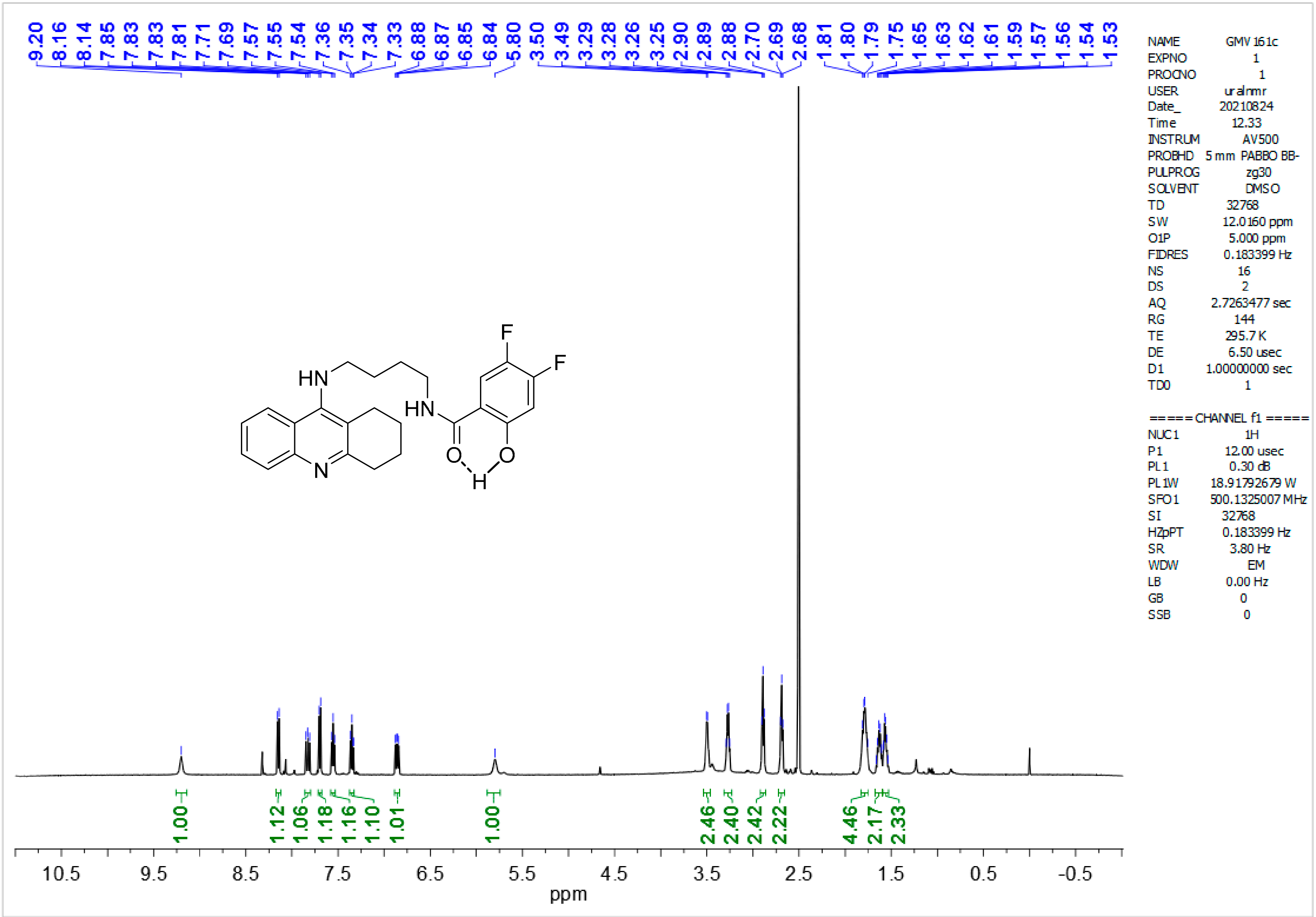

Figure S12. ^{1}H NMR spectrum of compound **7a**

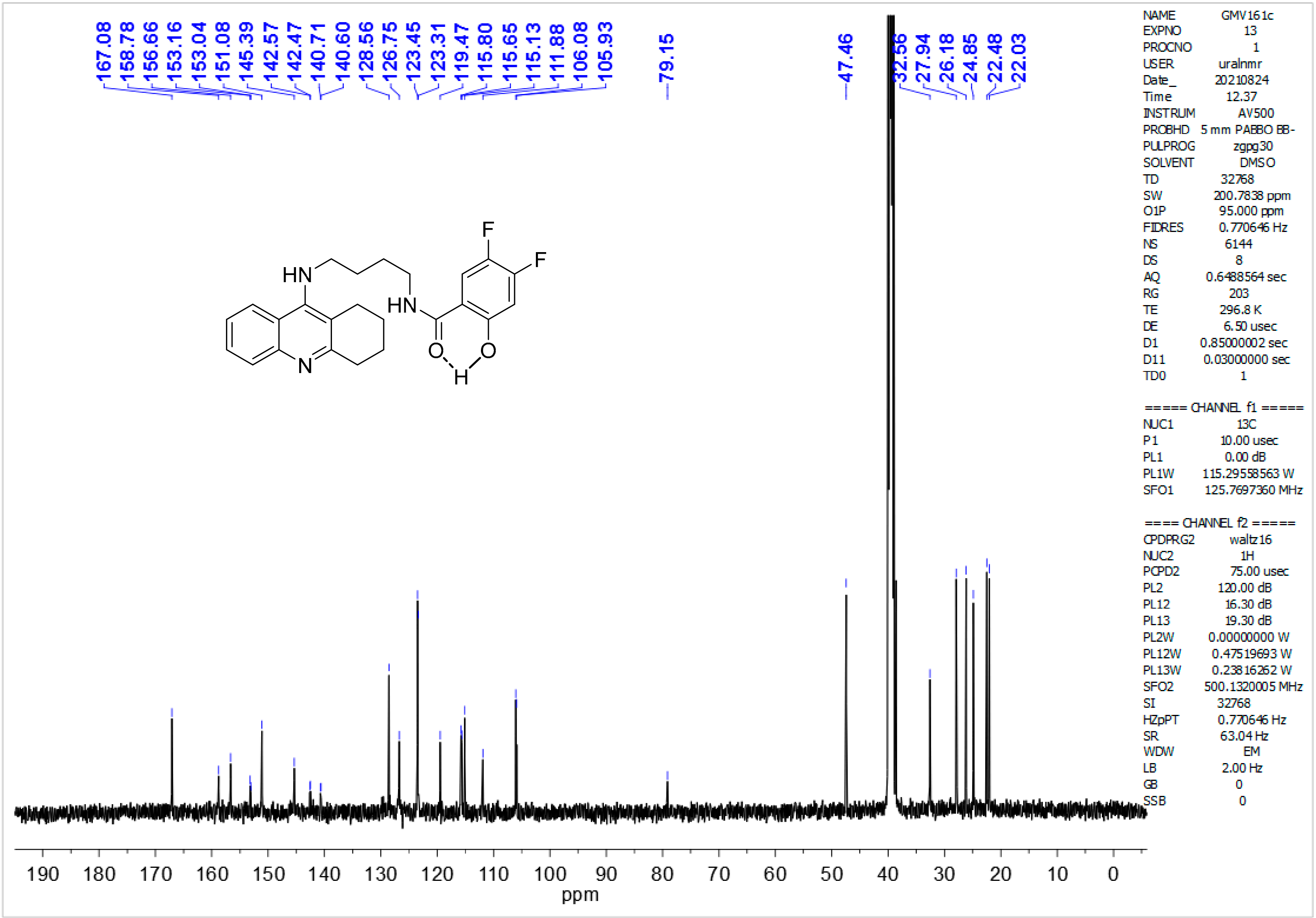

Figure S13. ^{13}C NMR spectrum of compound **7a**

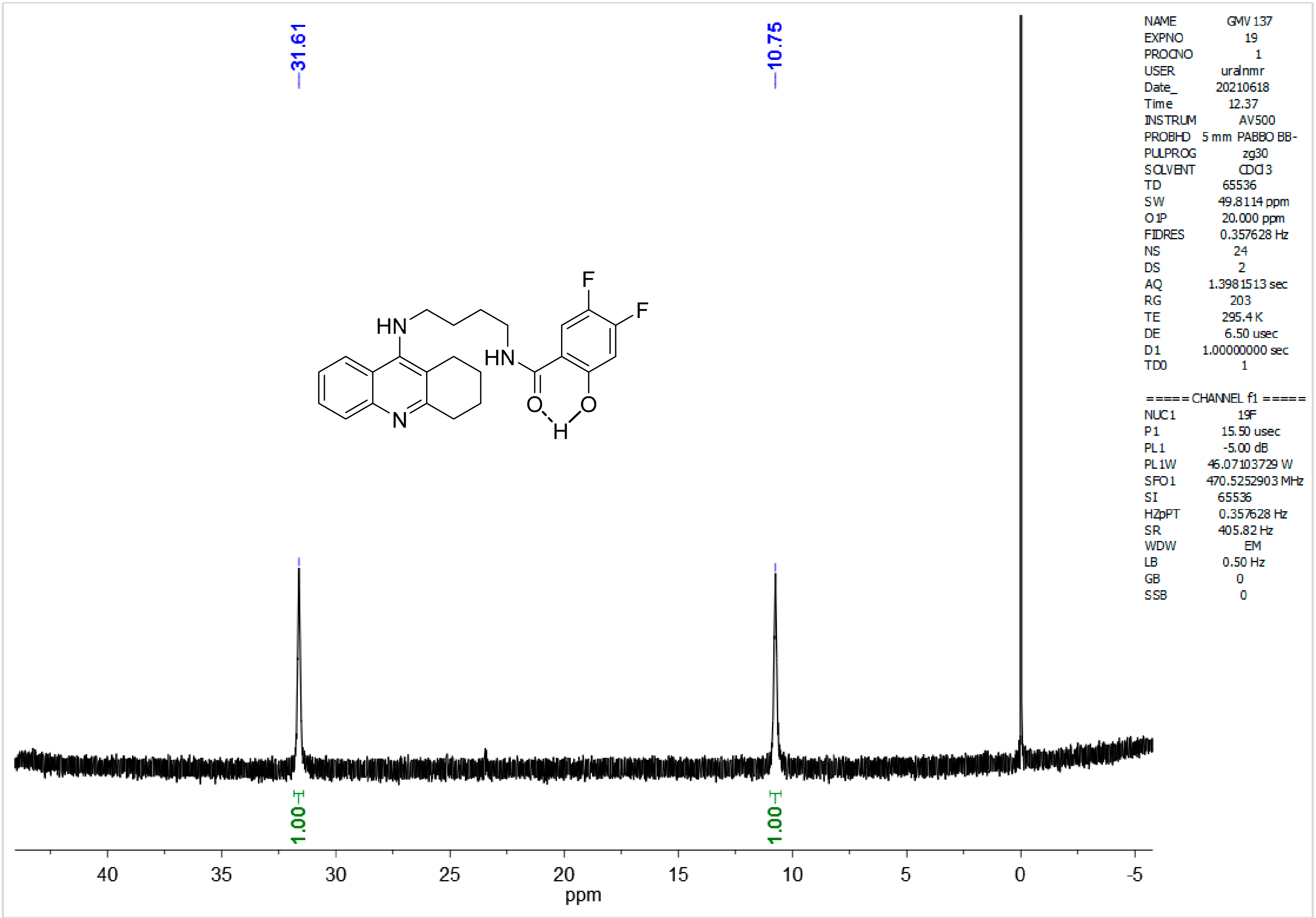

Figure S14. ^{19}F NMR spectrum of compound **7a**

Compound Spectrum SmartFormula Report

Analysis Info

Analysis Name	D:\Data\ING21\GMV-161c.23i-C.EP180.6240_23i1300.d	Acquisition Date	9/23/2021 1:00:30 PM
Method	EP180UI21HPC50-1600_500-3500-0.4-4-200_1f2002f200hrf 70ie5lm70ce10pps6crf300-1200tt40-110_F3x1_Segm1.m	Operator	admin
Sample Name		Instrument	maXis impact 1819696.00172

Comment: 23/09/2022: +Bckgnd: 118.09, 322.05, 622.03, 922.01, 1221.99, 1521.97, 1821.95, 2121.93, 2421.91, 2721.89 (G1969-85000; +/-299.981 HPC); other intense peaks (>2*e4): 102.13 (NEt3); 132.91 (*2-PrOH); 391.28&413.26 (DOP); 86.10, 113.13, 140.07, 149.02, 158.96, 167.03, 187.07. 194.10, 203.14, 207.17, 209.19, 214.25, 217.10, 223.21, 227.23, 237.22, 245.19, 249.22, 251.24, 255.27, 259.20, 263.23, 265.25, 273.22, 279.16, 291.27, 293.28, 304.30, 307.30, 321.31, 326.38, 332.33, 335.33, 349.35, 413.27, 1259.95, 1307.08, 1559.93: background (prev. analyzed samples and impurities); 188.09 (#6216); 588.32 (#6218); 404.23 (#6219); 376.20 (#6220); 440.21 (#6221); 460.30 (#6224); 482.26 (#6239)

Acquisition Parameter

Source Type	ESI	Ion Polarity	Positive	Set Nebulizer	0.4 Bar
Focus	Active	Set Capillary	3500 V	Set Dry Heater	200 °C
Scan Begin	50 m/z	Set End Plate Offset	-500 V	Set Dry Gas	4.0 l/min
Scan End	1600 m/z	Set Charging Voltage	2000 V	Set Divert Valve	Source
		Set Corona	0 nA	Set APCI Heater	0 °C

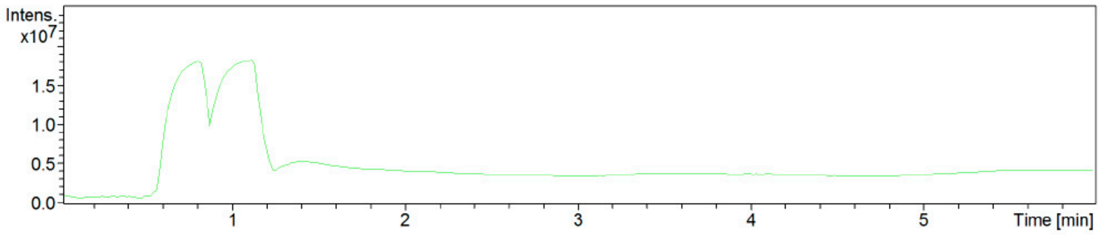

+MS, 0.7-0.9min #42-49

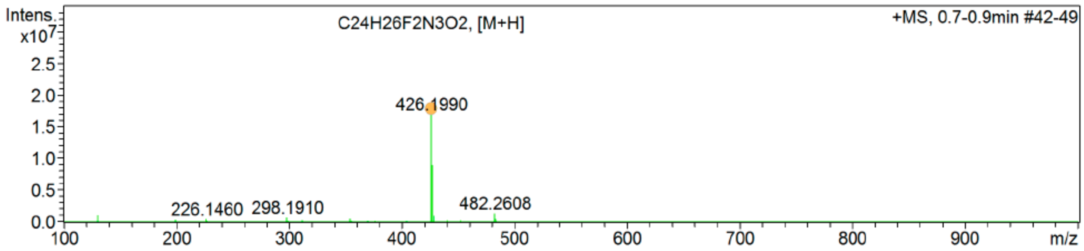

Meas. m/z	#	Ion Formula	m/z	err [ppm]	mSigma	# mSigma	Score	rdb	e⁻ Conf	N-Rule
426.1990	1	$C_{24}H_{26}F_2N_3O_2$	426.1988	-0.7	150.0	1	100.00	12.5	even	ok
	2	$C_{21}H_{27}F_3N_3O_3$	426.1999	2.0	168.7	2	20.59	8.5	even	ok
	3	$C_{19}H_{24}N_9O_3$	426.1997	1.5	168.7	3	23.31	12.5	even	ok
	4	$C_{18}H_{28}N_5O_7$	426.1983	-1.7	182.0	4	8.16	7.5	even	ok
	5	$C_{15}H_{29}FN_5O_8$	426.1995	1.0	200.6	5	2.08	3.5	even	ok
	6	$C_9H_{22}F_2N_{15}O_3$	426.1993	0.5	219.3	6	0.44	5.5	even	ok

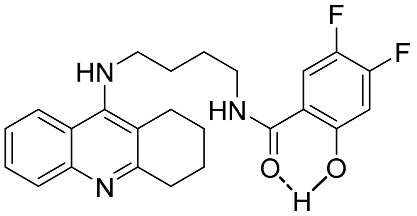

GMV-161c.23i-C.EP180.6240_23i1300.d

Bruker Compass DataAnalysis 4.2 printed: 9/23/2021 1:08:14 PM by: admin Page 1 of 1

Institute of Organic Synthesis UB RAS
22 S.Kovalevskoy, 20 Akademicheskaya str, Yekaterinburg, Russian Federation
Phone: +7 (343) 362-34-56

Figure S15. HMRS spectrum of compound **7a**

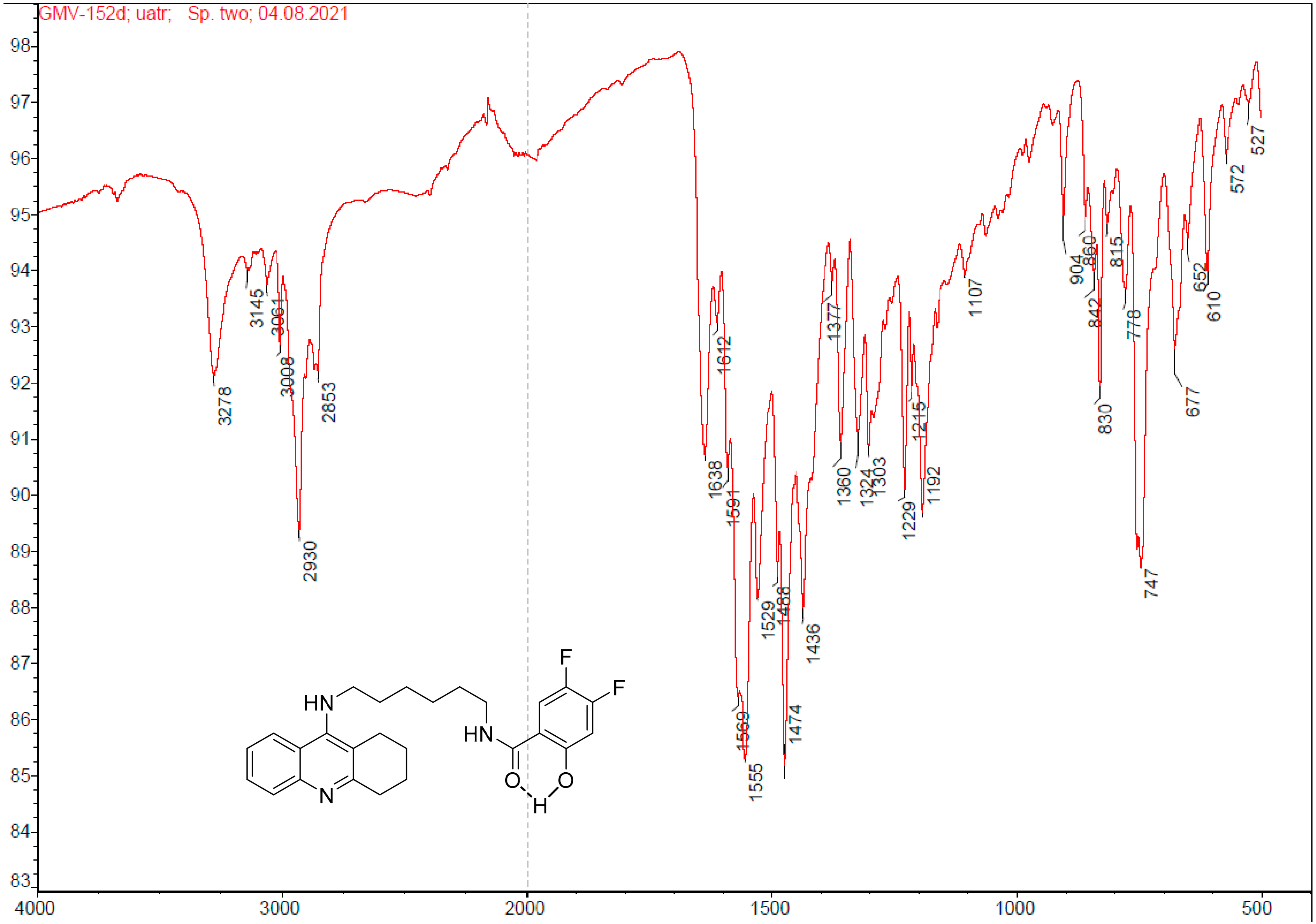

Figure S16. IR spectrum of compound **7b**

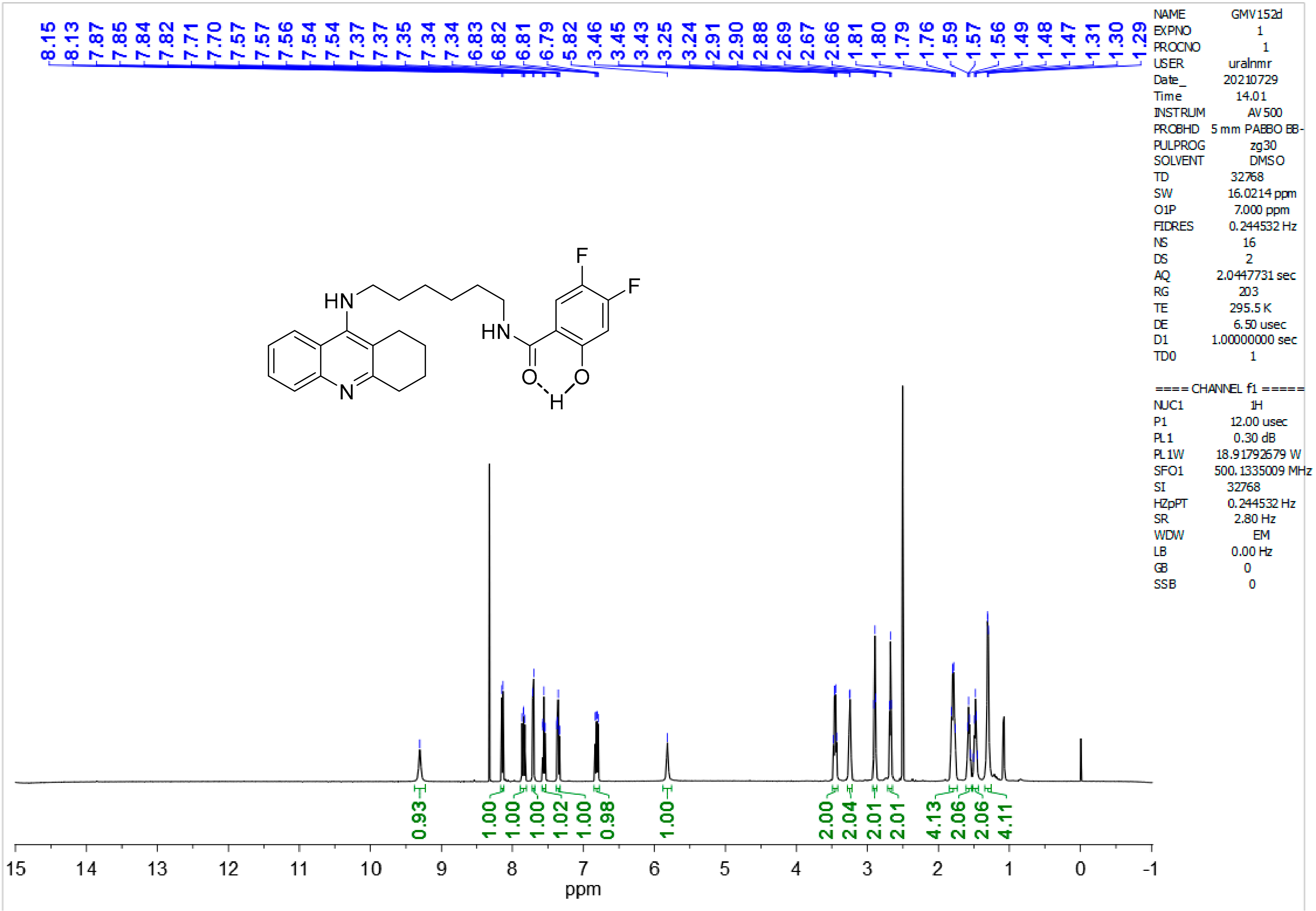

Figure S17. ¹H NMR spectrum of compound **7b**

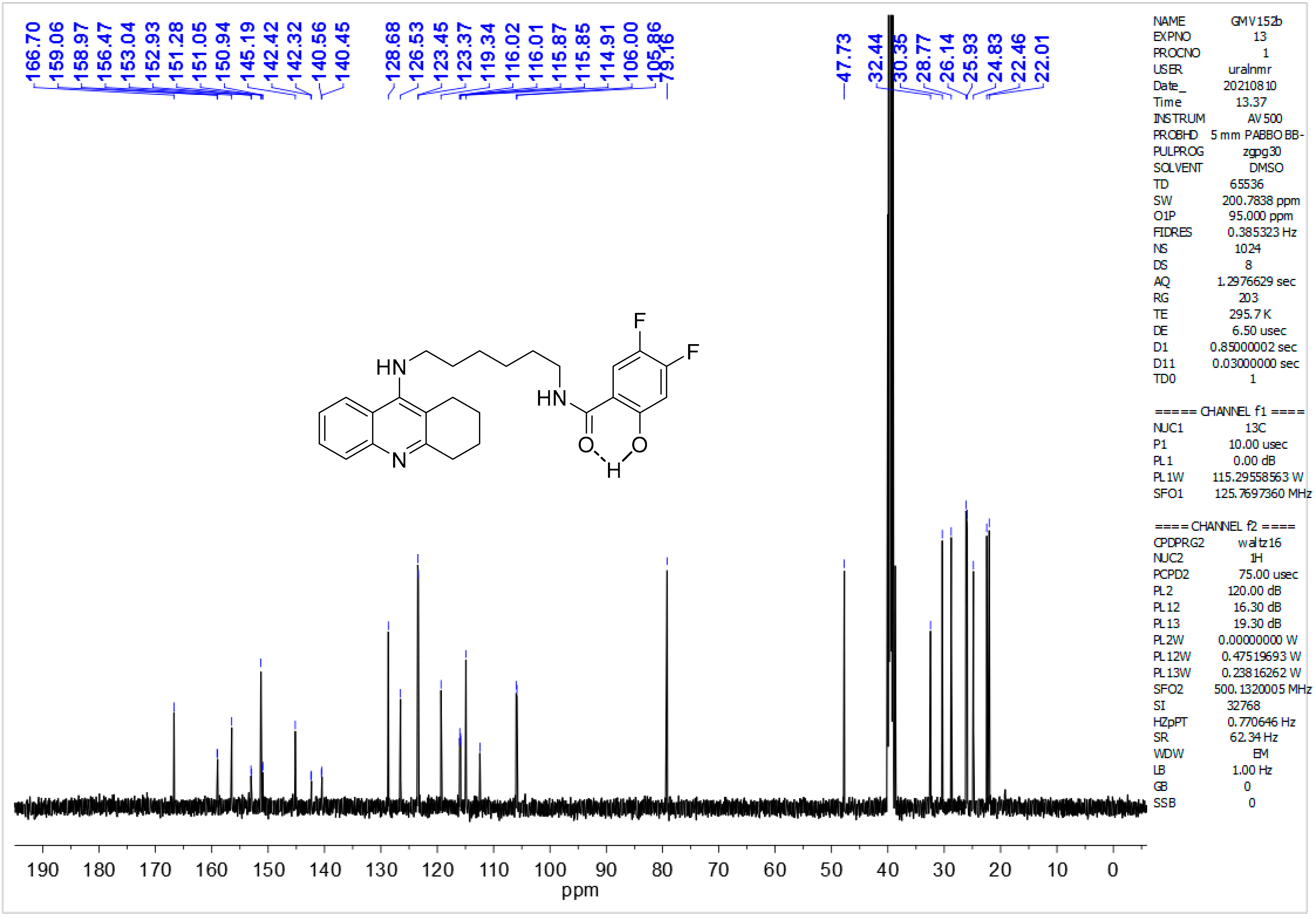

Figure S18. ^{13}C NMR spectrum of compound **7b**

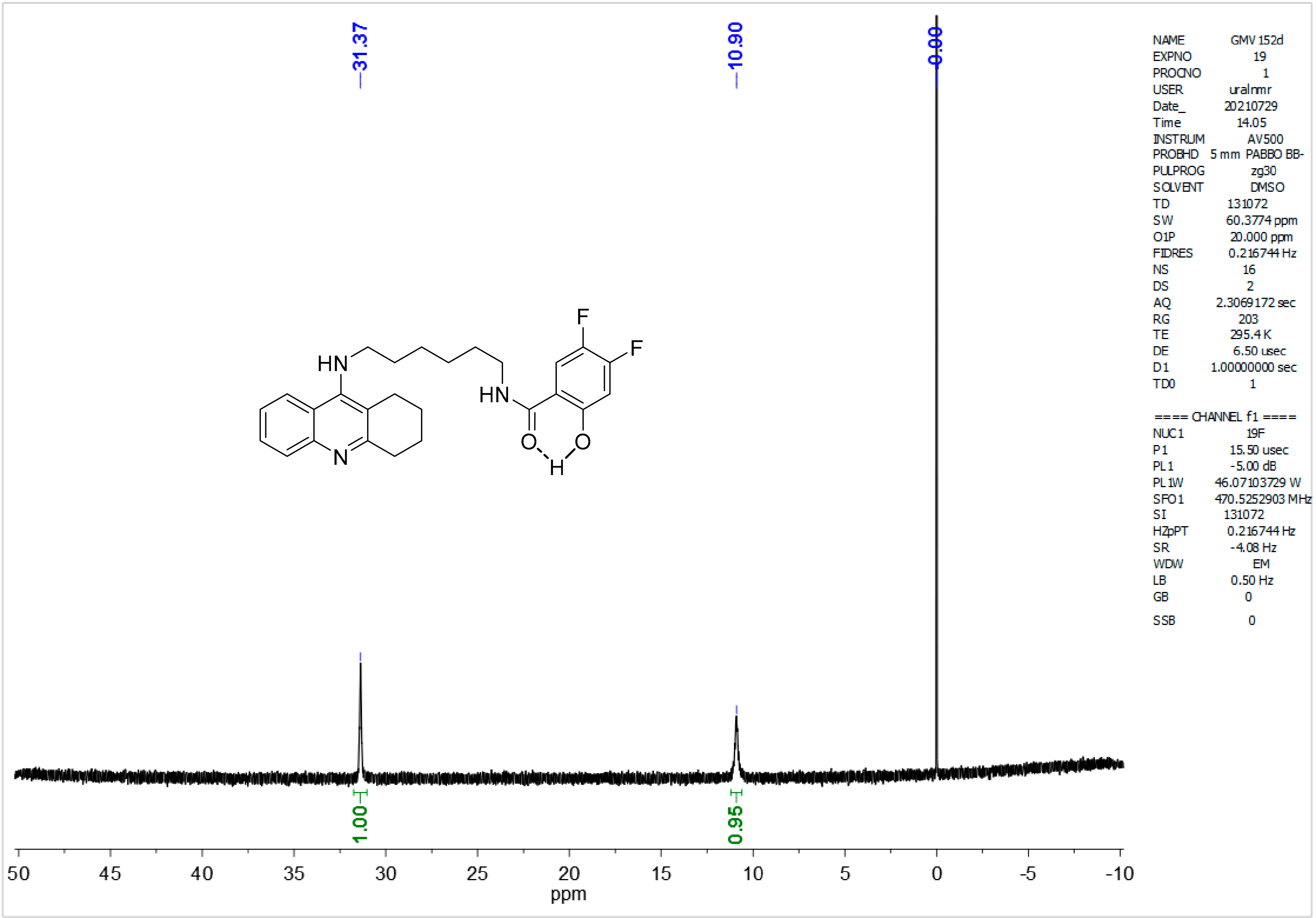

Figure S19. ^{19}F NMR spectrum of compound **7b**

Compound Spectrum SmartFormula Report

Analysis Info

Analysis Name	D:\Data\ING21\GMV-152d.22i-C.EP180.6225_23i1150.d	Acquisition Date	9/23/2021 11:50:13 AM
Method	EP180UI21HPC50-1600_500-3500-0.4-4-200_1f2002f200hrf 70ie5lm70ce10pps6crf300-1200tt40-110_F3x1_Segm1.m	Operator	admin
Sample Name		Instrument	maXis impact 1819696.00172

Comment: 2/09/2022: +Bckgnd: 118.09, 322.05, 622.03, 922.01, 1221.99, 1521.97, 1821.95, 2121.93, 2421.91, 2721.89 (G1969-85000; +/-299.981 HPC); other intense peaks (>2*e4): 102.13 (NEt3); 132.91 (*2-PrOH); 391.28&413.26 (DOP); 86.10, 113.13, 140.07, 149.02, 158.96, 167.03, 187.07. 194.10, 203.14, 207.17, 209.19, 214.25, 217.10, 223.21, 227.23, 237.22, 245.19, 249.22, 251.24, 255.27, 259.20, 263.23, 265.25, 273.22, 279.16, 291.27, 293.28, 304.30, 307.30, 321.31, 326.38, 332.33, 335.33, 349.35, 413.27, 1259.95, 1307.08, 1559.93: background (prev. analyzed samples and impurities); 188.09 (#6216); 588.32 (#6218); 404.23 (#6219); 376.20 (#6220); 440.21 (#6221); 460.30 (#6224)

Acquisition Parameter

Source Type	ESI	Ion Polarity	Positive	Set Nebulizer	0.4 Bar
Focus	Active	Set Capillary	3500 V	Set Dry Heater	200 °C
Scan Begin	50 m/z	Set End Plate Offset	-500 V	Set Dry Gas	4.0 l/min
Scan End	1600 m/z	Set Charging Voltage	2000 V	Set Divert Valve	Source
		Set Corona	0 nA	Set APCI Heater	0 °C

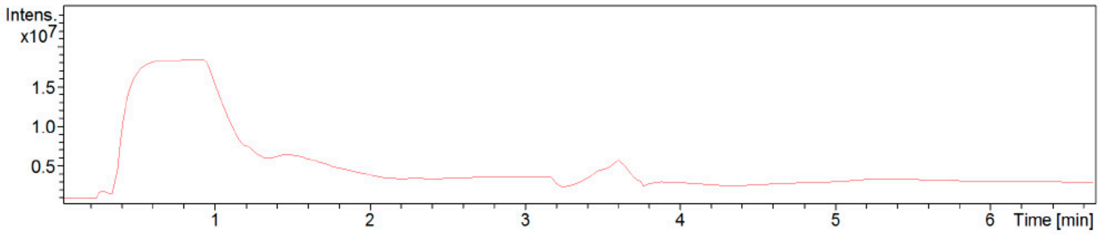

+MS, 0.5-0.6min #28-33

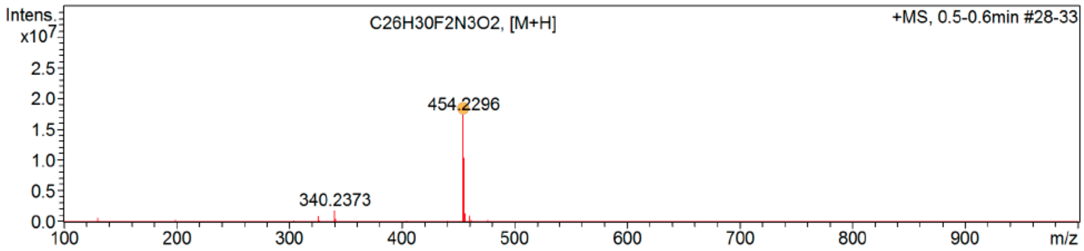

Meas. m/z	#	Ion Formula	m/z	err [ppm]	mSigma	# mSigma	Score	rdb	e⁻ Conf	N-Rule
454.2296	1	C29H29FN3O	454.2289	-1.5	158.3	1	100.00	16.5	even	ok
	2	C26H30F2N3O2	454.2301	1.0	177.1	2	28.33	12.5	even	ok
	3	C20H32N5O7	454.2296	0.1	209.2	3	2.48	7.5	even	ok
	4	C14H25FN15O2	454.2294	-0.4	228.1	4	0.41	9.5	even	ok
	5	C10H30F2N11O7	454.2292	-0.8	259.8	5	0.02	0.5	even	ok

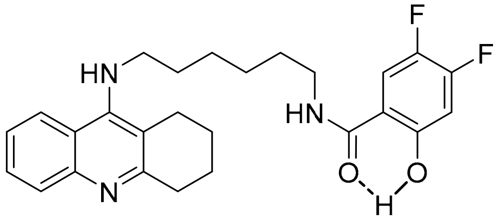

GMV-152d.22i-C.EP180.6225_23i1150.d

Bruker Compass DataAnalysis 4.2 printed: 9/23/2021 11:58:05 AM by: admin Page 1 of 1

Institute of Organic Synthesis UB RAS
22 S.Kovalevskoy, 20 Akademicheskaya str, Yekaterinburg, Russian Federation
Phone: +7 (343) 362-34-56

Figure S20. HRMS spectrum of compound **7b**

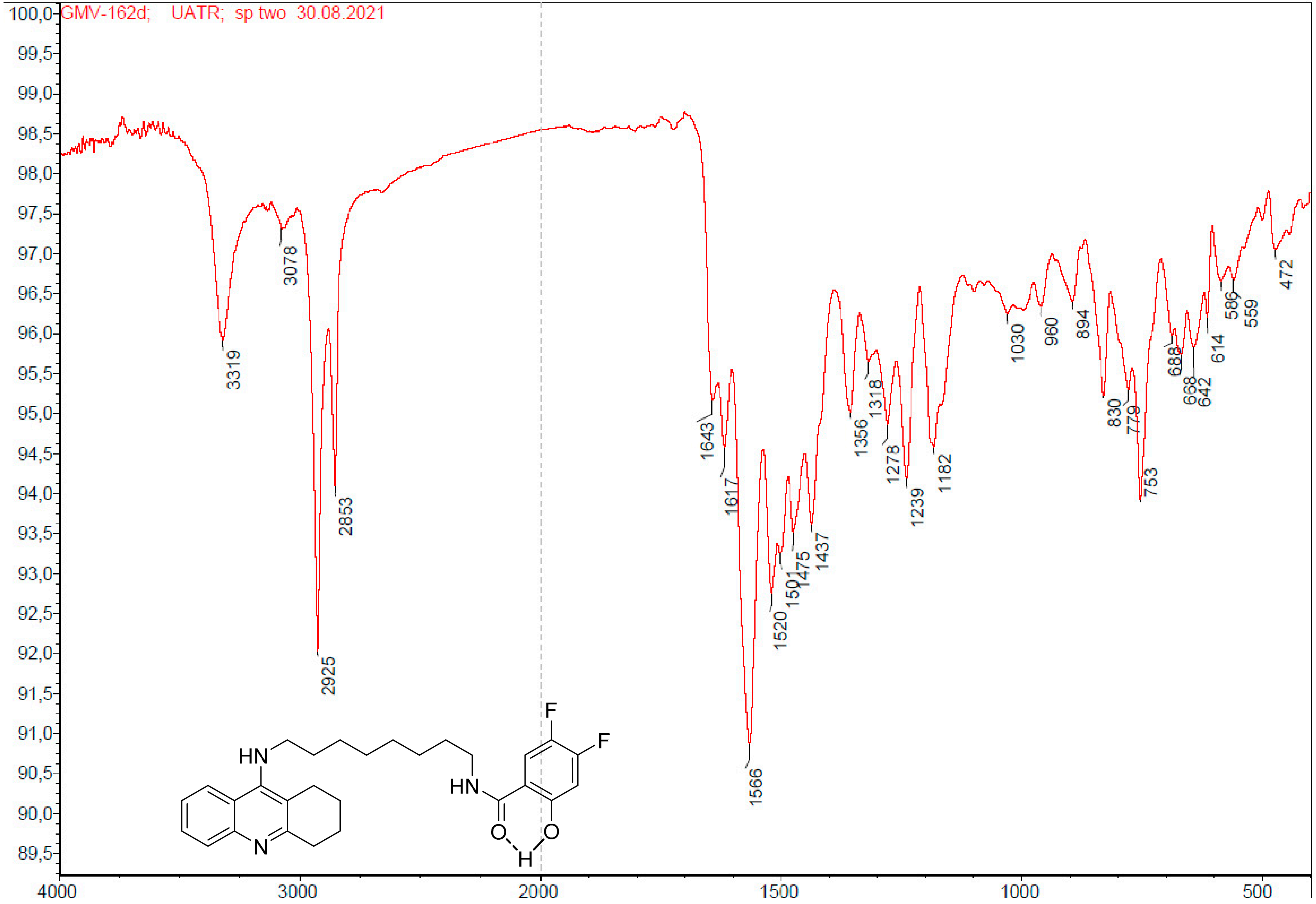

Figure S21. IR spectrum of compound **7c**

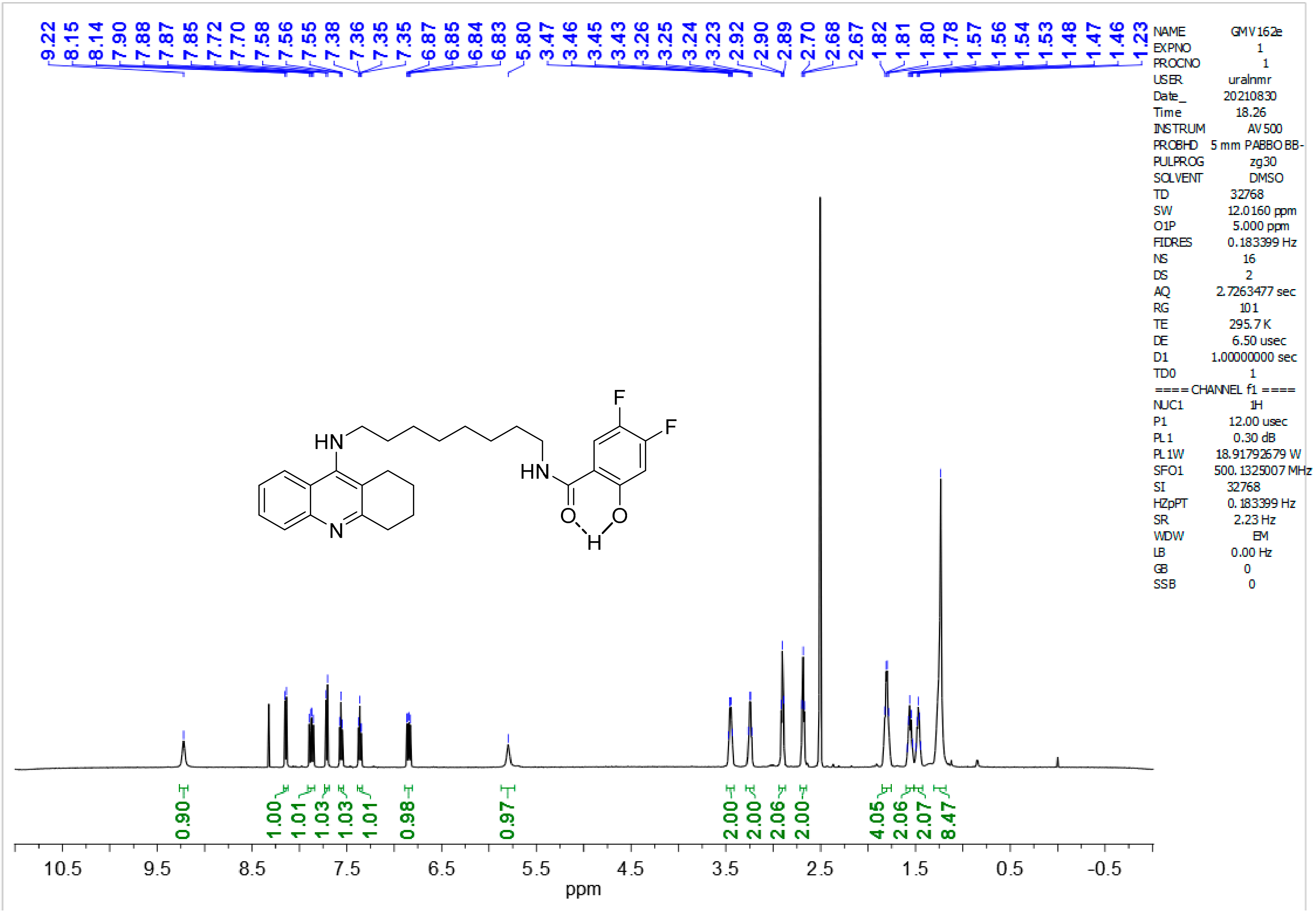

Figure S22. ^{1}H NMR spectrum of compound **7c**

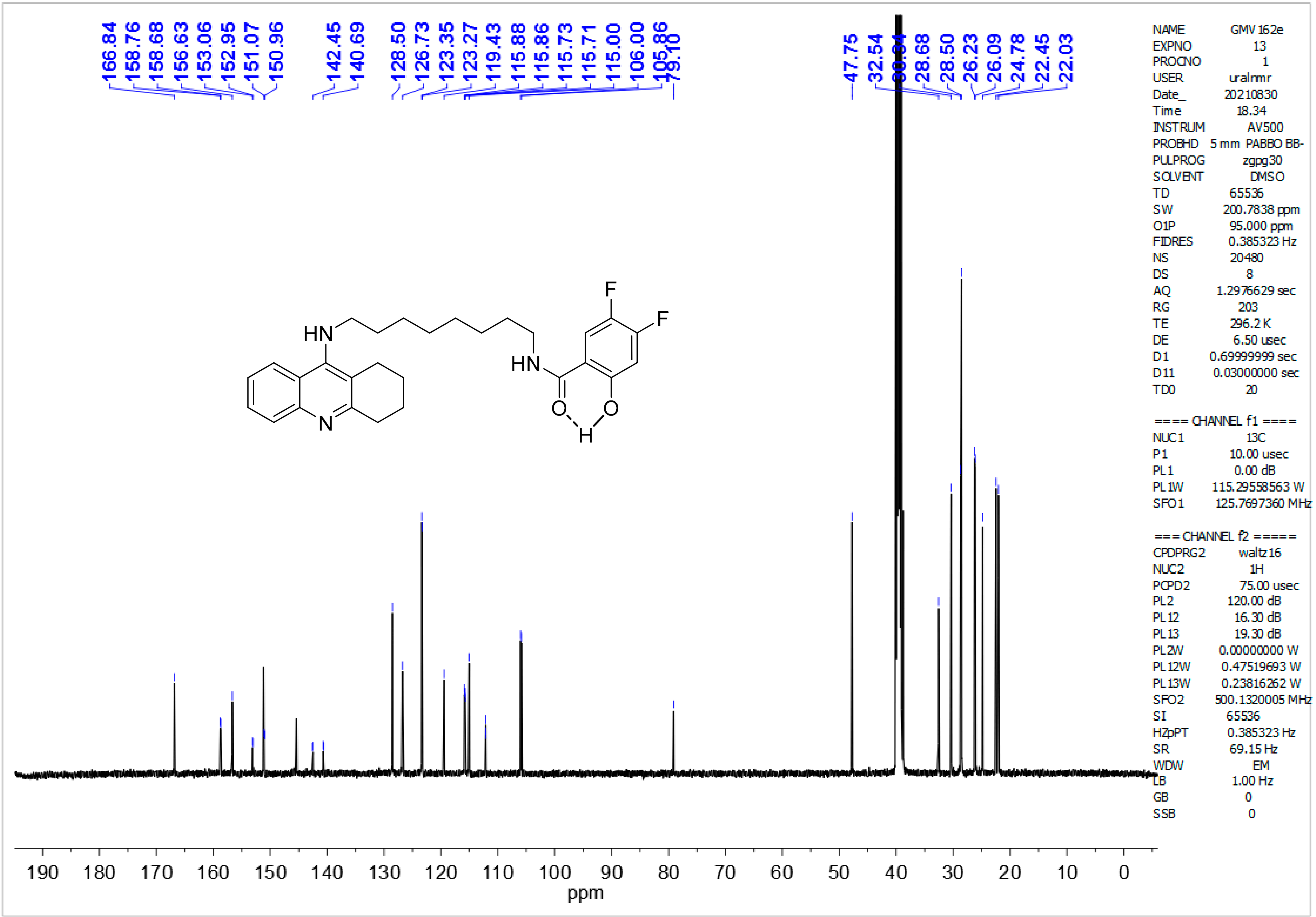

Figure S23. ^{13}C NMR spectrum of compound **7c**

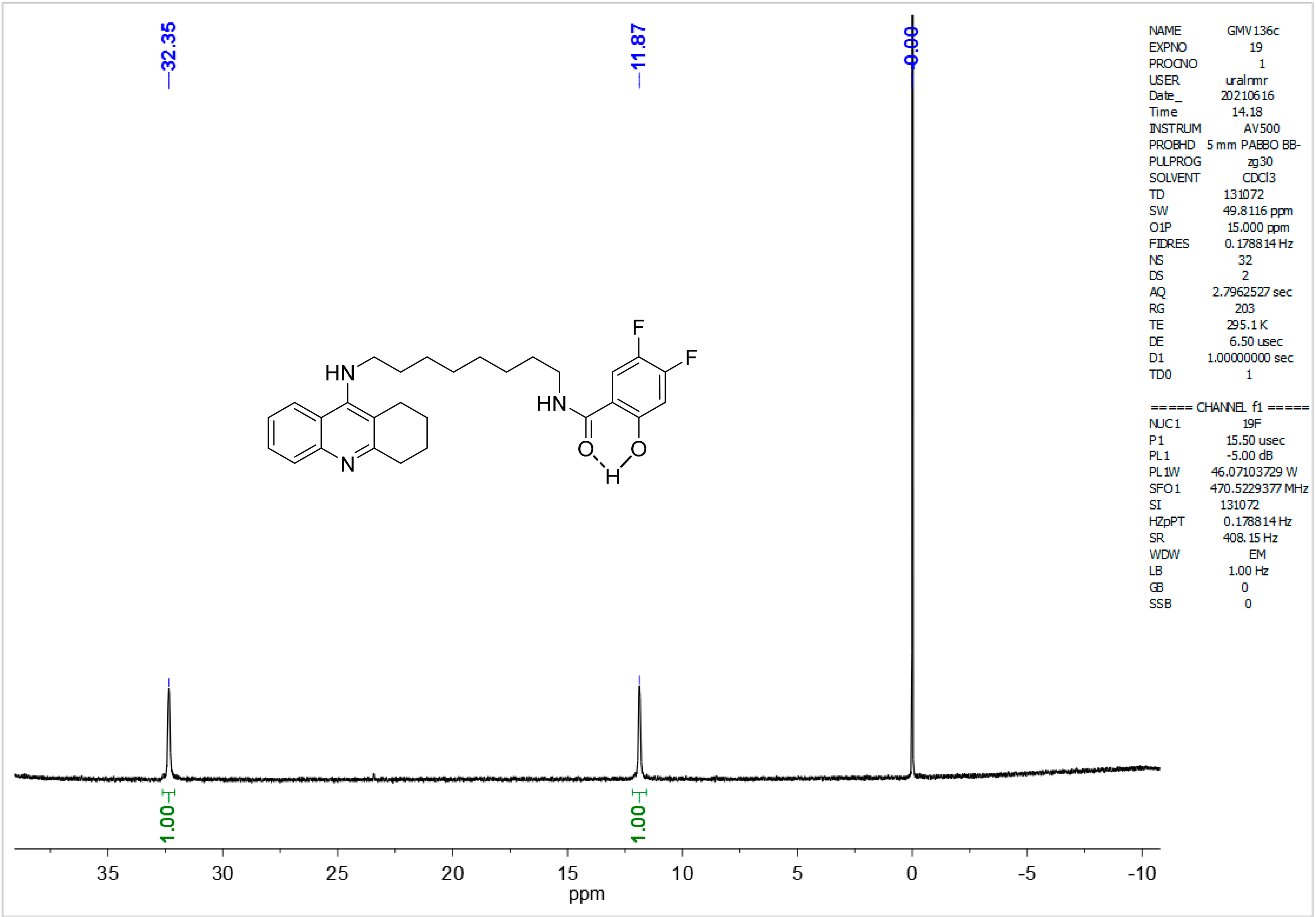

Figure S24. ^{19}F NMR spectrum of compound **7c**

Compound Spectrum SmartFormula Report

Analysis Info Acquisition Date 9/23/2021 12:35:03 PM

Analysis Name	D:\Data\ING21\GMV-162d.23i-C.EP180.6239_23i1230.d
Method	EP180UI21HPC50-1600_500-3500-0.4-4-200_1f2002f200hrf Operator admin<br>70ie5lm70ce10pps6crf300-1200tt40-110_F3x1_Segm1.m
Sample Name	Instrument maXis impact 1819696.00172
Comment	2/09/2022: +Bckgnd: 118.09, 322.05, 622.03, 922.01, 1221.99, 1521.97, 1821.95, 2121.93, 2421.91, 2721.89 (G1969-85000; +/-299.981 HPC); other intense peaks (>2*e4): 102.13 (NEt3); 132.91 (*2-PrOH); 391.28&413.26 (DOP); 86.10, 113.13, 140.07, 149.02, 158.96, 167.03, 187.07. 194.10, 203.14, 207.17, 209.19, 214.25, 217.10, 223.21, 227.23, 237.22, 245.19, 249.22, 251.24, 255.27, 259.20, 263.23, 265.25, 273.22, 279.16, 291.27, 293.28, 304.30, 307.30, 321.31, 326.38, 332.33, 335.33, 349.35, 413.27, 1259.95, 1307.08, 1559.93: background (prev. analyzed samples and impurities); 188.09 (#6216); 588.32 (#6218); 404.23 (#6219); 376.20 (#6220); 440.21 (#6221); 460.30 (#6224)

Acquisition Parameter

Source Type	ESI	Ion Polarity	Positive	Set Nebulizer	0.4 Bar
Focus	Active	Set Capillary	3500 V	Set Dry Heater	200 °C
Scan Begin	50 m/z	Set End Plate Offset	-500 V	Set Dry Gas	4.0 l/min
Scan End	1600 m/z	Set Charging Voltage	2000 V	Set Divert Valve	Source
		Set Corona	0 nA	Set APCI Heater	0 °C

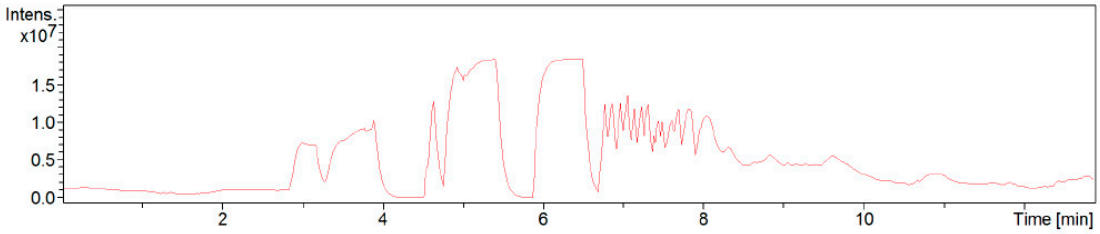

+MS, 7.0-7.5min #403-434

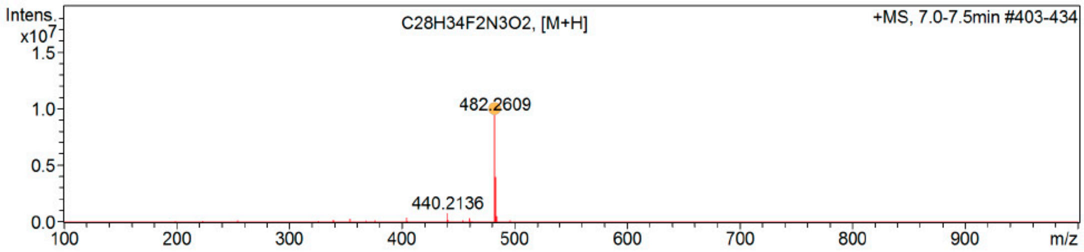

Meas. m/z	#	Ion Formula	m/z	err [ppm]	mSigma	# mSigma	Score	rdb	e⁻ Conf	N-Rule
482.2609	1	C31H33FN3O	482.2602	-1.5	37.9	1	100.00	16.5	even	ok
	2	C28H34F2N3O2	482.2614	0.9	62.1	2	41.94	12.5	even	ok
	3	C23H32N9O3	482.2623	2.7	80.8	3	12.69	12.5	even	ok
	4	C21H40NO11	482.2596	-2.8	93.3	4	9.61	2.5	even	ok
	5	C22H36N5O7	482.2609	-0.1	94.3	5	13.78	7.5	even	ok
	6	C21H31F3N9O	482.2598	-2.3	94.3	6	7.92	9.5	even	ok
	7	C19H28N15O	482.2596	-2.8	94.4	7	6.81	13.5	even	ok
	8	C19H37FN5O8	482.2621	2.3	112.9	8	3.23	3.5	even	ok
	9	C16H29FN15O2	482.2607	-0.5	113.1	9	5.04	9.5	even	ok
	10	C13H30F2N15O3	482.2619	1.9	131.7	10	1.28	5.5	even	ok
	11	C12H34F2N11O7	482.2605	-0.9	144.9	11	0.73	0.5	even	ok

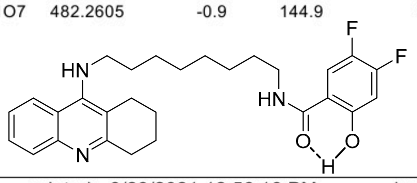

GMV-162d.23i-C.EP180.6239_23i1230.d
Bruker Compass DataAnalysis 4.2 printed: 9/23/2021 12:56:16 PM by: admin Page 1 of 1

Institute of Organic Synthesis UB RAS
22 S.Kovalevskoy, 20 Akademicheskaya str, Yekaterinburg, Russian Federation
Phone: +7 (343) 362-34-56

Figure S25. HMRS spectrum of compound **7c**

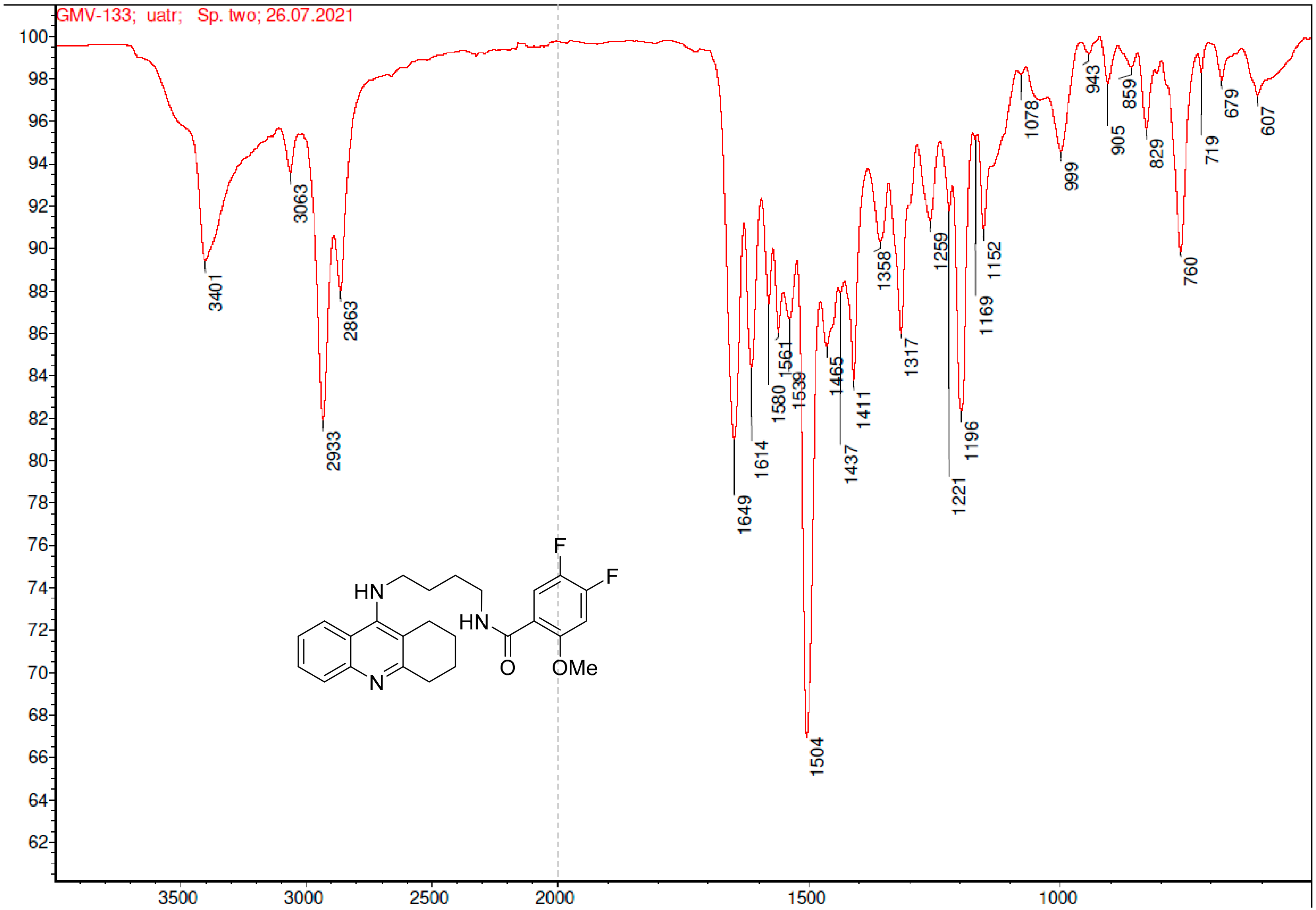

Figure S26. IR spectrum of compound **8a**

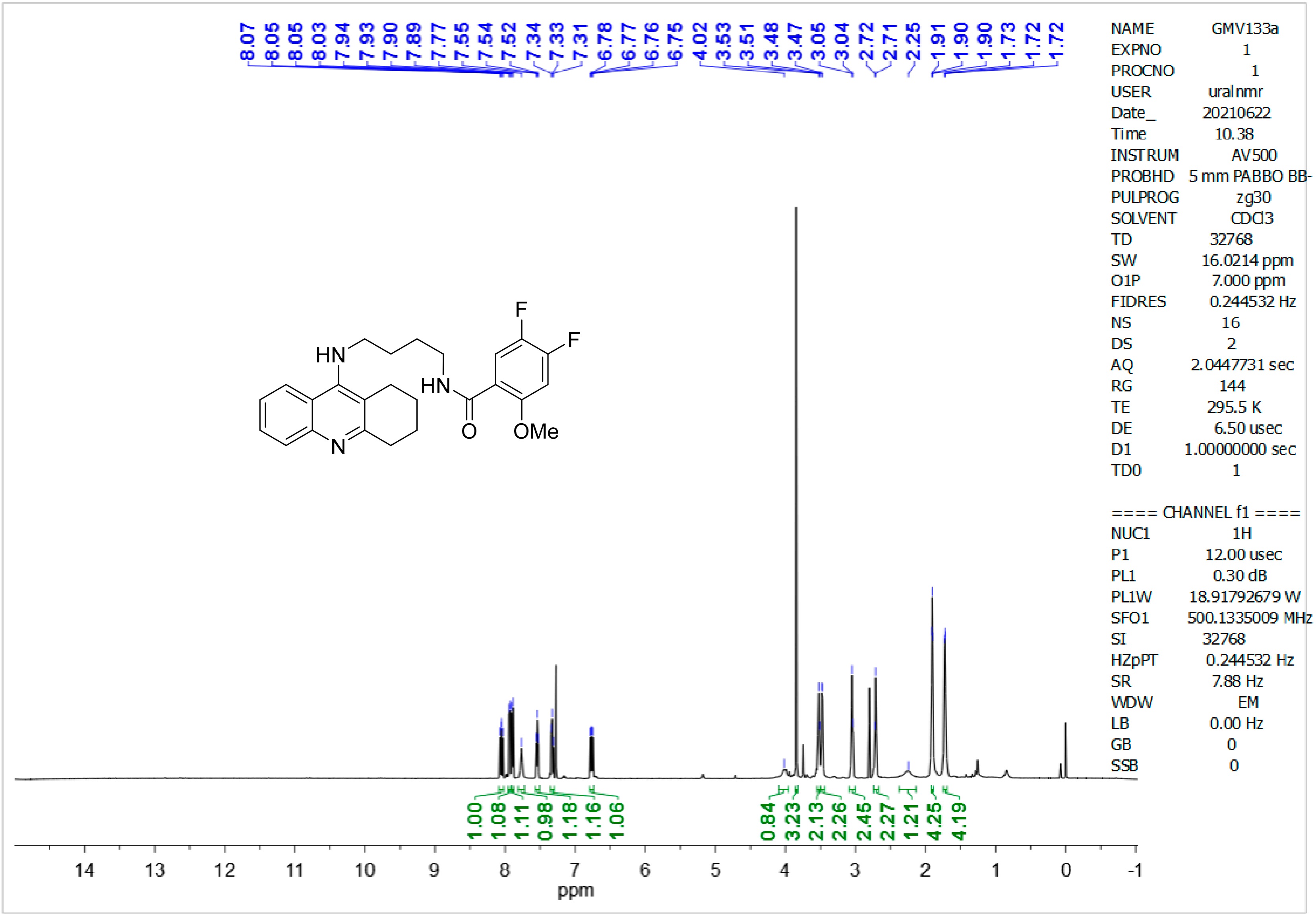

Figure S26. ^{1}H NMR spectrum of compound **8a**

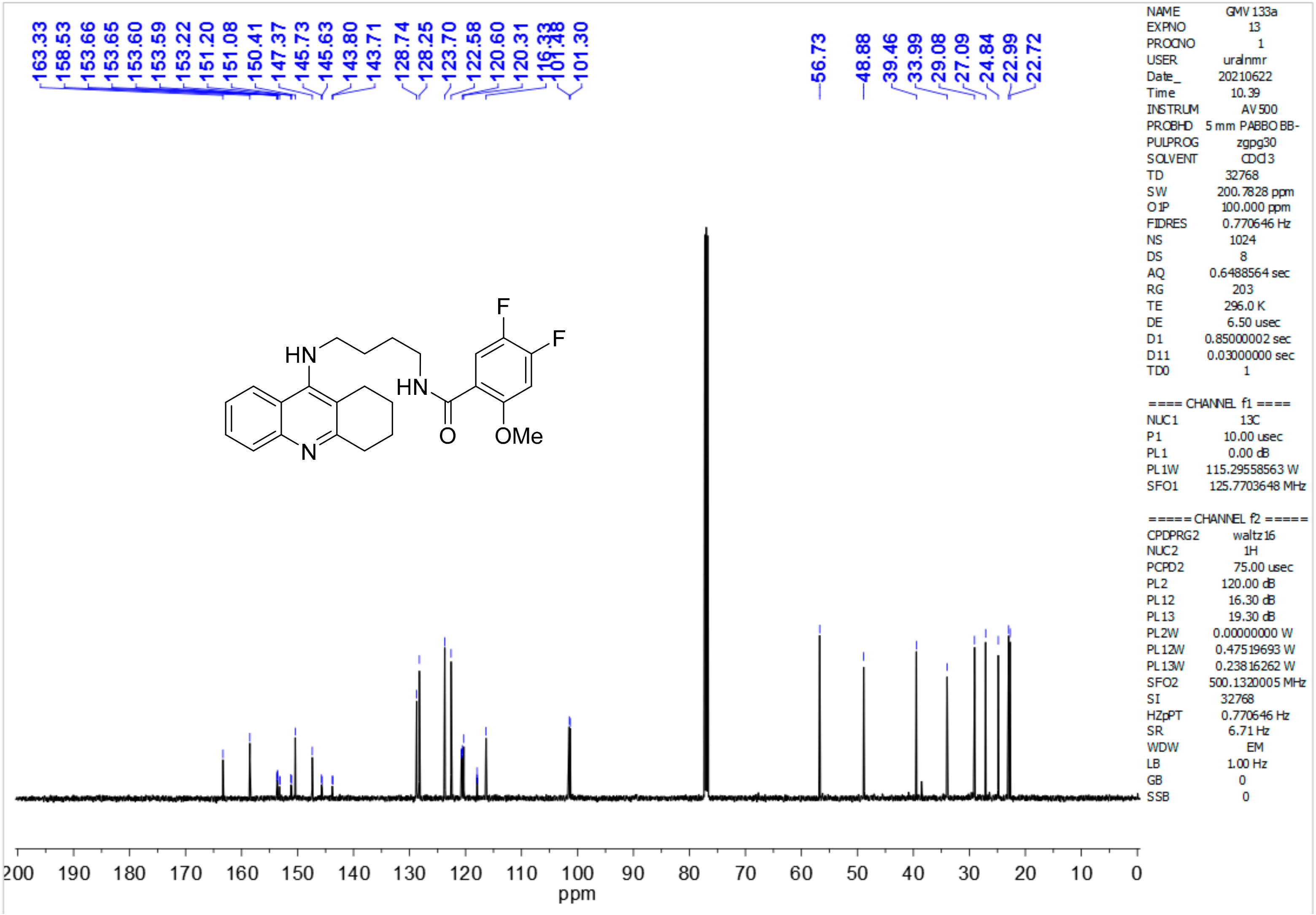

Figure S27. ^{13}C NMR spectrum of compound **8a**

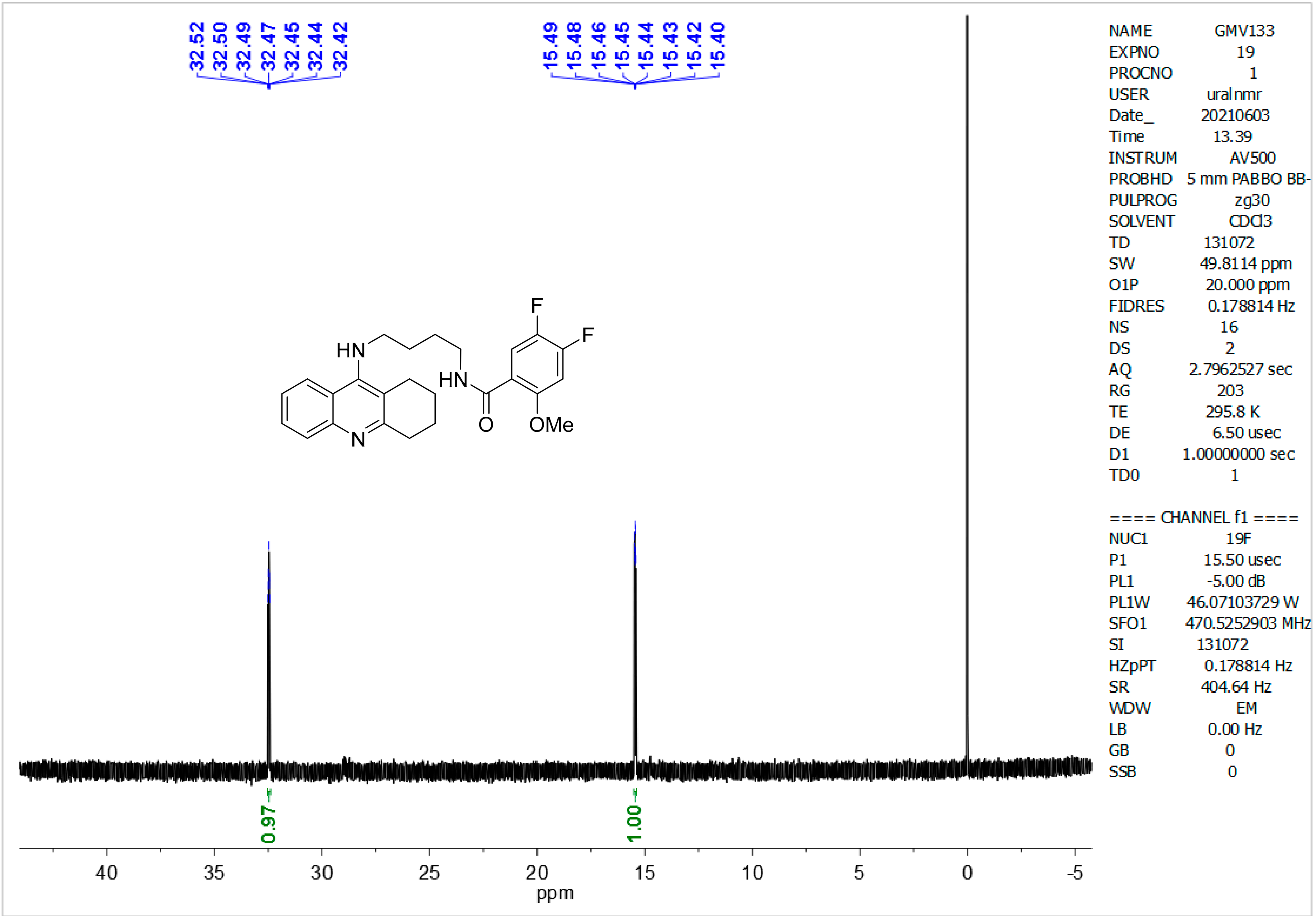

Figure S28. ^{19}F NMR spectrum of compound **8a**

Compound Spectrum SmartFormula Report

Analysis Info

Acquisition Date 9/22/2021 2:38:48 PM

Analysis Name D:\Data\ING21\GMV-133.22i-C.EP180.6221_22i1440.d

Method EP180UI21HPC50-1600_500-3500-0.4-4-200_1f2002f200hrf 70ie5lm70ce10pps6crf300-1200tt40-110_F3x1_Segm1.m Operator admin

Sample Name

Instrument maXis impact 1819696.00172

Comment 22/09/2022: +Bckgnd: 118.09, 322.05, 622.03, 922.01, 1221.99, 1521.97, 1821.95, 2121.93, 2421.91, 2721.89 (G1969-85000; +/-299.981 HPC); other intense peaks (>2*e4): 102.13 (NEt3); 132.91 (*2-PrOH); 391.28&413.26 (DOP); 86.10, 113.13, 140.07, 149.02, 158.96, 167.03, 187.07. 194.10, 203.14, 207.17, 209.19, 214.25, 217.10, 223.21, 227.23, 237.22, 245.19, 249.22, 251.24, 255.27, 259.20, 263.23, 265.25, 273.22, 279.16, 291.27, 293.28, 304.30, 307.30, 321.31, 326.38, 332.33, 335.33, 349.35, 413.27, 1259.95, 1307.08, 1559.93: background (prev. analyzed samples and impurities); 188.09 (#6216); 588.32 (#6218); 404.23 (#6219); 376.20 (#6220)

Acquisition Parameter

Source Type	ESI	Ion Polarity	Positive	Set Nebulizer	0.4 Bar
Focus	Active	Set Capillary	3500 V	Set Dry Heater	200 °C
Scan Begin	50 m/z	Set End Plate Offset	-500 V	Set Dry Gas	4.0 l/min
Scan End	1600 m/z	Set Charging Voltage	2000 V	Set Divert Valve	Source
		Set Corona	0 nA	Set APCI Heater	0 °C

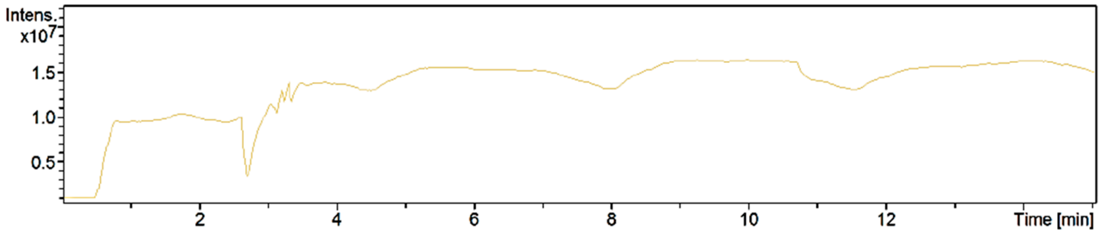

+MS, 13.7-14.3min #791-821

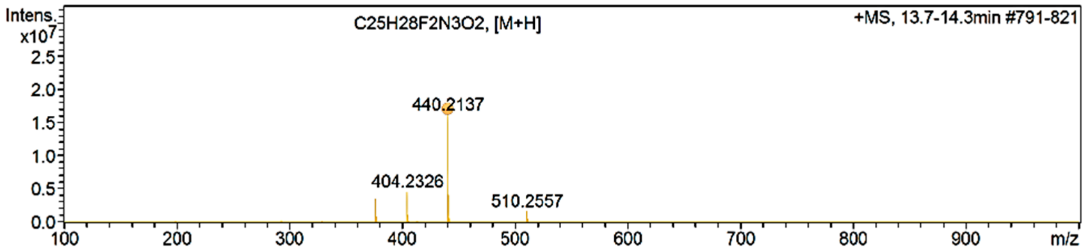

Meas. m/z	#	Ion Formula	m/z	err [ppm]	mSigma	# mSigma	Score	rdb	e⁻ Conf	N-Rule
440.2137	1	C25H28F2N3O2	440.2144	1.7	15.0	1	87.43	12.5	even	ok
	2	C19H30N5O7	440.2140	0.7	18.8	2	100.00	7.5	even	ok
	3	C28H27FN3O	440.2133	-0.9	33.8	3	67.65	16.5	even	ok

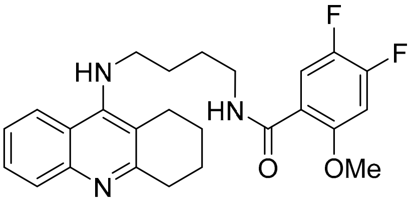

GMV-133.22i-C.EP180.6221_22i1440.d

Bruker Compass DataAnalysis 4.2 printed: 9/22/2021 4:19:21 PM by: admin Page 1 of 1

Institute of Organic Synthesis UB RAS
22 S.Kovalevskoy, 20 Akademicheskaya str, Yekaterinburg, Russian Federation
Phone: +7 (343) 362-34-56

BRUKER

Figure S29. HRMS spectrum of compound **8a**

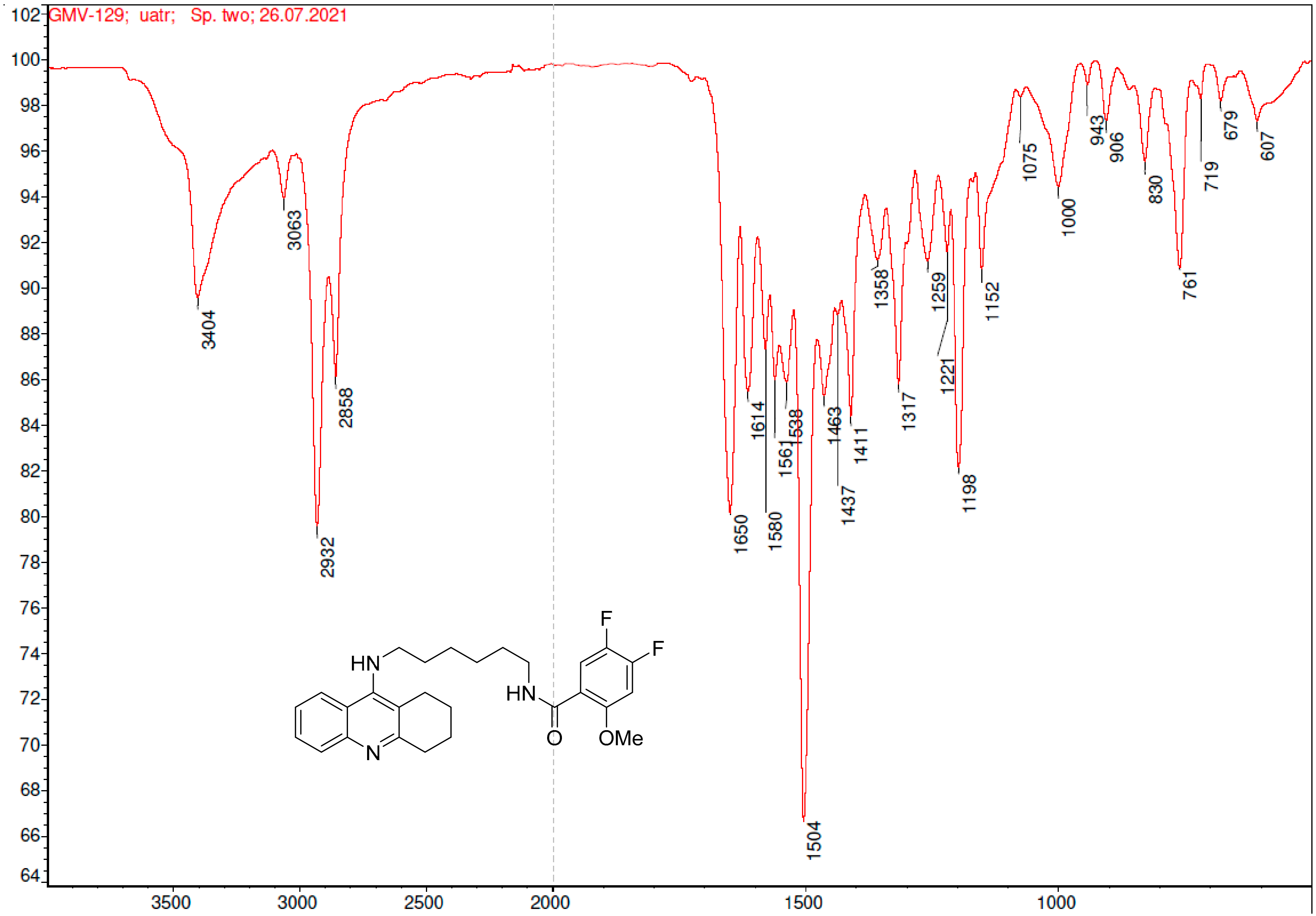

Figure S30. IR spectrum of compound **8b**

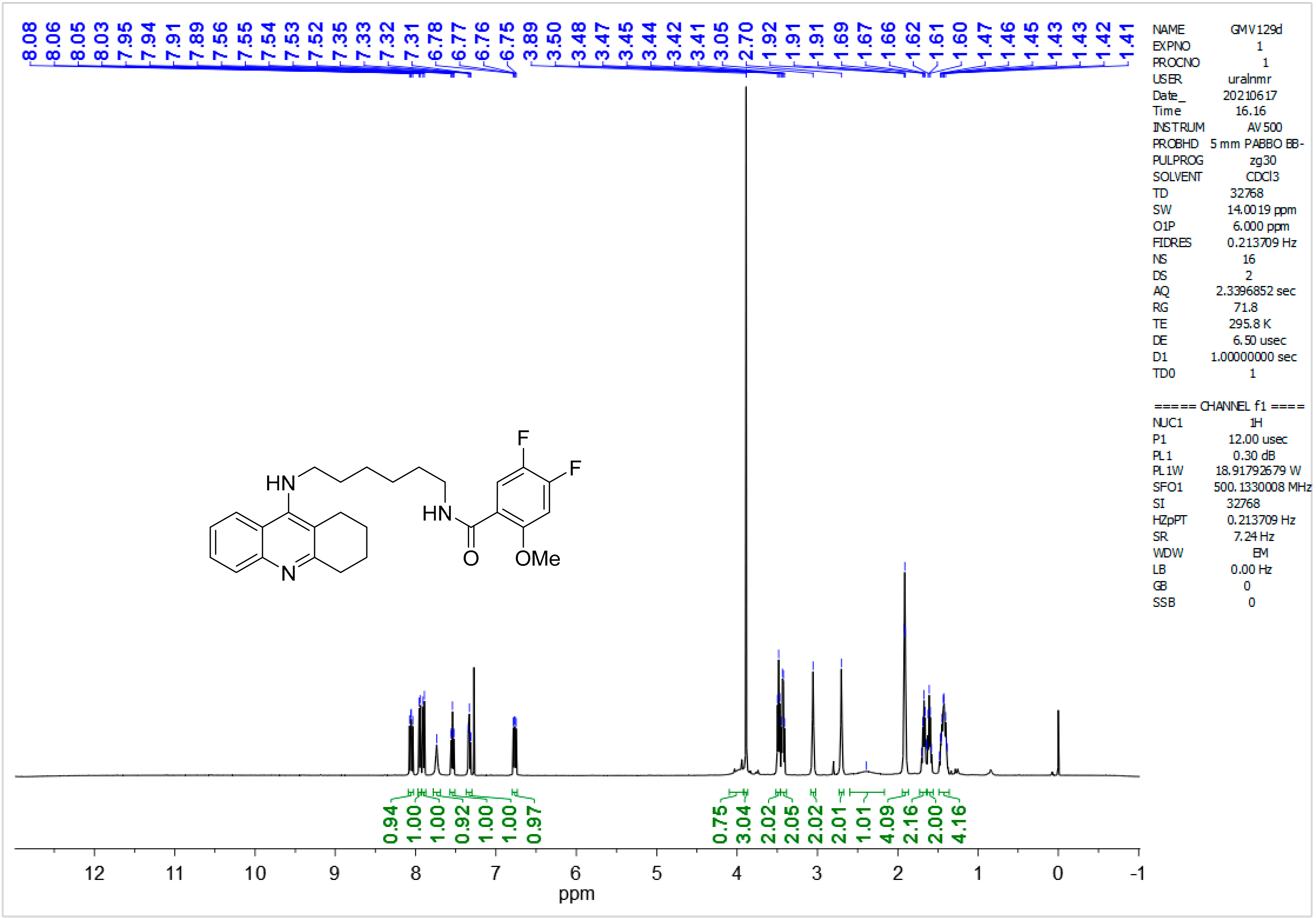

Figure S31. ^{1}H NMR spectrum of compound **8b**

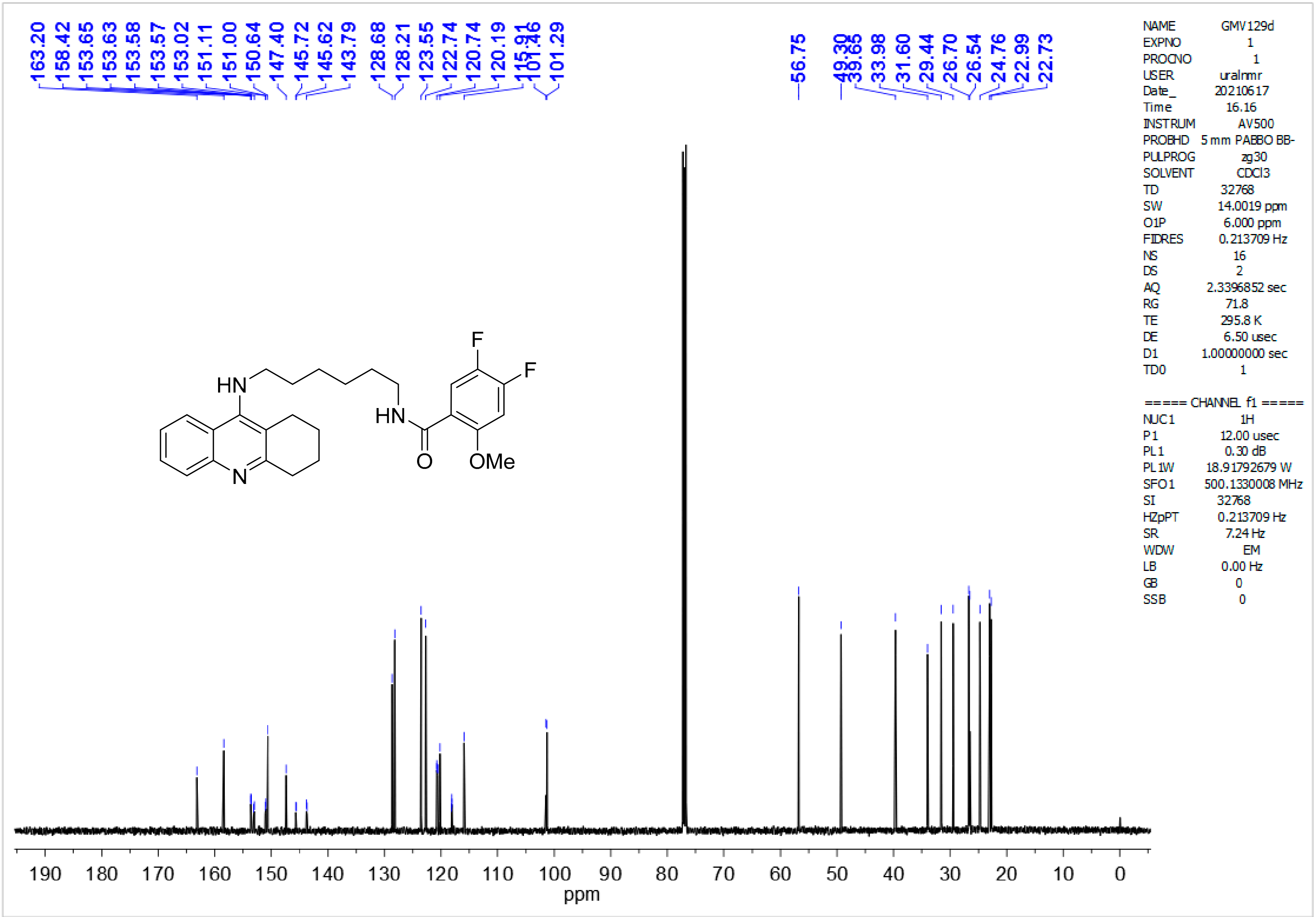

Figure S33. ^{13}C NMR spectrum of compound **8b**

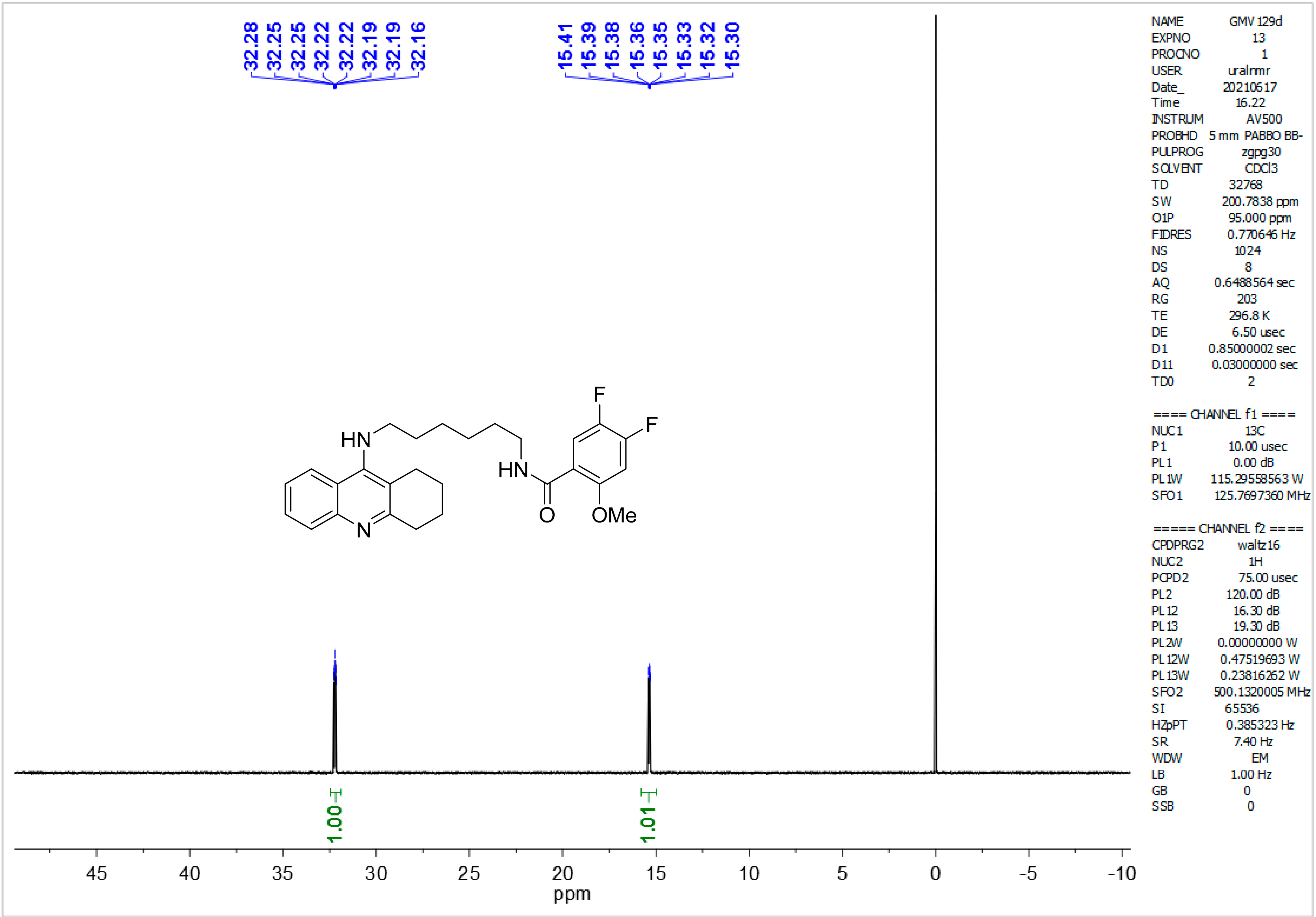

Figure S34. ^{19}F NMR spectrum of compound **8b**

Compound Spectrum SmartFormula Report

Analysis Info

Acquisition Date 6/25/2021 4:20:38 PM

Analysis Name D:\Data\ING21\GMV-129.25F-C.6122_EP_22_01_1072.d
Method ep180_50-1300_tuneposstd-hoona-uf19_1f2002f200hrf80ie5lOperator admin
m70ce6crf300-1200tt50-110pps6x1_fst_segm3.m
Sample Name GMV-129.25F-C.6122_EP Instrument maXis impact 1819696.00172
Comment

Acquisition Parameter

Source Type	ESI	Ion Polarity	Positive	Set Nebulizer	4.0 Bar
Focus	Active	Set Capillary	3500 V	Set Dry Heater	200 °C
Scan Begin	50 m/z	Set End Plate Offset	-500 V	Set Dry Gas	8.0 l/min
Scan End	1300 m/z	Set Charging Voltage	2000 V	Set Divert Valve	Source
		Set Corona	0 nA	Set APCI Heater	0 °C

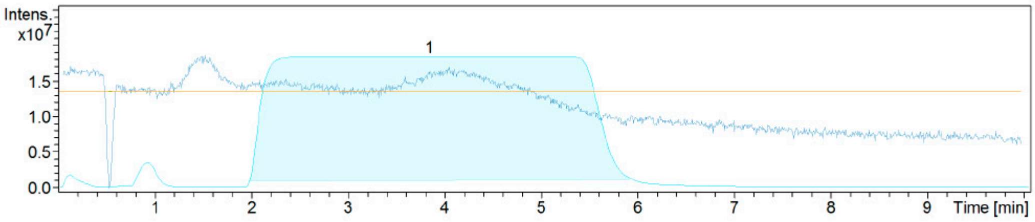

#	RT [min]	Area	Int. Type	I	S/N	Chromatogram	Max. m/z	FWHM [min]
1	3.8	3662201856	Manual	18331640	7520.5	BPC +All MS	468.2456	

Cmpd 1, 3.8 min

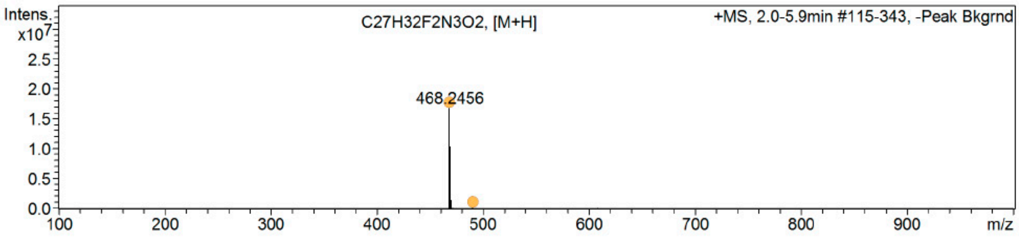

Meas. m/z	#	Ion Formula	m/z	err [ppm]	mSigma	# mSigma	Score	rdb	e⁻ Conf	N-Rule
468.2456	1	C27H32F2N3O2	468.2457	0.3	181.3	1	100.00	12.5	even	ok
	2	C21H34N5O7	468.2453	-0.6	213.4	2	6.39	7.5	even	ok
	3	C18H35FN5O8	468.2464	1.8	232.1	3	0.85	3.5	even	ok
	4	C15H27FN15O2	468.2451	-1.1	232.4	4	0.99	9.5	even	ok
	5	C12H28F2N15O3	468.2462	1.4	251.0	5	0.14	5.5	even	ok
	6	C11H32F2N11O7	468.2449	-1.5	264.2	6	0.03	0.5	even	ok
	1	C17H32FN9NaO4	468.2453	-0.5	231.5	1	100.00	5.5	even	ok
	2	C14H33F2N9NaO5	468.2465	2.0	250.1	2	10.94	1.5	even	ok
490.2261	1	C15H26FN15NaO2	490.2270	2.0	39.2	1	100.00	9.5	even	ok
	1	C11H31F2N11NaO7	490.2268	1.6	70.2	1	100.00	0.5	even	ok

+MS, 6.1-6.5min #352-377

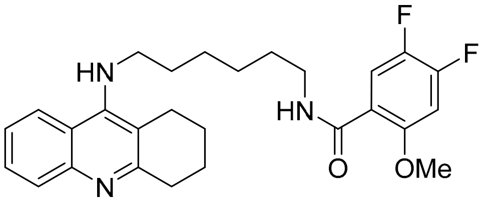

GMV-129.25F-C.6122_EP_22_01_1072.d
Bruker Compass DataAnalysis 4.2 printed: 6/25/2021 5:27:23 PM by: admin Page 1 of 2

Institute of Organic Synthesis UB RAS
22 S.Kovalevskoy, 20 Akademicheskaya str, Yekaterinburg, Russian Federation
Phone: +7 (343) 362-34-56

Figure S35. HRMS spectrum of compound **8b**

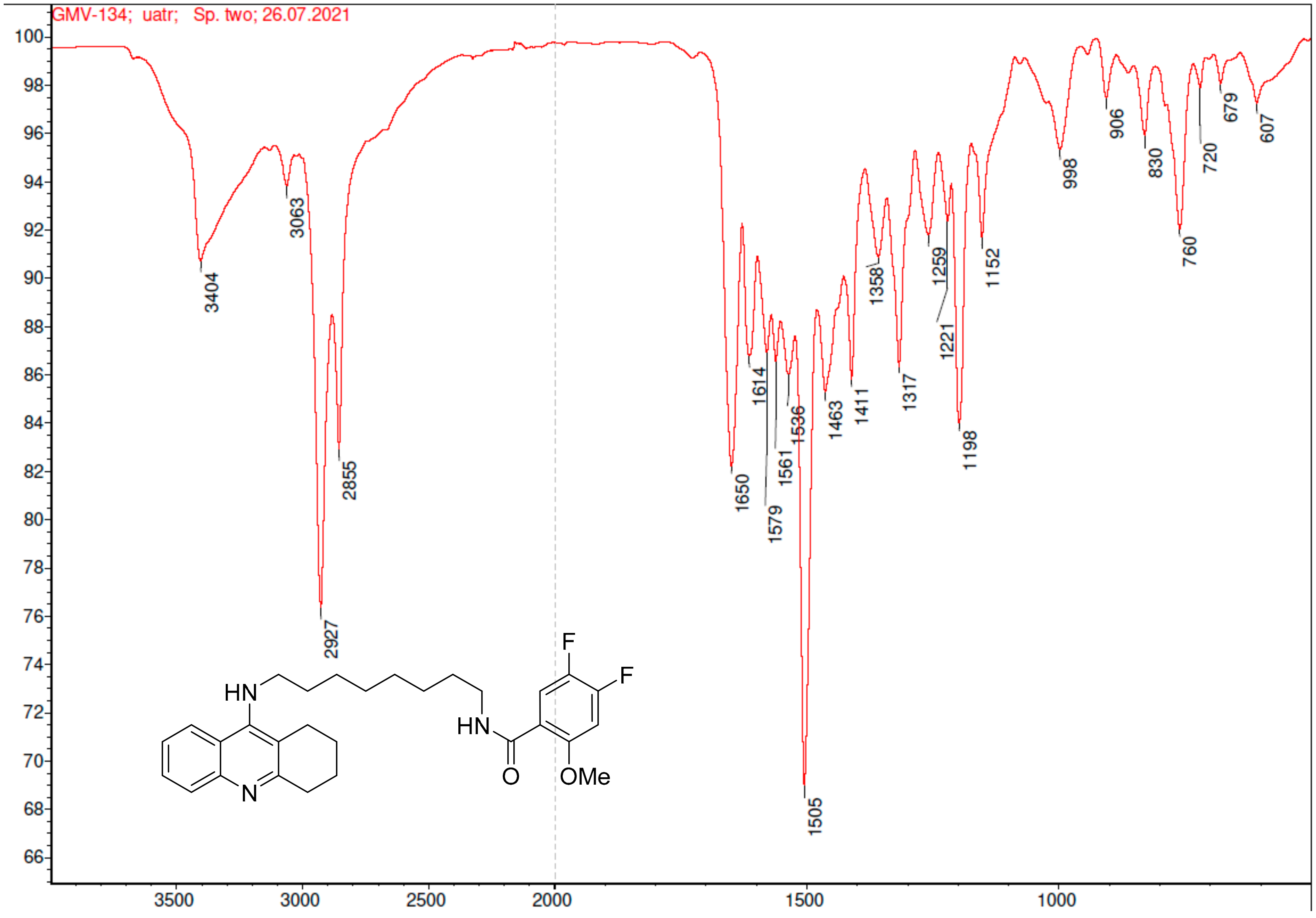

Figure S36. IR spectrum of compound **8c**

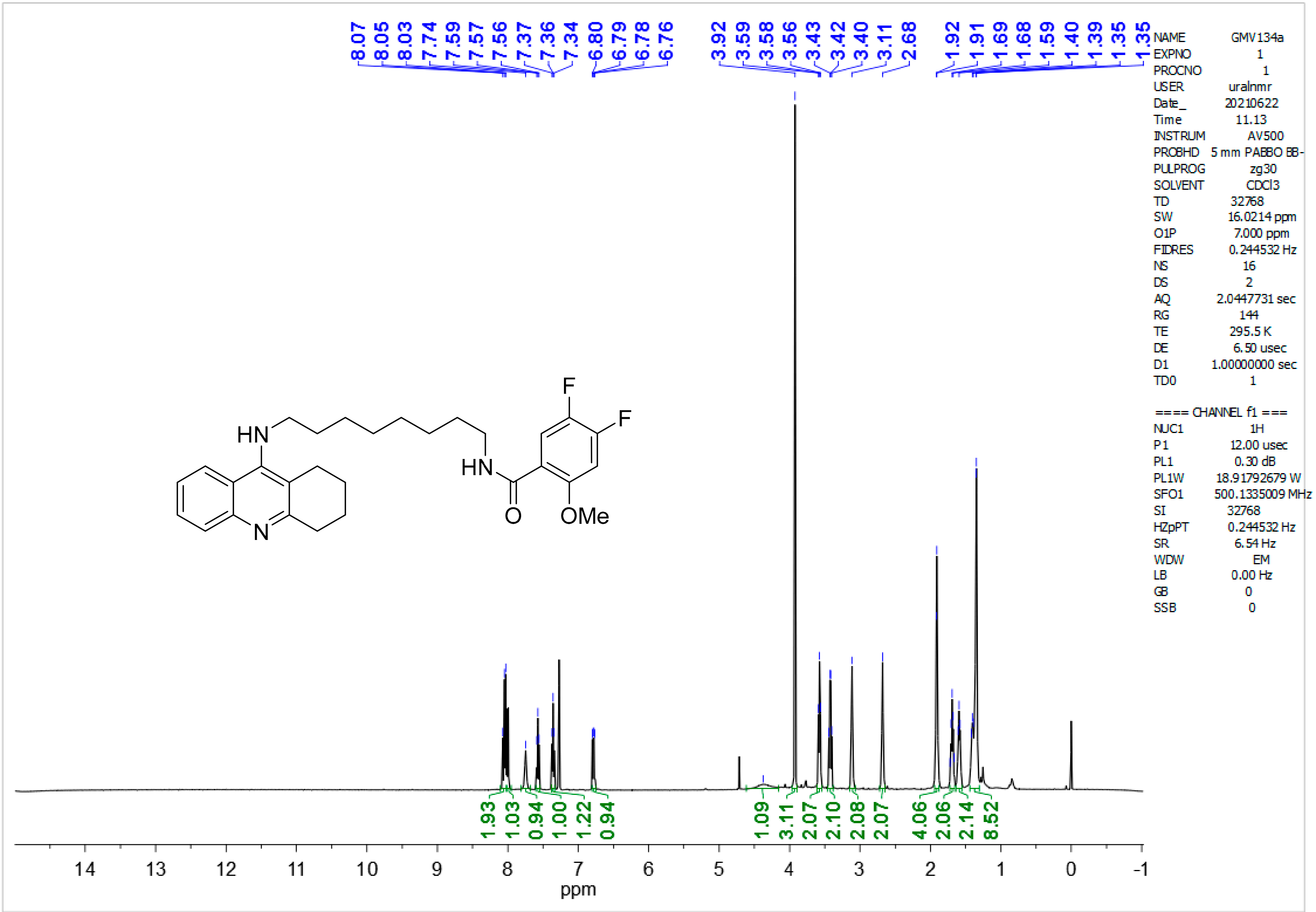

Figure S37. ^{1}H NMR spectrum of compound **8c**

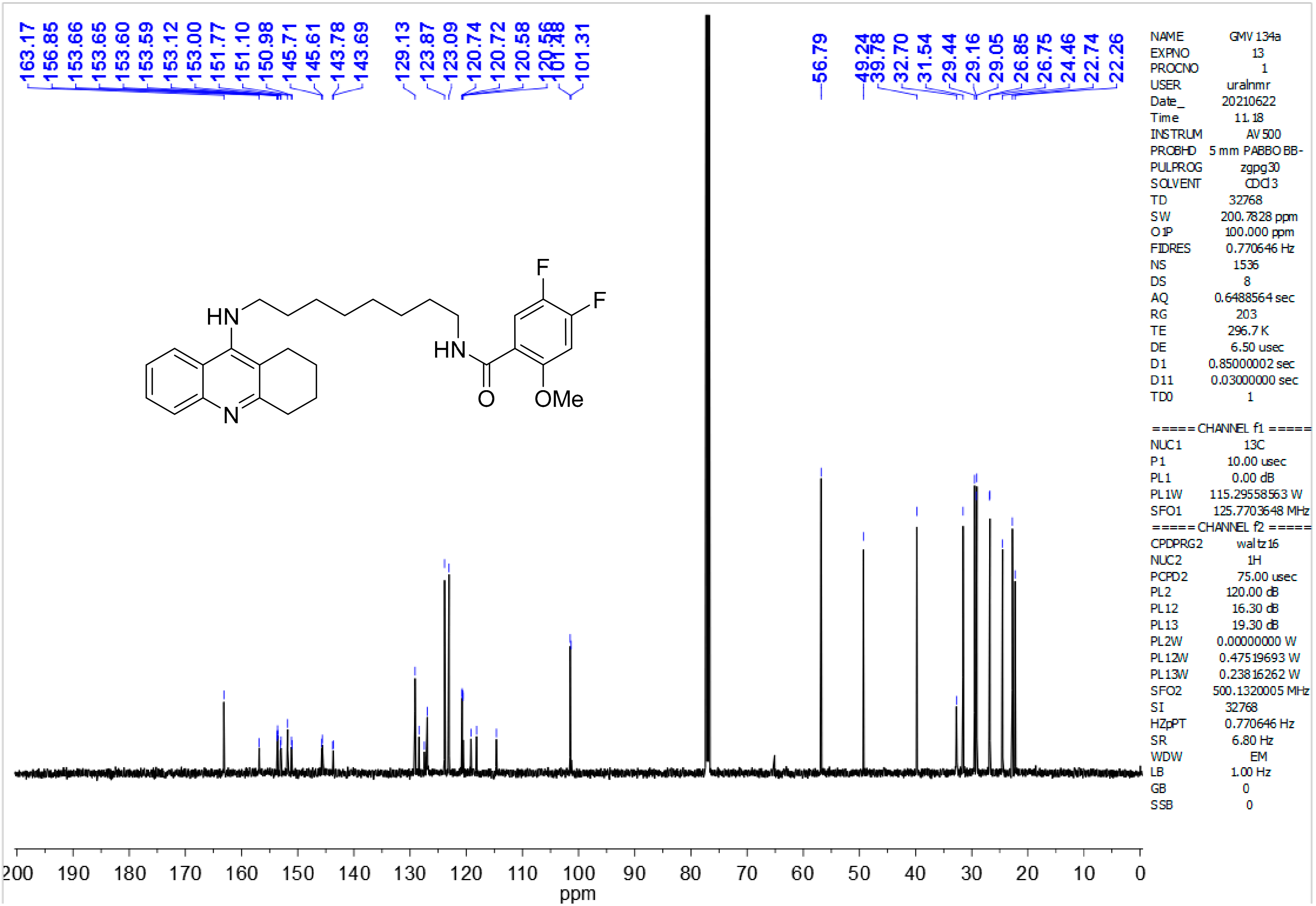

Figure S38. ^{13}C NMR spectrum of compound **8c**

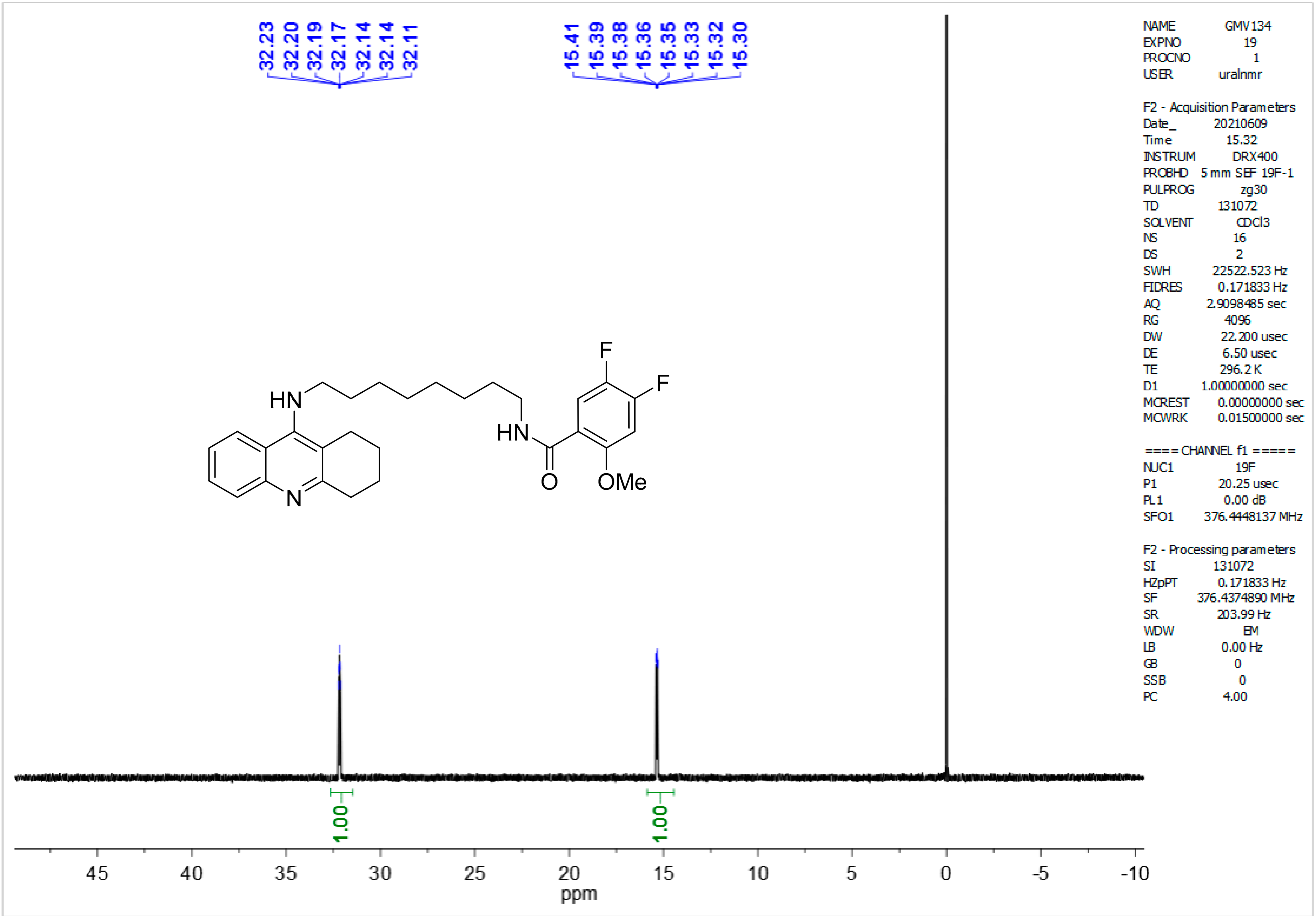

Figure S39. ^{19}F NMR spectrum of compound **8c**

Compound Spectrum SmartFormula Report

Analysis Info Acquisition Date 9/23/2021 10:46:14 AM

Analysis Name D:\Data\ING21\GMV-134.22i-C.EP180.6223_23i1045.d
Method EP180UI21HPC50-1600_500-3500-0.4-4-200_1f2002f200hrf70ie5lm70ce10pps6crf300-1200tt40-110_F3x1_Segm1.m
Sample Name Instrument maXis impact
Comment 2/09/2022: +Bckgnd: 118.09, 322.05, 622.03, 922.01, 1221.99, 1521.97, 1821.95, 2121.93, 2421.91, 2721.89 (G1969-85000; +/-299.981 HPC); other intense peaks (>2*e4): 102.13 (NEt3); 132.91 (*2-PrOH); 391.28&413.26 (DOP); 86.10, 113.13, 140.07, 149.02, 158.96, 167.03, 187.07. 194.10, 203.14, 207.17, 209.19, 214.25, 217.10, 223.21, 227.23, 237.22, 245.19, 249.22, 251.24, 255.27, 259.20, 263.23, 265.25, 273.22, 279.16, 291.27, 293.28, 304.30, 307.30, 321.31, 326.38, 332.33, 335.33, 349.35, 413.27, 1259.95, 1307.08, 1559.93: background (prev. analyzed samples and impurities); 188.09 (#6216); 588.32 (#6218); 404.23 (#6219); 376.20 (#6220); 440.21 (#6221)

Acquisition Parameter 1819696.00172

Source Type	ESI	Ion Polarity	Positive	Set Nebulizer	0.4 Bar
Focus	Active	Set Capillary	3500 V	Set Dry Heater	200 °C
Scan Begin	50 m/z	Set End Plate Offset	-500 V	Set Dry Gas	4.0 l/min
Scan End	1600 m/z	Set Charging Voltage	2000 V	Set Divert Valve	Source
		Set Corona	0 nA	Set APCI Heater	0 °C

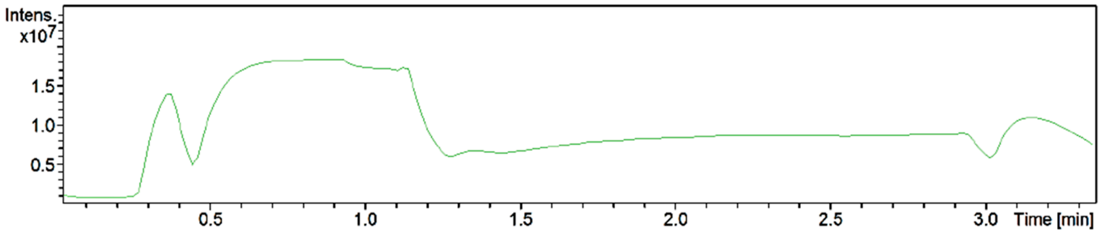

+MS, 0.3-0.4min #18-22

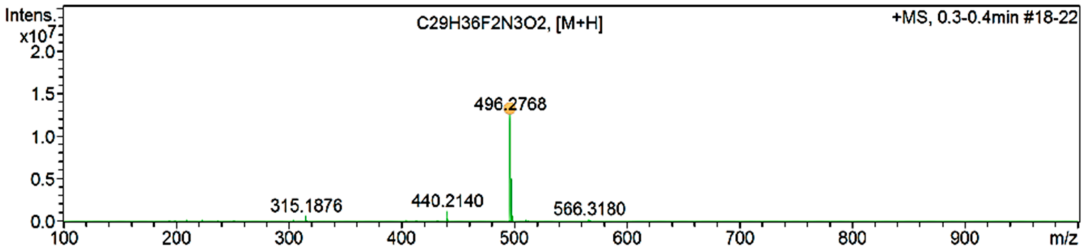

Meas. m/z	#	Ion Formula	m/z	err [ppm]	mSigma	# mSigma	Score	rdb	e¯ Conf	N-Rule
496.2768	1	C32H35FN3O	496.2759	-1.9	21.6	1	100.00	16.5	even	ok
	2	C29H36F2N3O2	496.2770	0.4	42.7	2	64.33	12.5	even	ok

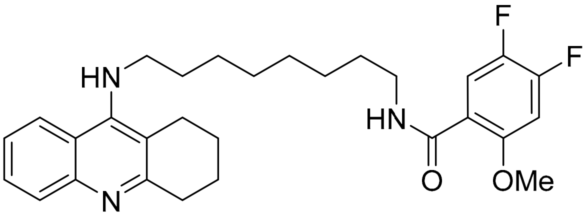

GMV-134.22i-C.EP180.6223_23i1045.d
Bruker Compass DataAnalysis 4.2 printed: 9/23/2021 10:51:04 AM Page 1 of 1

Figure S40. HRMS spectrum of compound **8c**

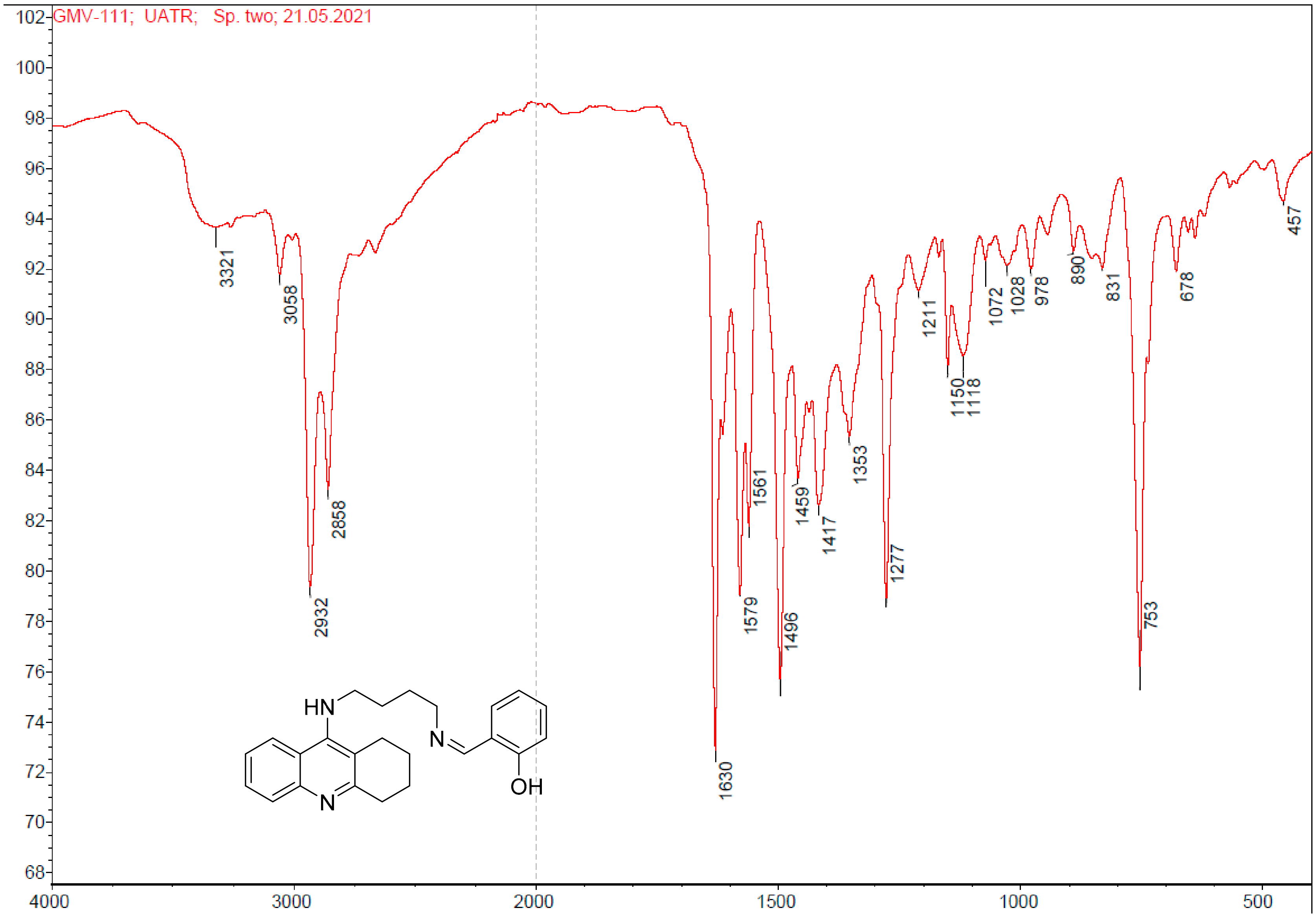

Figure S41. IR spectrum of compound **10a**

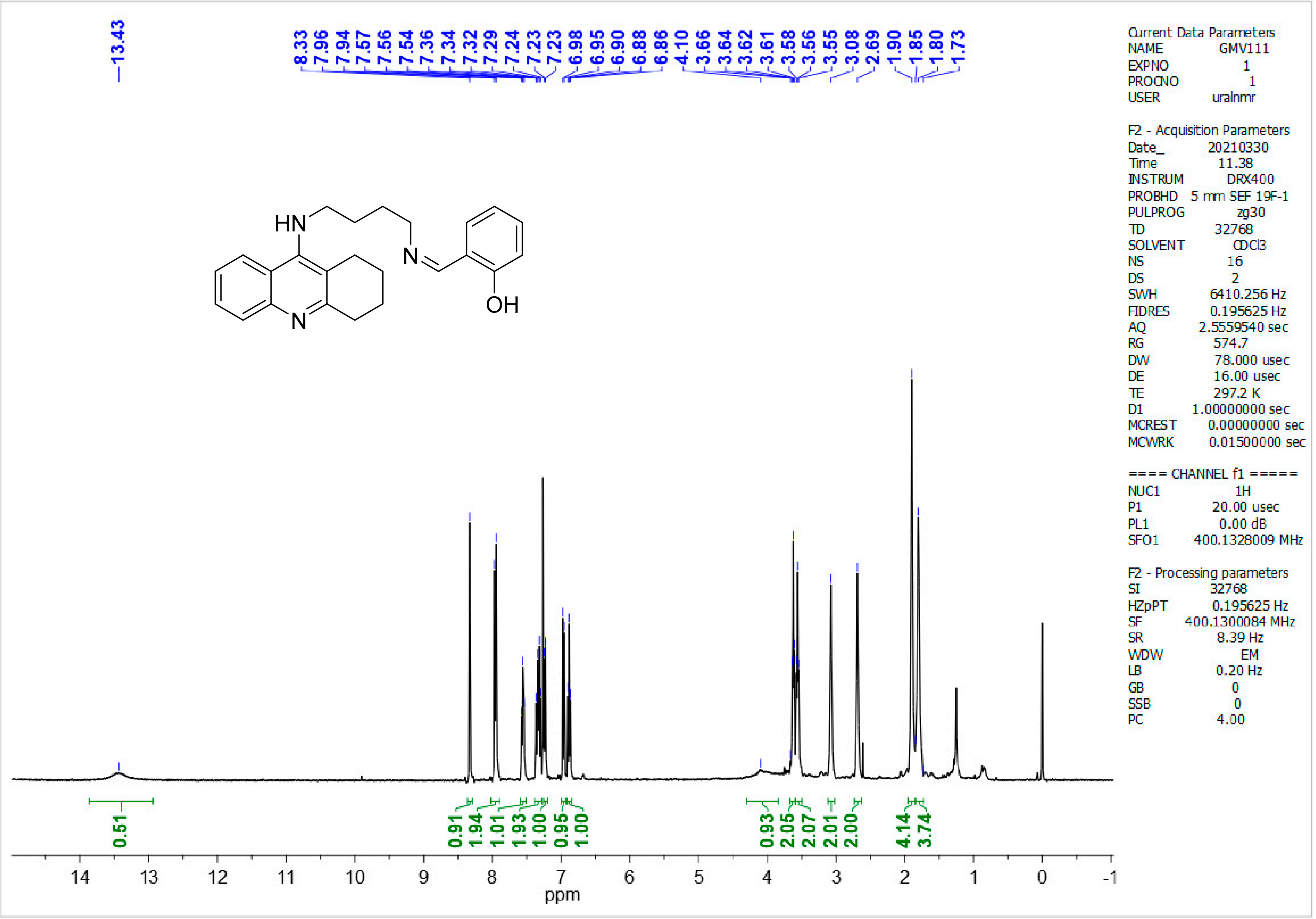

Figure S42. ^{1}H NMR spectrum of compound **10a**

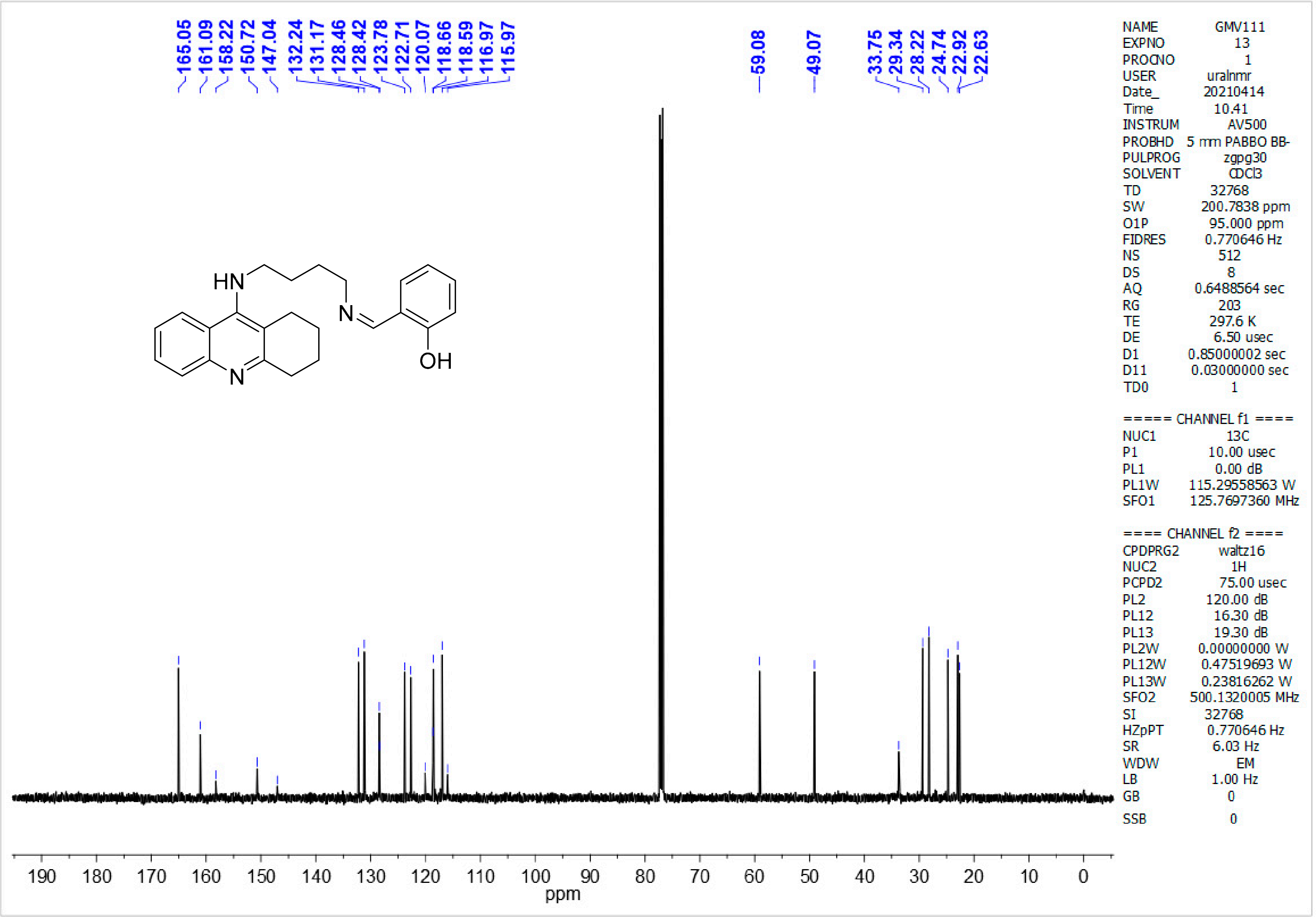

Figure S43. ^{13}C NMR spectrum of compound **10a**

Compound Spectrum SmartFormula Report

Analysis Info

Analysis Name	D:\Data\ING21\GMV-111.22D-C1.6-ESIPOS-180.5875-26D1615.d	Acquisition Date	4/26/2021 4:17:07 PM
Method	EP180_50-2200_TunePosStd-UA13_1f3002f200hrf50ie3lm1 00ce3crf300-800tt60-120pps6x0.75_fsthpc.m	Operator	admin
Sample Name		Instrument	maXis impact 1819696.00172
Comment	26/04/2021: +Bckgnd: 118.09, 322.05, 622.03, 922.01, 1221.99, 1521.97, 1821.95, 2121.93, 2421.91, 2721.89 (G1969-85000; +/-299.981 HPC); other intense peaks (>2e4): 102.13 (NEt3); 132.91 (2-PrOH); 391.28 (DOP); 79.0, 86.10, 111,09, 157.03 (DMSO); 129.05, 132.91, 144.98, 140.02, 161.08, 165.13, 175.10, 183.17, 194.12, 199.12, 209.19, 214.25, 223.21, 227.24, 237.24, 249.22, 251.24, 251.24, 255.27, 277.25, 293.28, 299.19, 304.30, 307.30, 344.19, 407.10, 430.17, 707.12, 1007.10, 1307.08, 1557.95: background (prev. analyzed samples and impurities); 339.12 (#5451); 446.28 (#5721); 390.22 (#5874); 430.29 (#5876); 419.25 (#5878); 402.25 (#5876)		

Acquisition Parameter

Source Type	ESI	Ion Polarity	Positive	Set Nebulizer	0.3 Bar
Focus	Active	Set Capillary	3500 V	Set Dry Heater	200 °C
Scan Begin	50 m/z	Set End Plate Offset	-500 V	Set Dry Gas	4.0 l/min
Scan End	2200 m/z	Set Charging Voltage	2000 V	Set Divert Valve	Source
		Set Corona	0 nA	Set APCI Heater	0 °C

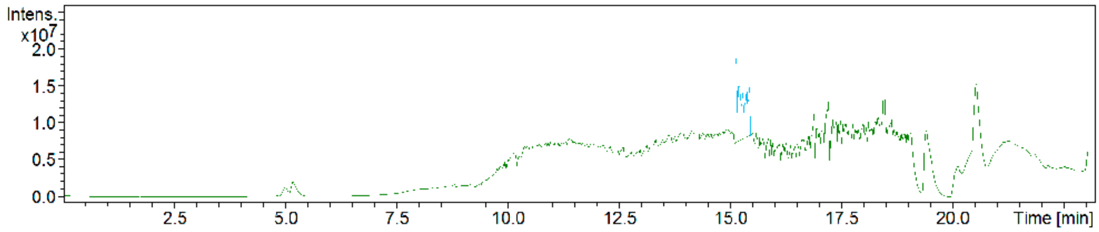

+MS, 20.3-22.9min #886-996 (i.EQ; -322)

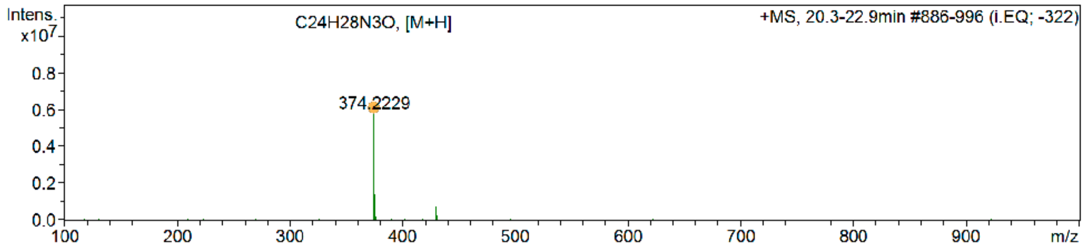

Meas. m/z	#	Ion Formula	m/z	err [ppm]	mSigma	# mSigma	Score	rdb	e⁻ Conf	N-Rule
374.2229	1	C24H28N3O	374.2227	-0.7	14.4	1	100.00	12.5	even	ok

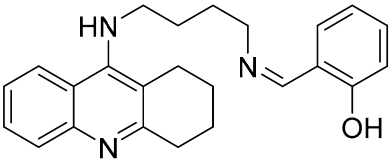

GMV-111.22D-C1.6-ESIPOS-180.5875-26D1615.d

Bruker Compass DataAnalysis 4.2 printed: 4/26/2021 4:45:50 PM by: admin Page 1 of 1

Institute of Organic Synthesis UB RAS
22 S.Kovalevskoy, 20 Akademicheskaya str, Yekaterinburg, Russian Federation
Phone: +7 (343) 362-34-56

Figure S44. HRMS spectrum of compound **10a**

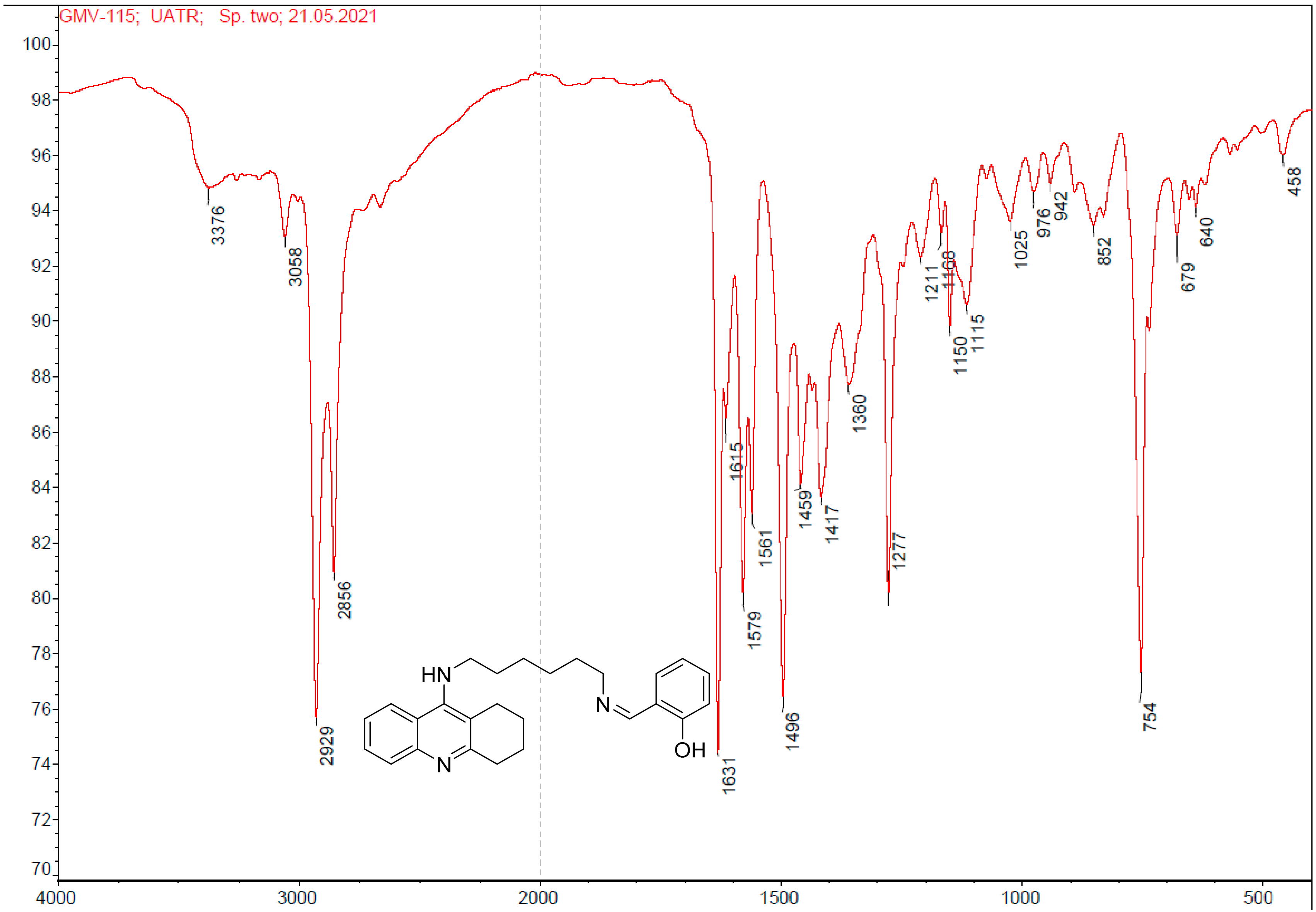

Figure S45. IR spectrum of compound **10b**

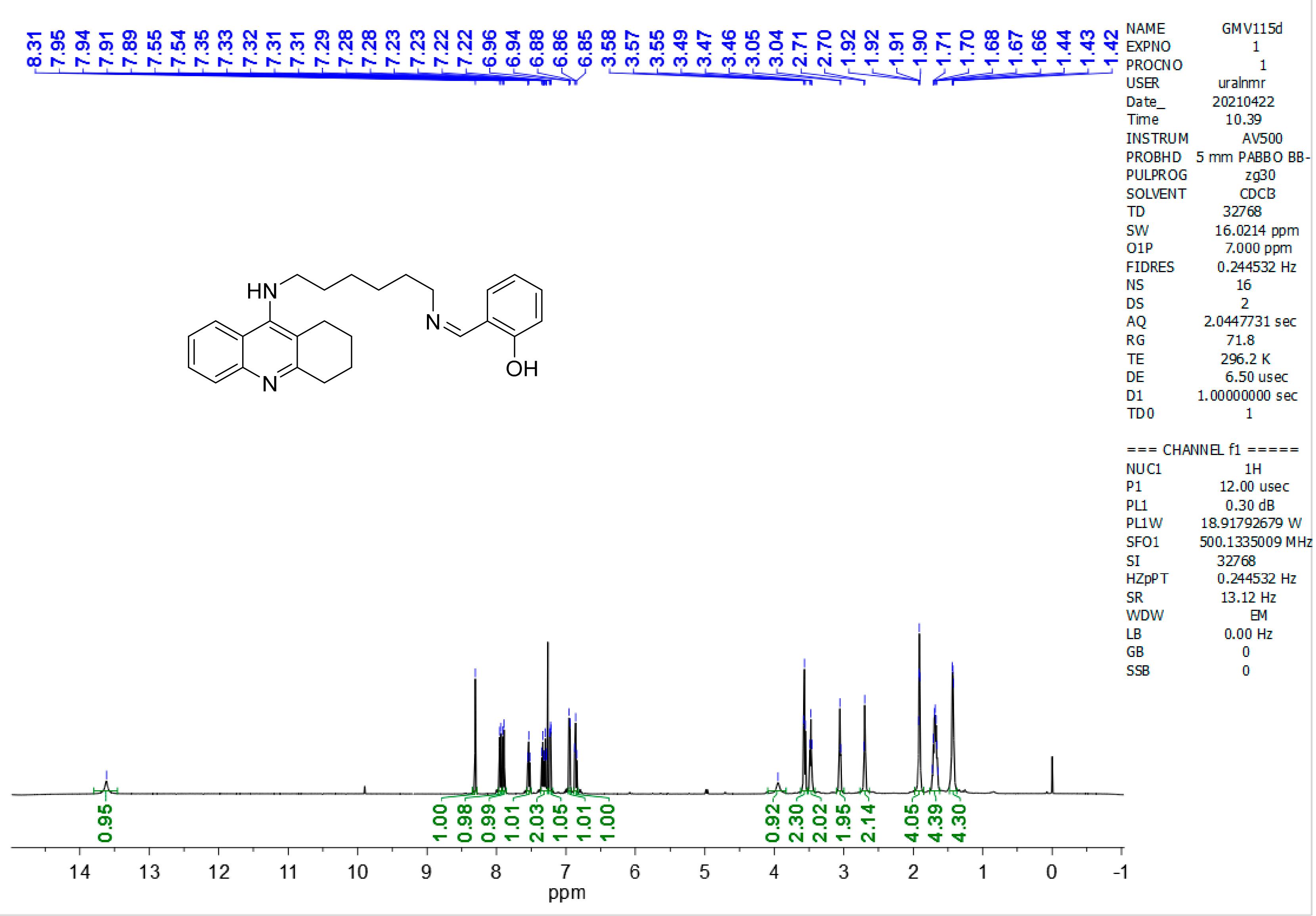

Figure S46. ^{1}H NMR spectrum of compound **10b**

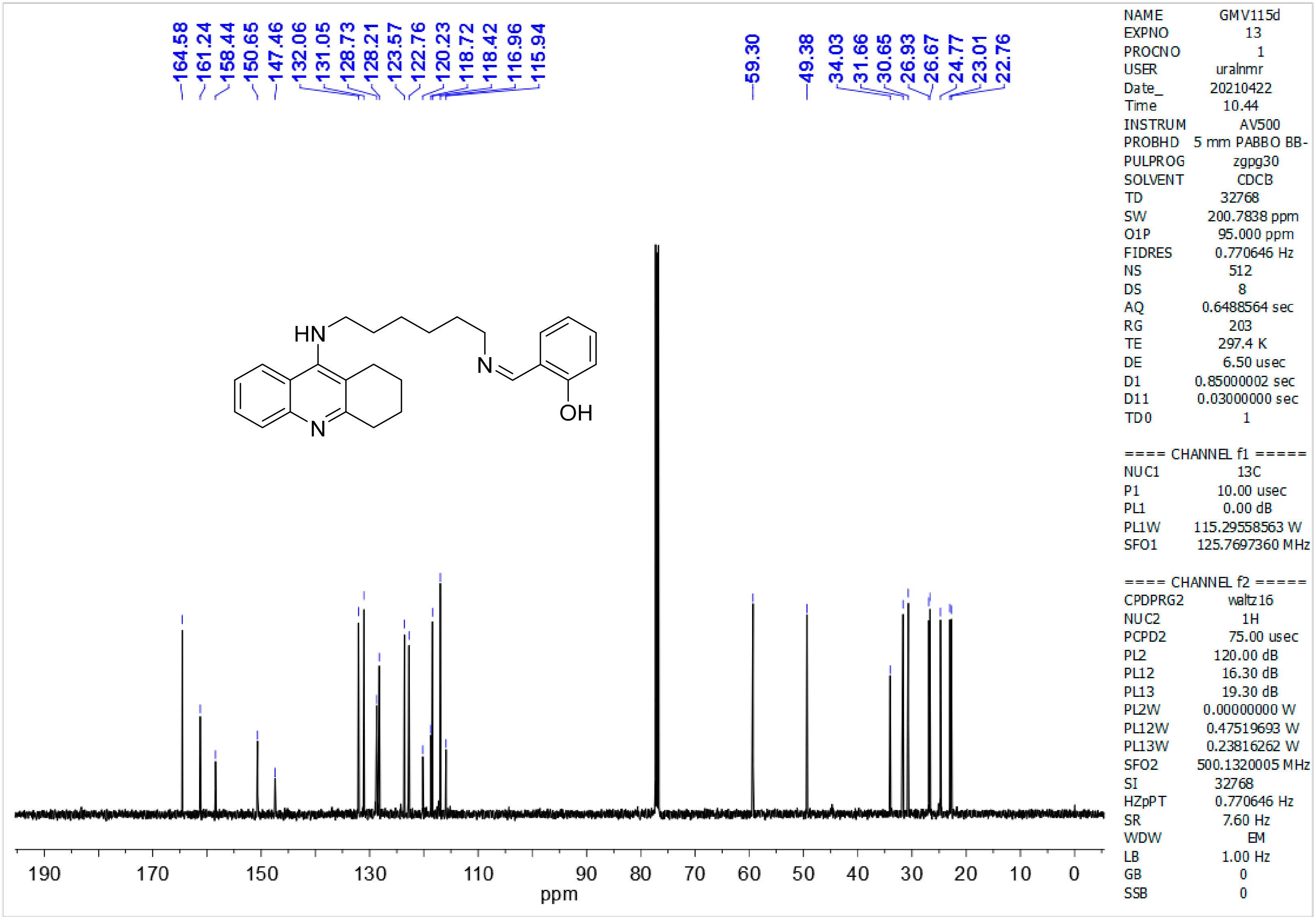

Figure S47. ^{13}C NMR spectrum of compound **10b**

Compound Spectrum SmartFormula Report

Analysis Info

Acquisition Date 4/26/2021 3:41:41 PM

Analysis Name D:\Data\ING21\GMV-115.22D-C1.6-ESIPOS-180.5876-26D1540.d

Method EP180_50-2200_TunePosStd-UA13_1f3002f200hrf50ie3lm1 00ce3crf300-800tt60-120pps6x0.75_fsthpc.m

Operator admin

Sample Name

Instrument maXis impact 1819696.00172

Comment 26/04/2021: +Bckgnd: 118.09, 322.05, 622.03, 922.01, 1221.99, 1521.97, 1821.95, 2121.93, 2421.91, 2721.89 (G1969-85000; +/-299.981 HPC); other intense peaks (>2e4): 102.13 (NEt3); 132.91 (2-PrOH); 391.28 (DOP); 79.0, 86.10, 111,09, 157.03 (DMSO); 129.05, 132.91, 144.98, 140.02, 161.08, 165.13, 175.10, 183.17, 194.12, 199.12, 209.19, 214.25, 223.21, 227.24, 237.24, 249.22, 251.24, 251.24, 255.27, 277.25, 293.28, 299.19, 304.30, 307.30, 344.19, 407.10, 430.17, 707.12, 1007.10, 1307.08, 1557.95: background (prev. analyzed samples and impurities); 339.12 (#5451); 446.28 (#5721); 390.22 (#5874); 430.29 (#5876); 419.25 (#5878)

Acquisition Parameter

Source Type	ESI	Ion Polarity	Positive	Set Nebulizer	0.3 Bar
Focus	Active	Set Capillary	3500 V	Set Dry Heater	200 °C
Scan Begin	50 m/z	Set End Plate Offset	-500 V	Set Dry Gas	4.0 l/min
Scan End	2200 m/z	Set Charging Voltage	2000 V	Set Divert Valve	Source
		Set Corona	0 nA	Set APCI Heater	0 °C

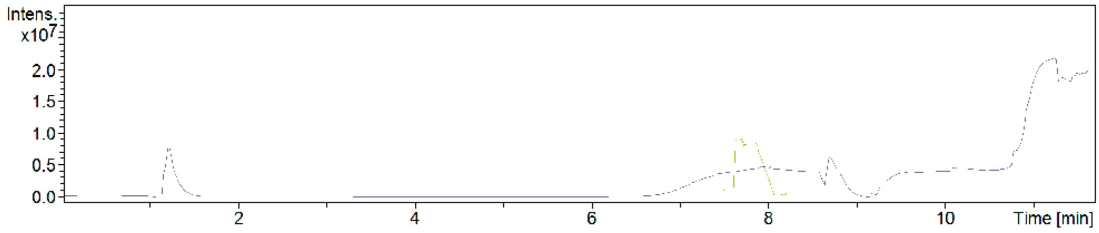

+MS, 6.9-7.4min #302-324

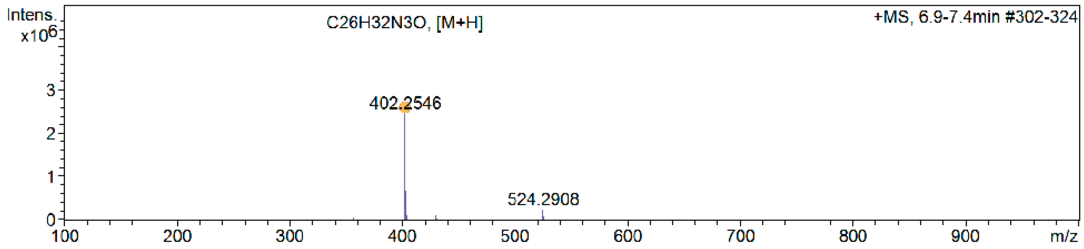

Meas. m/z	#	Ion Formula	m/z	err [ppm]	mSigma	# mSigma	Score	rdb	e⁻ Conf	N-Rule
402.2546	1	C26H32N3O	402.2540	-1.6	13.4	1	100.00	12.5	even	ok

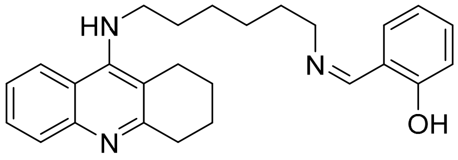

Institute of Organic Synthesis UB RAS
22 S.Kovalevskoy, 20 Akademicheskaya str, Yekaterinburg, Russian Federation
Phone: +7 (343) 362-34-56

Figure S48. HMRS spectrum of compound **10b**

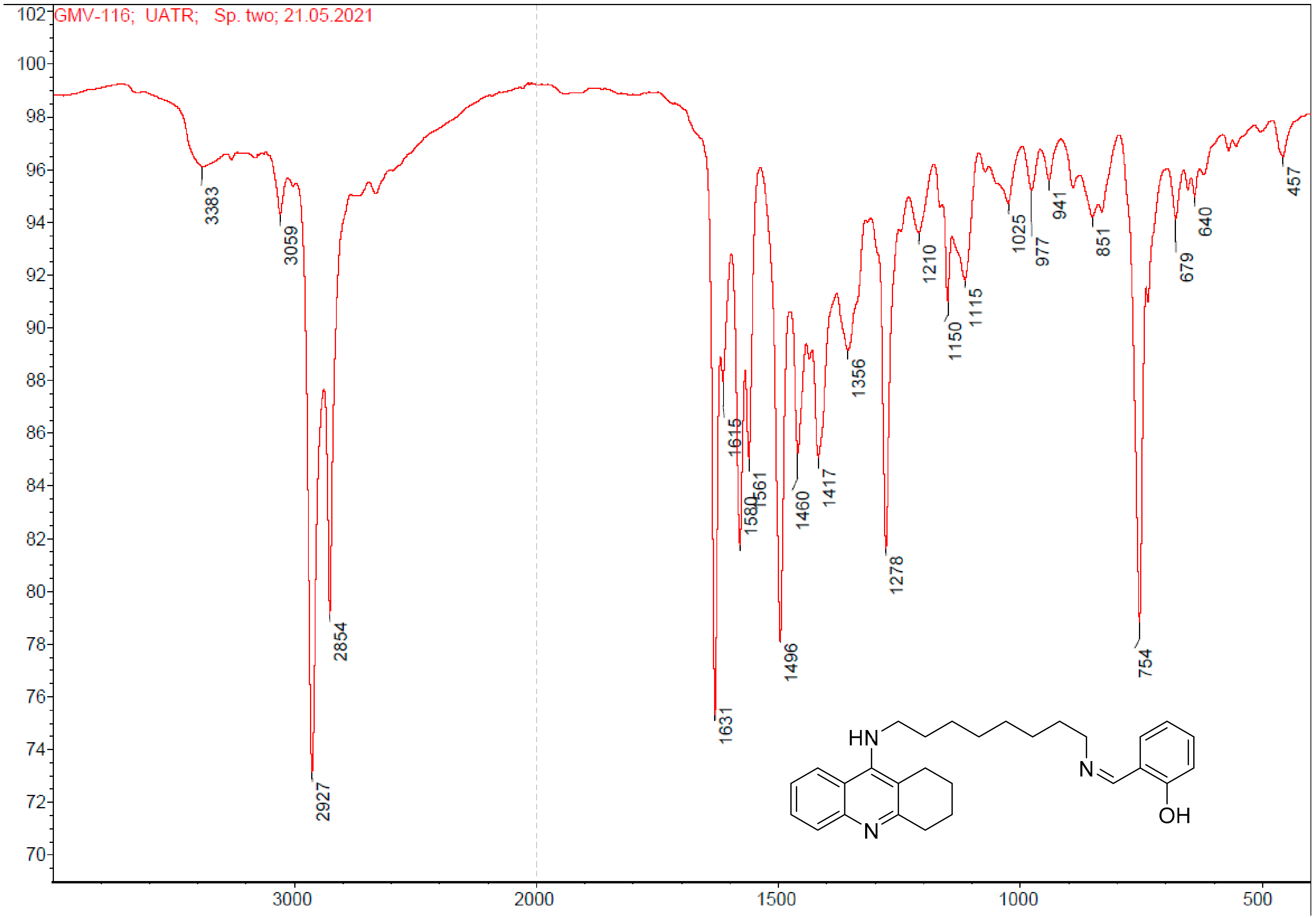

Figure S49. IR spectrum of compound **10c**

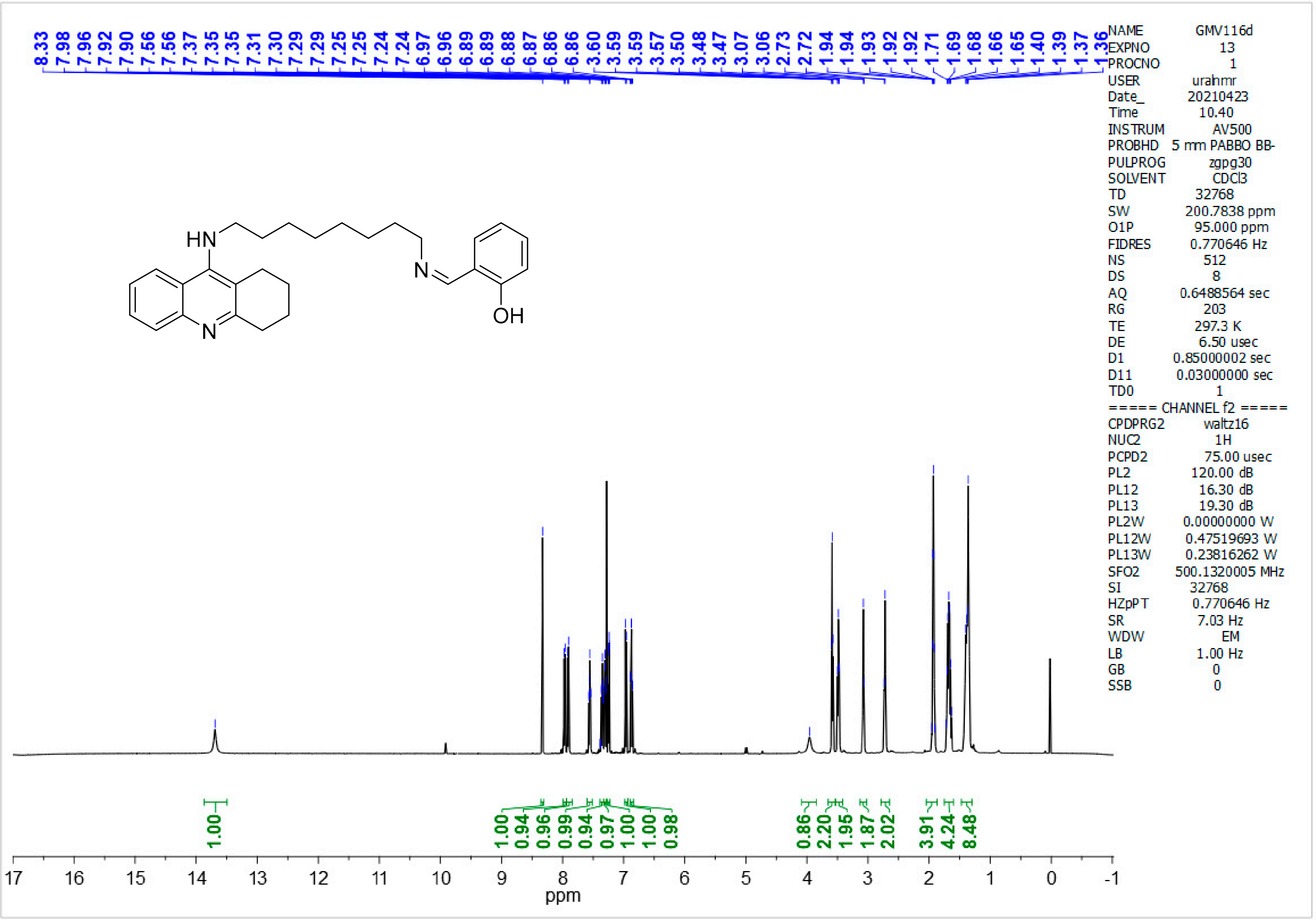

Figure S50. ^{1}H NMR spectrum of compound **10c**

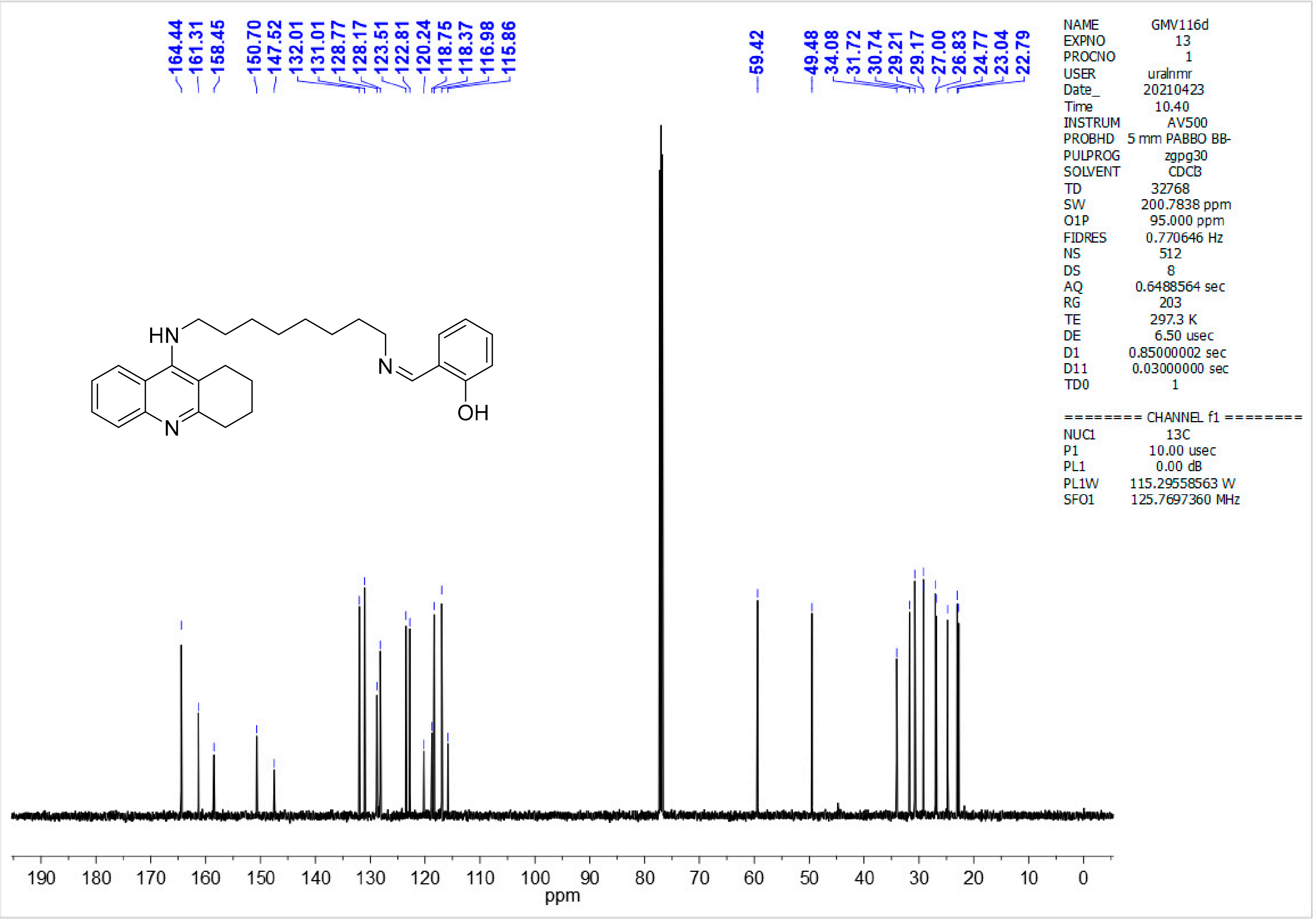

Figure S51. ^{13}C NMR spectrum of compound **10c**

Compound Spectrum SmartFormula Report

Analysis Info

Acquisition Date	4/26/2021 2:59:56 PM
Analysis Name	D:\Data\ING21\GMV-116.22D-C1.6-ESIPOS-180.5877-26D1500.d
Method	EP180_50-2200_TunePosStd-UA13_1f3002f200hrf50ie3lm1 00ce3crf300-800tt60-120pps6x0.75_fsthpc.m
Operator	admin
Sample Name	
Instrument	maXis impact 1819696.00172
Comment	26/04/2021: +Bckgnd: 118.09, 322.05, 622.03, 922.01, 1221.99, 1521.97, 1821.95, 2121.93, 2421.91, 2721.89 (G1969-85000; +/-299.981 HPC); other intense peaks (>2e4): 102.13 (NEt3); 132.91 (2-PrOH); 391.28 (DOP); 79.0, 86.10, 111,09, 157.03 (DMSO); 129.05, 132.91, 144.98, 140.02, 161.08, 165.13, 175.10, 183.17, 194.12, 199.12, 209.19, 214.25, 223.21, 227.24, 237.24, 249.22, 251.24, 251.24, 255.27, 277.25, 293.28, 299.19, 304.30, 307.30, 344.19, 407.10, 430.17, 707.12, 1007.10, 1307.08, 1557.95: background (prev. analyzed samples and impurities); 339.12 (#5451); 446.28 (#5721); 309.02/311.02 (#5899); 155.08 (#5898); 231.11 (#5901); 321.14 (#5990); 367,19 (#5873); 390.22 (#5874)

Acquisition Parameter

Source Type	ESI	Ion Polarity	Positive	Set Nebulizer	0.3 Bar
Focus	Active	Set Capillary	3500 V	Set Dry Heater	200 °C
Scan Begin	50 m/z	Set End Plate Offset	-500 V	Set Dry Gas	4.0 l/min
Scan End	2200 m/z	Set Charging Voltage	2000 V	Set Divert Valve	Source
		Set Corona	0 nA	Set APCI Heater	0 °C

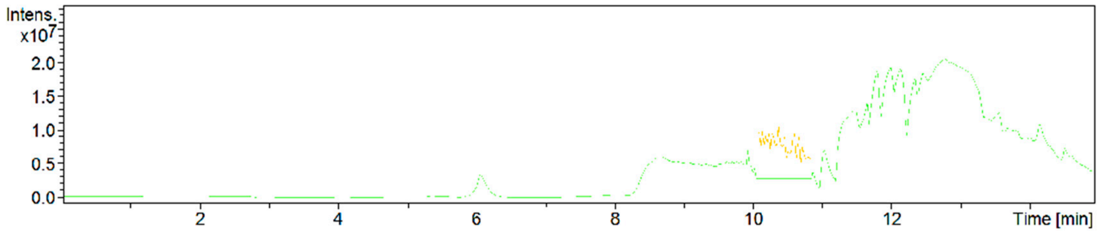

+MS, 13.6-14.9min #592-648

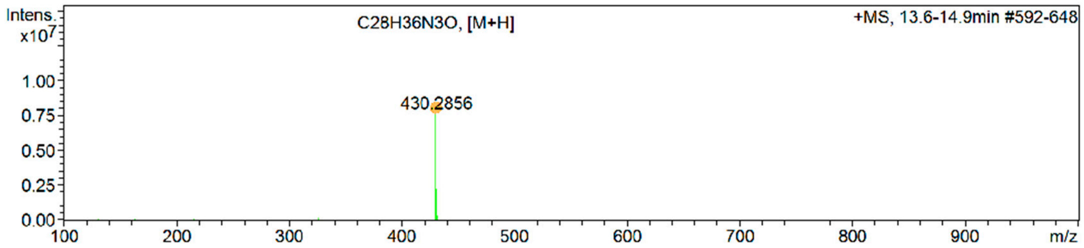

Meas. m/z	#	Ion Formula	m/z	err [ppm]	mSigma	# mSigma	Score	rdb	e⁻ Conf	N-Rule
430.2856	1	C28H36N3O	430.2853	-0.8	14.3	1	100.00	12.5	even	ok

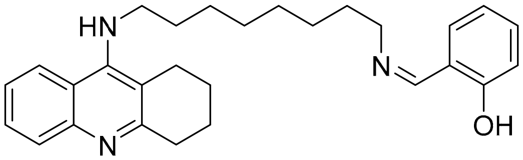

Institute of Organic Synthesis UB RAS
22 S.Kovalevskoy, 20 Akademicheskaya str, Yekaterinburg, Russian Federation
Phone: +7 (343) 362-34-56

Figure S52. HRMS spectrum of compound **10c**

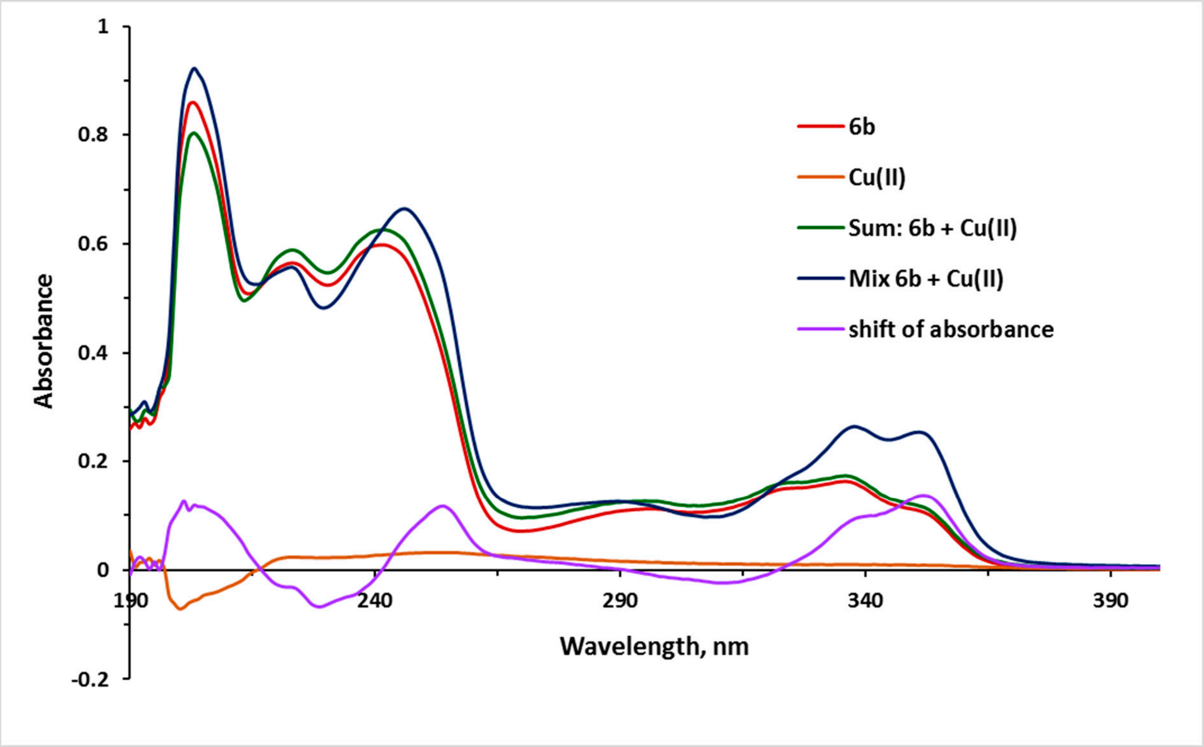

Figure S53. Absorption spectra of: compound **6b**, Cu^{2+} ions solution, a sum of **6b** and Cu^{2+}, their mixture, and the shift of the spectra caused by the formation of a complex.

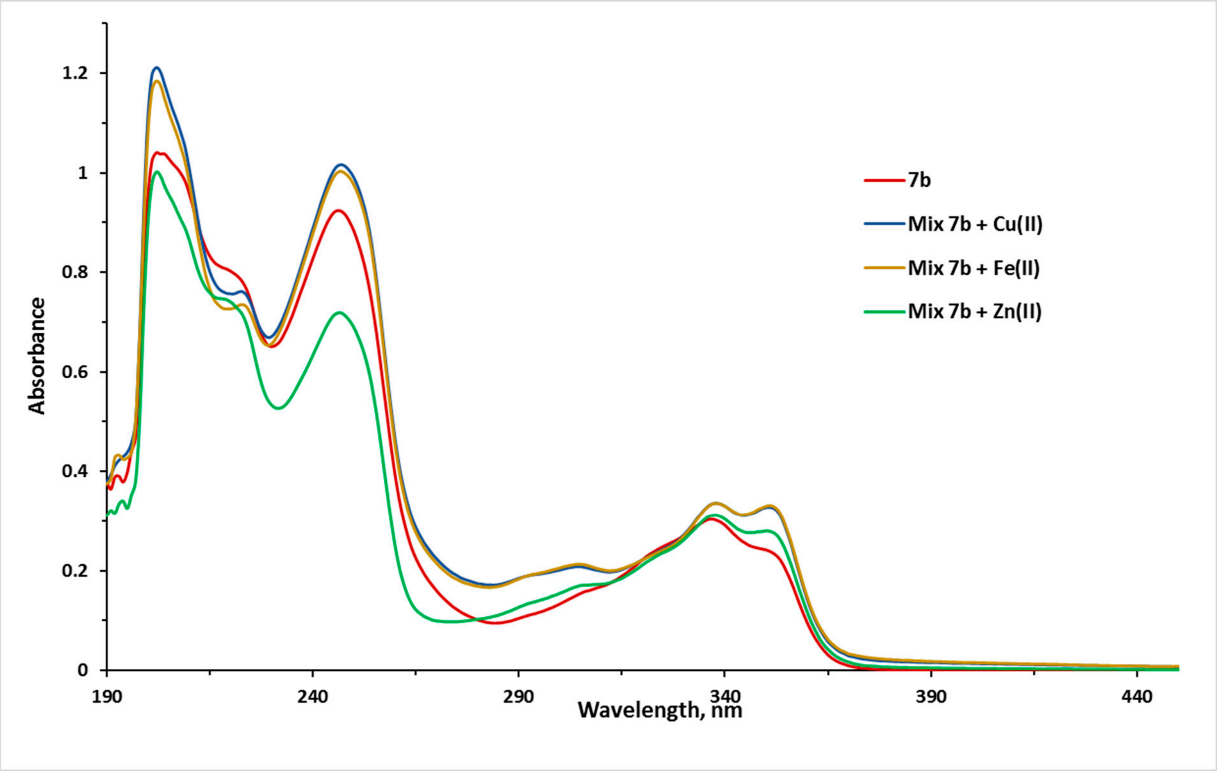

Figure S54. UV spectra of compound **7b** and mixture of **7b** with Cu(II), Fe(II) and Zn(II) ions.

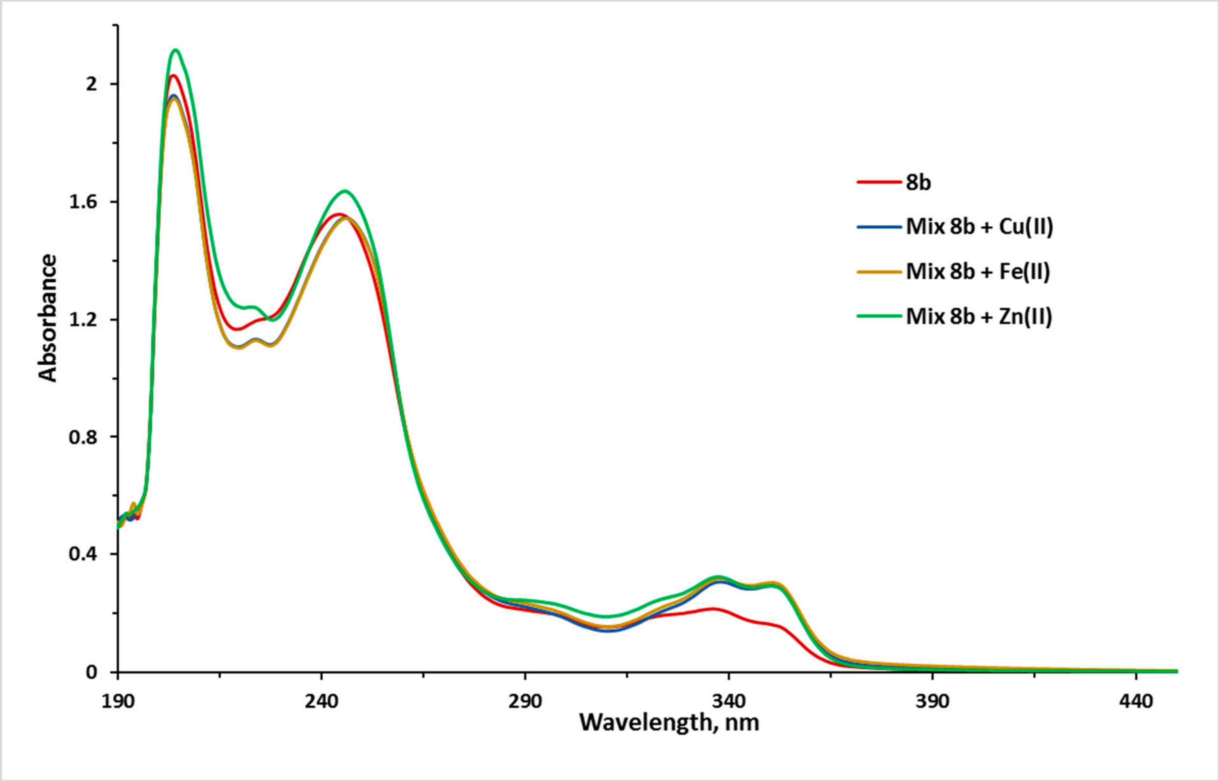

Figure S55. UV spectra of compound **8b** and mixture of **8b** with Cu(II), Fe(II) and Zn(II) ions.

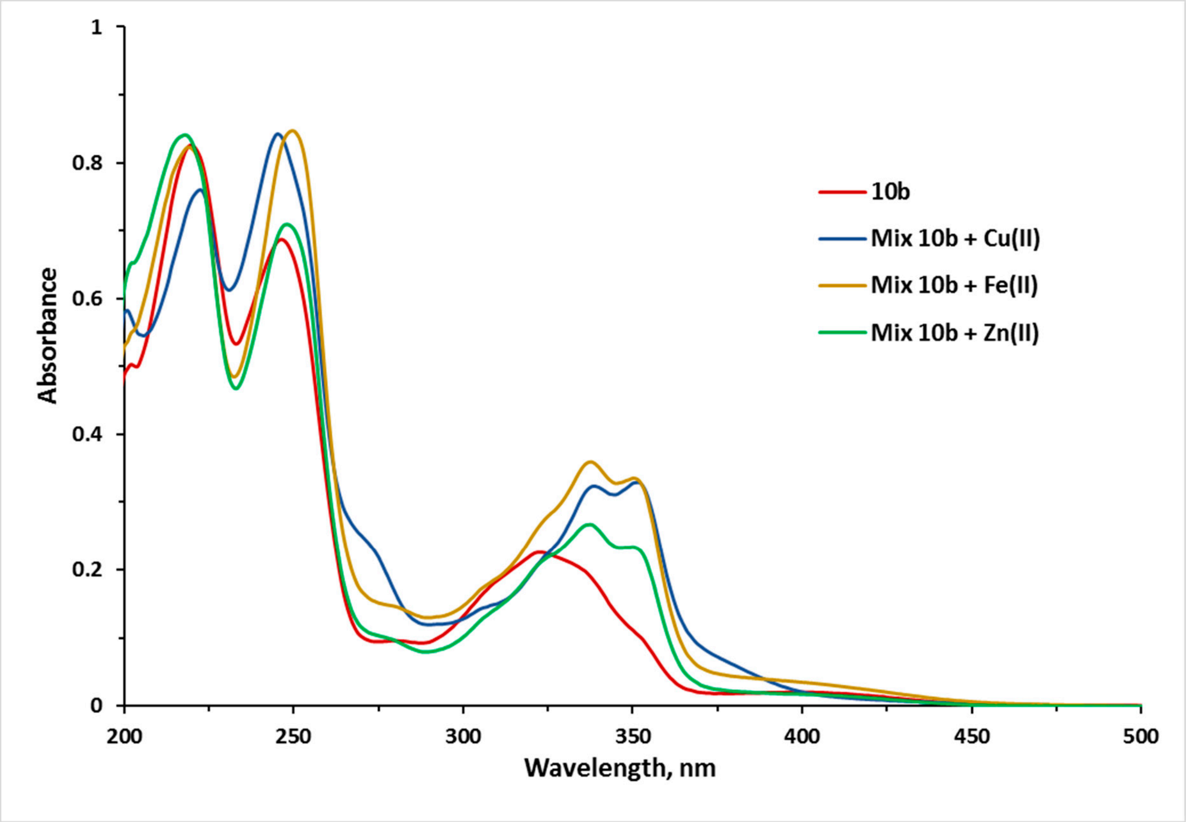

Figure S56. UV spectra of compound **10b** and mixture of **10b** with Cu(II), Fe(II) and Zn(II) ions.

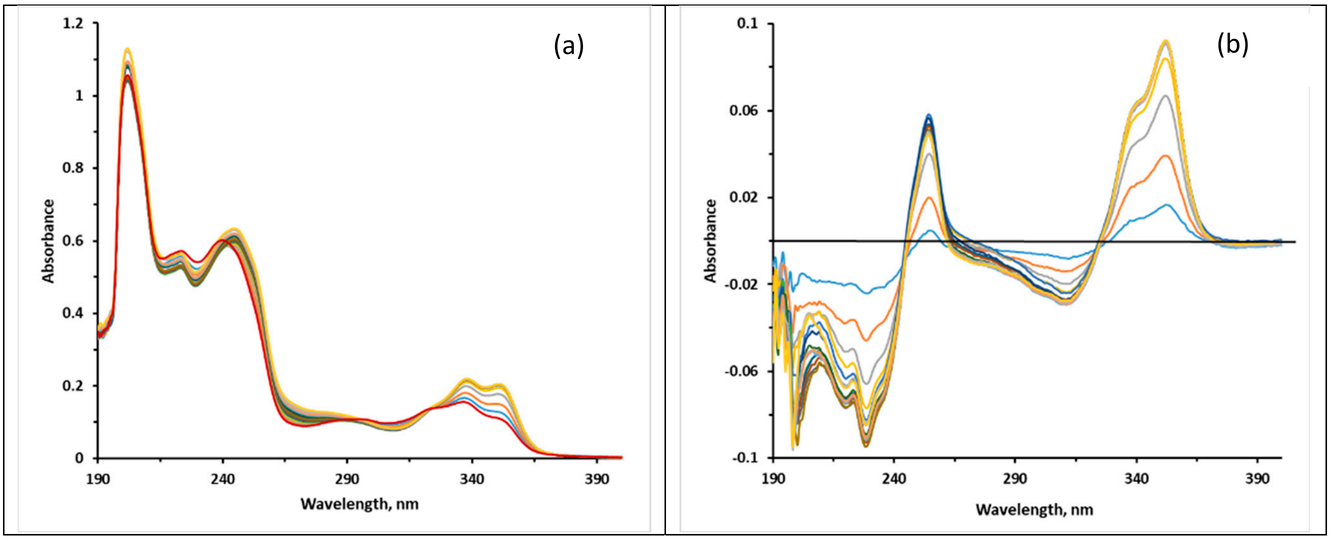

Figure S57. (a) UV absorption spectra of **6b** (20 μM) in ethanol after addition of increasing concentrations of $CuCl_2$ (2-34 μM). **(b)** the differential spectra due to 6b-Cu^{2+} complex formation obtained by numerical subtraction from the spectra of the mixture of the spectra of the Cu^{2+} alone and 6b alone at the corresponding concentrations.

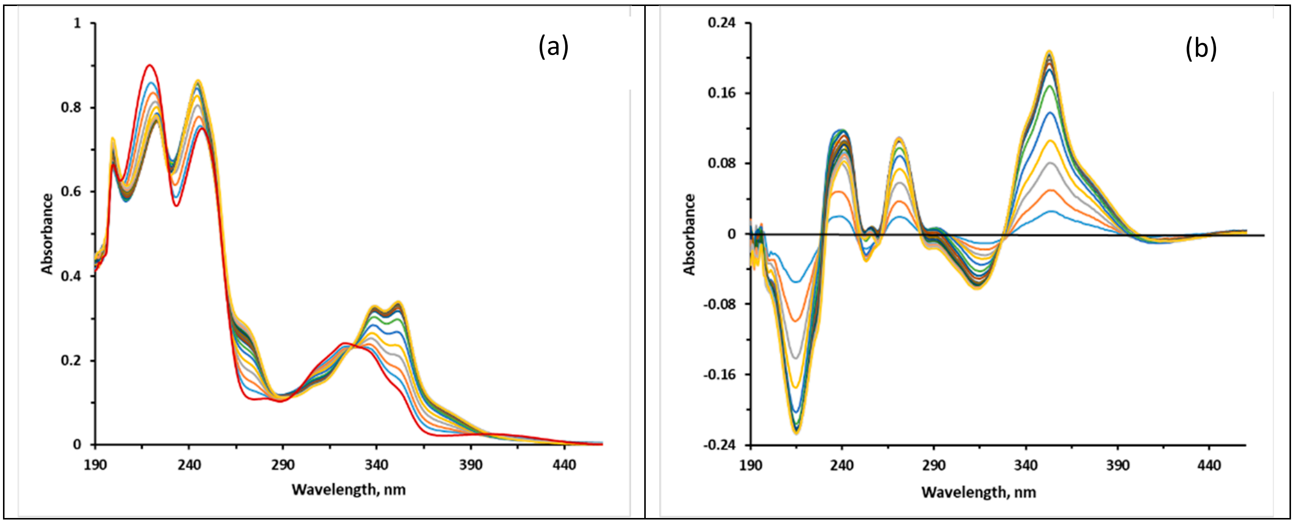

Figure S58. **(a)** UV absorption spectra of **10b** (20 μM) in ethanol after addition of increasing concentrations of $CuCl_2$ (2-34 μM). **(b)** the differential spectra due to **10b**-Cu^{2+} complex formation obtained by numerical subtraction from the spectra of the mixture of the spectra of the Cu^{2+} alone and **10b** alone at the corresponding concentrations.

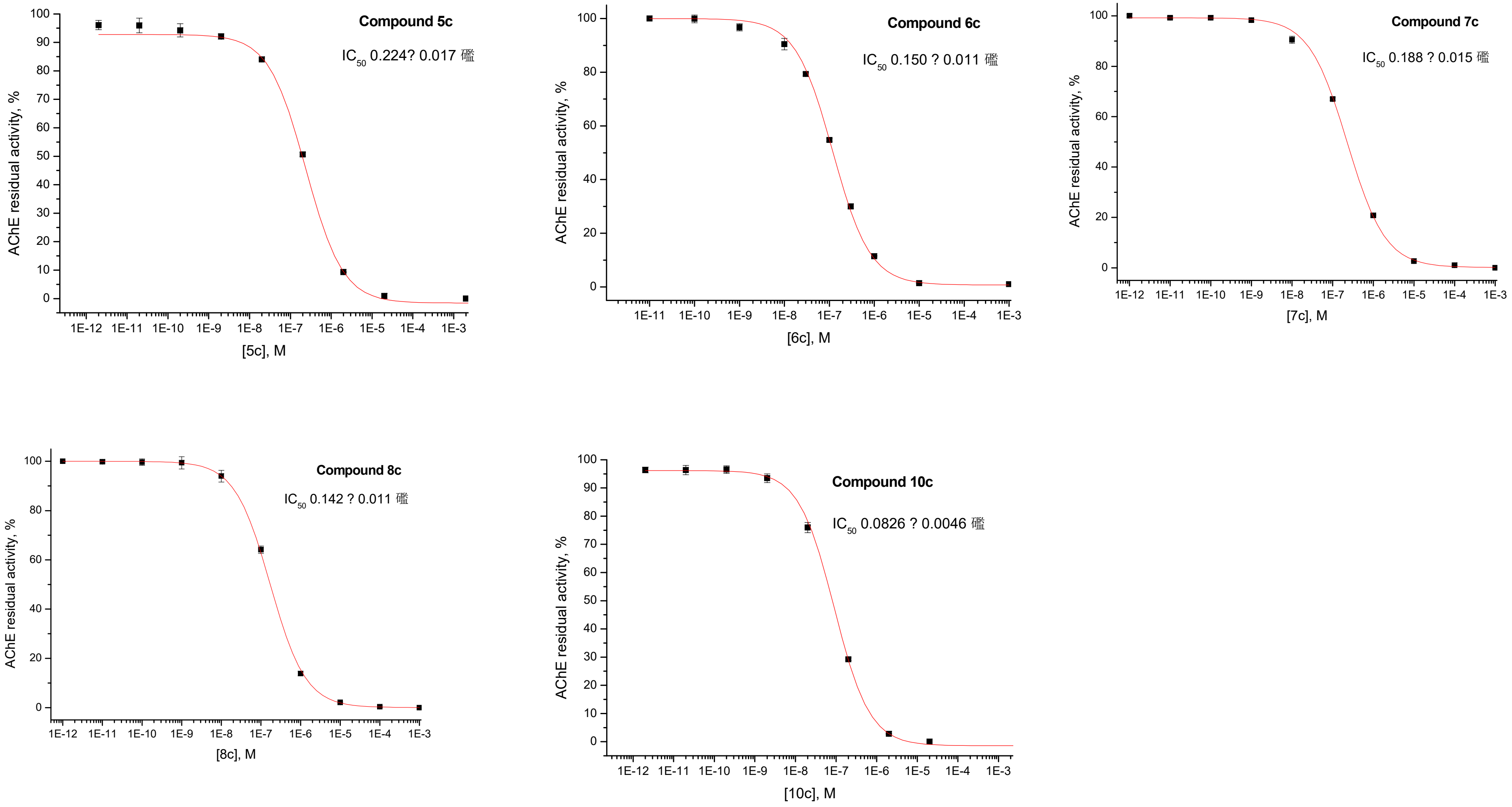

Figure S59. IC_{50} values for AChE inhibition by compounds **5c,6c,7c,8c and 10c** (Mean ± SEM, n = 3)

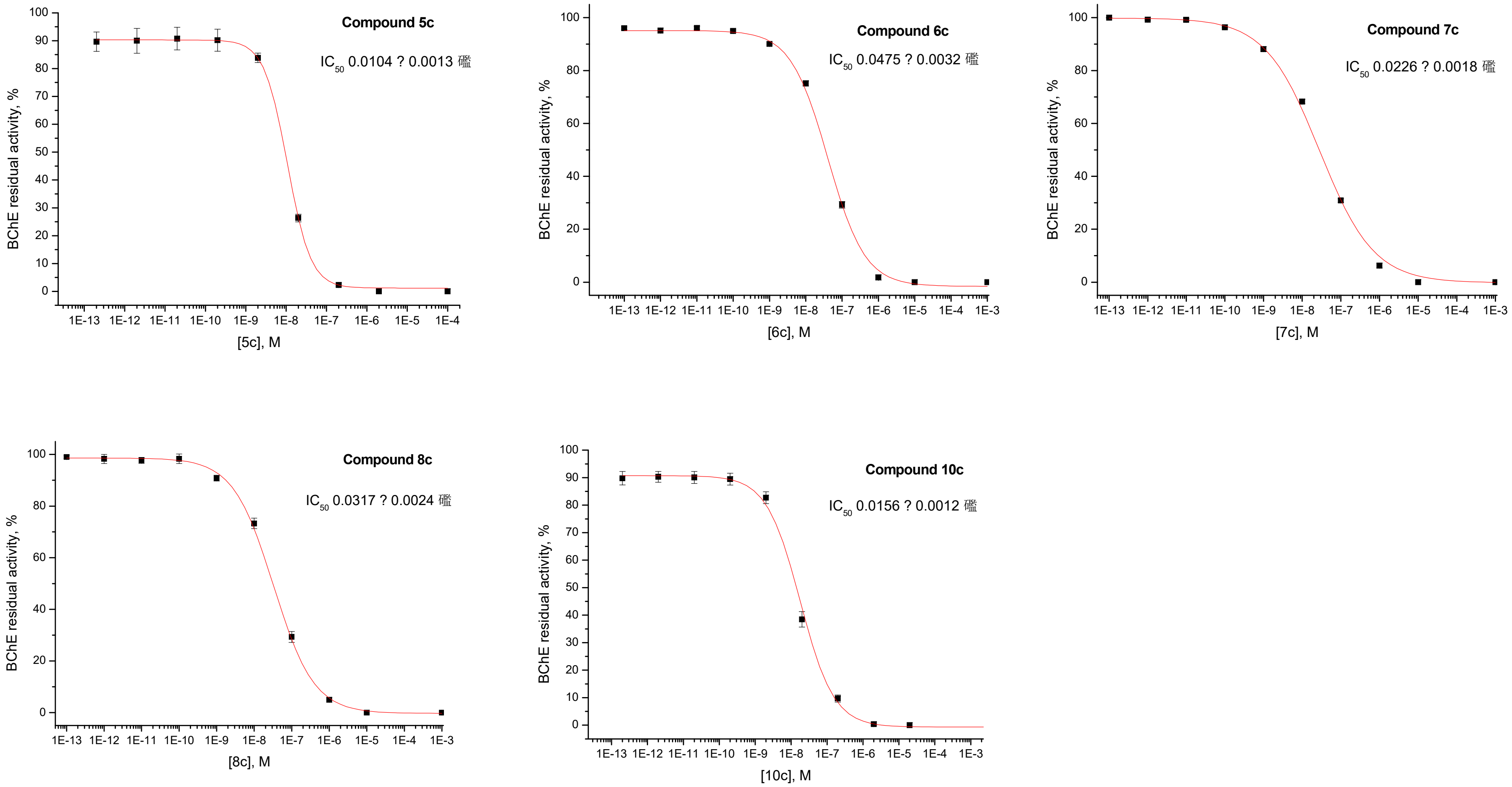

Figure S60. IC_{50} values for BChE inhibition by compounds **5c,6c,7c,8c and 10c** (Mean ± SEM, n = 3)

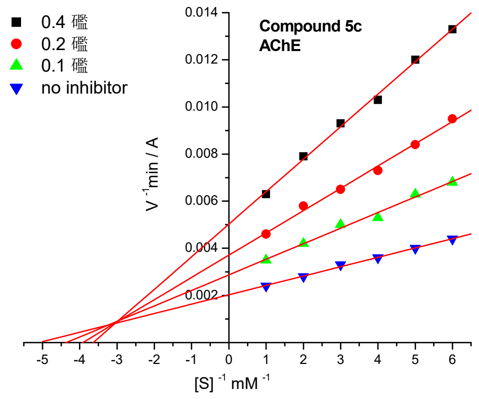

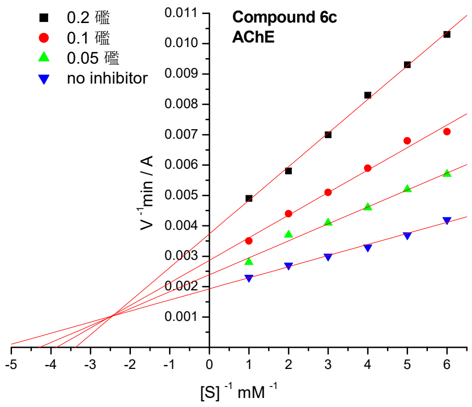

Kᵢ = 0.152±0.012 μM
Kᵢ' = 0.263±0.018 μM

Kᵢ = 0.104 ± 0.004 μM
Kᵢ' = 0.213 ± 0.012 μM

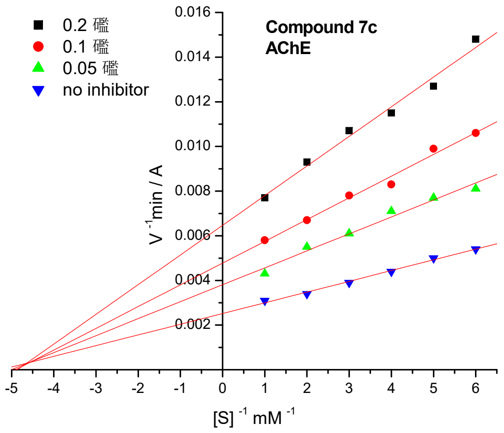

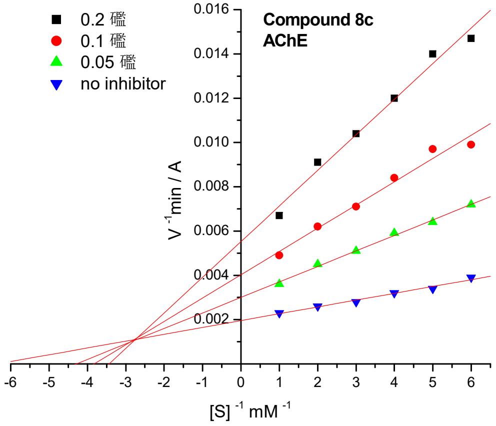

Kᵢ = 0.125 ± 0.003 μM
Kᵢ' = 0.134 ± 0.009 μM

Kᵢ = 0.0536 ± 0.0025 μM
Kᵢ' = 0.0998 ± 0.0058 μM

Figure S61. Lineweaver-Burk double-reciprocal plots of steady state inhibition of AChE by compounds **5c**,**6c**,**7c**,**8c**. Each plot indicates mixed-type inhibition.

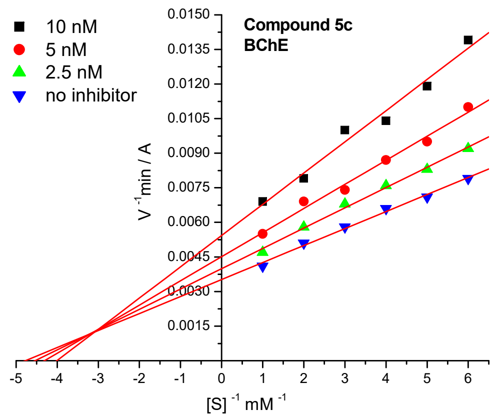

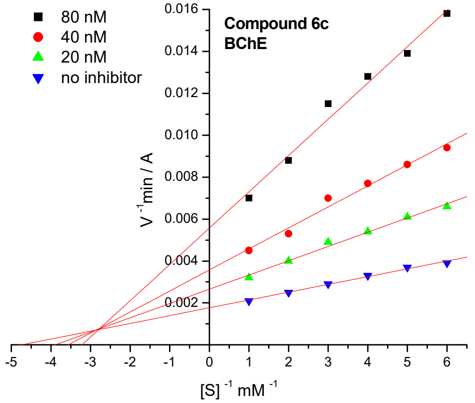

K_i = 0.0106±0.0007μM
K_i' = 0.0171±0.0013 μM

K_i = 0.0188 ± 0.0011 μM
K_i' = 0.0314 ± 0.0025 μM

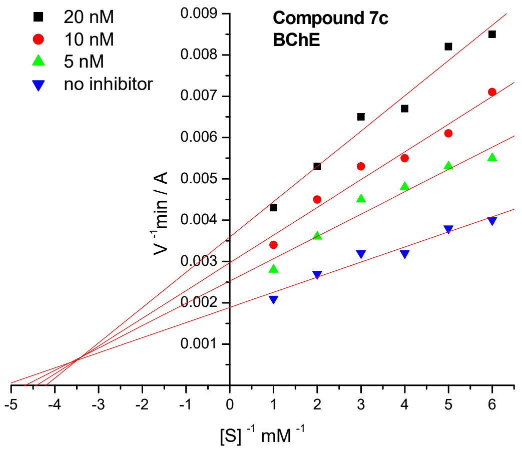

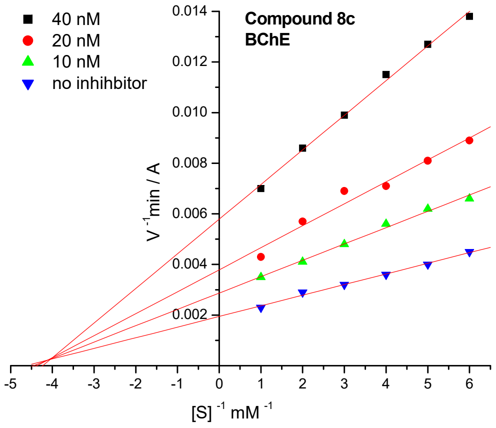

K_i = 0.0173 ± 0.0006 μM
K_i' = 0.0243 ± 0.0001 μM

K_i = 0.0177 ± 0.0007 μM
K_i' = 0.0187 ± 0.0013 μM

Figure S62. Lineweaver-Burk double-reciprocal plots of steady state inhibition of BChE by compounds **5c**,**6c**,**7c**,**8c**. Each plot indicates mixed-type inhibition.

Table S1. pKa values and charges of the investigated compounds.

Compound	Tacrine N	Spacer N	Ar-OH	Charge of the Molecule
**5a**	7.5	-	8.3	+1
**6a**	7.5	-	-	+1
**7a**	7.5	-	7.3	+1
**10a**	7.5	8.4	9	+2
